# Imputing missing RNA-sequencing data from DNA methylation by using a transfer learning–based neural network

**DOI:** 10.1093/gigascience/giaa076

**Published:** 2020-07-10

**Authors:** Xiang Zhou, Hua Chai, Huiying Zhao, Ching-Hsing Luo, Yuedong Yang

**Affiliations:** School of Data and Computer Science, Sun Yat-sen University, 132 East Waihuan Road, Guangzhou 510006, China; School of Data and Computer Science, Sun Yat-sen University, 132 East Waihuan Road, Guangzhou 510006, China; Sun Yat-sen Memorial Hospital, Sun Yat-sen University, 107 Yan Jiang West Road, Guangzhou 510120, China; School of Data and Computer Science, Sun Yat-sen University, 132 East Waihuan Road, Guangzhou 510006, China; School of Data and Computer Science, Sun Yat-sen University, 132 East Waihuan Road, Guangzhou 510006, China; Key Laboratory of Machine Intelligence and Advanced Computing (Sun Yat-sen University), Ministry of Education, 132 East Waihuan Road, Guangzhou 510006, China

**Keywords:** RNA-seq imputation, DNA methylation, transfer learning, neural network

## Abstract

**Background:**

Gene expression plays a key intermediate role in linking molecular features at the DNA level and phenotype. However, owing to various limitations in experiments, the RNA-seq data are missing in many samples while there exist high-quality of DNA methylation data. Because DNA methylation is an important epigenetic modification to regulate gene expression, it can be used to predict RNA-seq data. For this purpose, many methods have been developed. A common limitation of these methods is that they mainly focus on a single cancer dataset and do not fully utilize information from large pan-cancer datasets.

**Results:**

Here, we have developed a novel method to impute missing gene expression data from DNA methylation data through a transfer learning–based neural network, namely, TDimpute. In the method, the pan-cancer dataset from The Cancer Genome Atlas (TCGA) was utilized for training a general model, which was then fine-tuned on the specific cancer dataset. By testing on 16 cancer datasets, we found that our method significantly outperforms other state-of-the-art methods in imputation accuracy with a 7–11% improvement under different missing rates. The imputed gene expression was further proved to be useful for downstream analyses, including the identification of both methylation–driving and prognosis-related genes, clustering analysis, and survival analysis on the TCGA dataset. More importantly, our method was indicated to be useful for general purposes by an independent test on the Wilms tumor dataset from the Therapeutically Applicable Research to Generate Effective Treatments (TARGET) project.

**Conclusions:**

TDimpute is an effective method for RNA-seq imputation with limited training samples.

## Background

The recent development of molecular biology and high-throughput technologies facilitates the simultaneous measurement of various biological omics data such as genomics, transcriptomics, epigenetics, proteomics, and metabolomics for a single patient. Compared with single-omics analysis, integrative analyses of multi-omics data provide comprehensive insights into cancer occurrence and progression and thus strengthen our ability to predict cancer prognosis and to discover various levels of biomarkers. However, owing to limitations of experimental techniques and relatively high costs for measuring the multi-omics data, most samples are not measured with all types of omics data and lack 1 omics data type (called “block missing”). This problem is prevalent in publicly available multi-omics datasets including The Cancer Genome Atlas (TCGA). Because gene expression affects clinical outcomes or phenotypes more directly than molecular features at the DNA level such as DNA methylation and genetic variants [1], we focused on gene expression data imputation from DNA methylation data.

When the missing data happens at random positions in single-omics data, the missing data can be imputed by traditional methods, such as singular value decomposition imputation (SVD) and k-nearest neighbor (KNN) [2]. However, these methods do not perform well when the entire set of RNA sequencing (RNA-seq) data is missing. To address this issue, Voillet et al. developed a multiple hot-deck imputation approach to impute missing rows in a multi-omics dataset for multiple factor analysis [3]. Imbert et al. used multiple hot-deck imputations to improve the reliability of gene network inference [4]. In these 2 methods, they measured the similarities to cases in a standard database and fixed the missing values according to the case with the highest similarity. Because it is easy for the most similar case to be affected by random fluctuations in its neighbors, Dong et al. developed the trans-omics block missing data imputation (TOBMI) method by using a KNN-weighted method to impute messenger RNA–missing samples, where the similarity was measured by similarities in DNA methylation data [5]. Obviously, these methods depend strongly on the available neighbors and are of limited accuracy owing to the relatively small sample sizes of specific cancer datasets. More importantly, they cannot capture information from other related cancer datasets. Recently, least absolute shrinkage and selection operator (Lasso) penalized regression was used to predict gene expression using genetic variants [6] and DNA methylation [7], respectively. However, these linear methods are still limited in their ability to capture the non-linear relations between genomic variables.

In recent years, the deep neural network technique has demonstrated its superiority for modeling complex nonlinear relationships and enjoys scalability and flexibility. For gene expression imputation or prediction, many deep learning models have also been proposed. Chen et al. built a multilayer feedforward neural network to predict the expression of target genes from the expression of ~1,000 landmark genes [8]. With the ability to recover partially corrupted input data, denoising autoencoder (DAE) was used to impute missing values in single-cell RNA-seq data [9–11]. Xie et al. constructed a similar deep model to infer gene expression from genotypes of genetic variants [12]. On the basis of a convolutional neural network (CNN), Zeng et al. used promoter sequences and enhancer-promoter correlations to predict gene expression [13]. One obstacle for using these deep learning models with multi-omics datasets is the high dimensionality (>20,000 features) in omics data despite a small sample size (<1,000). Even TCGA has only hundreds of samples for each cancer type. Thus, it is hard to train an accurate model with millions of parameters in deep learning architecture.

In such scenarios, transfer learning is usually considered as a promising method, in which parameters trained for a task with a large amount of data are reused as the initialization parameters for a similar task with limited data [14]. In the computer vision community, a common strategy is to pretrain the CNN with ImageNet [15] and then fine-tune its last few layers or all layers (depending on the size of the target dataset) for the target tasks. This pretraining approach has achieved state-of-the-art results on many tasks including object detection [16], image segmentation [17], image classification [18], and action recognition [19]. Yosinski et al. pointed out the relationship between network structure and the transferability of features [20]. They showed that the deep features transition from general to specific along the network, and transferring higher layers results in a significant drop in performance because the features are more specific to source datasets.

For the omics data analysis of cancers, the transfer learning strategy has been applied to different tasks. Li et al. built a pan-cancer Cox model for the prediction of survival time, where 8 cancer types were combined to assist the training of a target cancer dataset [14]. Yousefi et al. used samples from uterine corpus endometrial carcinoma and ovarian serous carcinoma to augment the target breast cancer dataset to improve the prediction of clinical outcomes [21]. Hajiramezanali et al. used information from head and neck squamous cell carcinoma to subtype lung cancer [22]. Based on the assumption that different types of cancer may share common mechanisms [23, 24], transfer learning is becoming a useful approach for the prediction of missing data by learning from the data of different cancer types.

**Figure 1:**
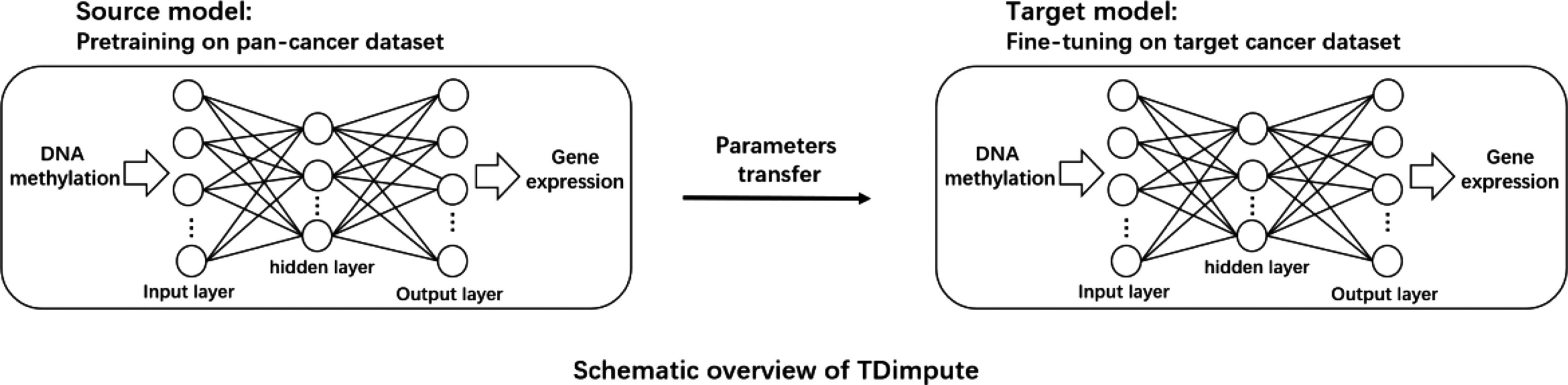
The architecture of a transfer learning–based neural network (TDimpute) for imputing missing gene expression values in a multi-omics dataset. The neural network: DNA methylation data are transformed into gene expression data and the root mean squared error (RMSE) between the actual output and desired output is minimized. Transfer learning: a pan-cancer dataset is used to train the general imputation model for multiple cancers, which is specifically tuned for each type of cancer.

In the present study, we propose a new method to use a transfer learning–based neural network for imputing gene expression from DNA methylation data, namely, TDimpute. Specifically, we first train a neural network based on the pan-cancer dataset to build a general imputation model for all cancers, which is then transferred to target cancer types (Fig. 1). To the best of our knowledge, this is the first study to use transfer learning for the imputation of gene expression from DNA methylation. The method was shown to be superior to other methods to recover gene expressions for 16 cancer types (see Table 1) in TCGA at 5 different missing rates. Our imputed gene expressions were further proven useful with the identification of methylation-driving genes, prognosis-related genes, clustering analysis, and survival analysis through the validations on the TCGA dataset and independent test of the Wilms tumor dataset from the Therapeutically Applicable Research to Generate Effective Treatments (TARGET) project.

**Table 1:** TCGA cancer types and their sample sizes sorted by size, with the first 16 types used for test

Cancer	Full name	Dataset size
BRCA	Breast adenocarcinoma	867
THCA	Thyroid carcinoma	562
HNSC	Head and neck squamous cell carcinoma	541
LGG	Brain lower grade glioma	541
PRAD	Prostate adenocarcinoma	532
LUAD	Lung adenocarcinoma	477
SKCM	Skin cutaneous melanoma	472
BLCA	Bladder urothelial carcinoma	424
LIHC	Liver hepatocellular carcinoma	416
LUSC	Lung squamous cell carcinoma	378
STAD	Stomach adenocarcinoma	371
KIRC	Kidney renal clear cell carcinoma	342
CESC	Cervical squamous cell carcinoma and endocervical adenocarcinoma	308
COAD	Colon carcinoma	297
KIRP	Kidney renal papillary cell carcinoma	297
SARC	Sarcoma	262
UCEC	Uterine corpus endometrial carcinoma	172

## Data Description

### Datasets and preprocessing

#### TCGA

We obtained the data of 33 cancer types from TCGA using the R package TCGA-assembler [25], including RNA-seq gene expression data (UNC IlluminaHiSeq_RNASeqV2_RSEM), DNA methylation data (JHU-USC HumanMethylation450), and clinical information with follow-up and tumor–node–metastasis (TNM) cancer stages [26]. Originally, 20,531 genes and 485,577 methylation sites were collected. After the exclusion of genes with zero values in the RNA-seq data across all samples, 19,027 genes remained. The gene expressions were converted by ${\log _2}( {G + 1} )$, where *G* is the raw gene expression value. For DNA methylation data, we excluded methylation sites with “NA” values, and 269,023 methylation sites remained. By further removing sites with small variances (<0.05) over all samples, 27,717 CpG sites were kept. Here, for evaluating all imputation methods we kept only samples having both RNA-seq and DNA methylation data. Finally, the dataset contains 8,856 samples with expression data for genes and methylation values of 33 cancers, namely, TCGA dataset. To keep enough sample size for downstream analyses, we selected cancer types containing >200 samples with complete DNA methylation, gene expression, and clinical data, leading to 16 cancer types for the test (see Table 1).

#### TARGET

Apart from TCGA, we compiled another dataset developed from the TARGET project. We chose the Wilms tumor (the most common type of childhood kidney cancer) that has the smallest sample size. Here, the DNA methylation data were downloaded from the TARGET Data Matrix [27], and their corresponding RNA-seq data (RSEM estimated read counts) were downloaded from the UCSC Xena [28]. The data were normalized into the same distribution as TCGA by means of quantile normalization [29]. Finally, we obtained 118 samples with complete gene expression data, methylation data, and clinical data, which were randomly split into training and test datasets with a proportion of 1:1.

## Analyses

### Comparisons on the imputation accuracy

**Figure 2:**
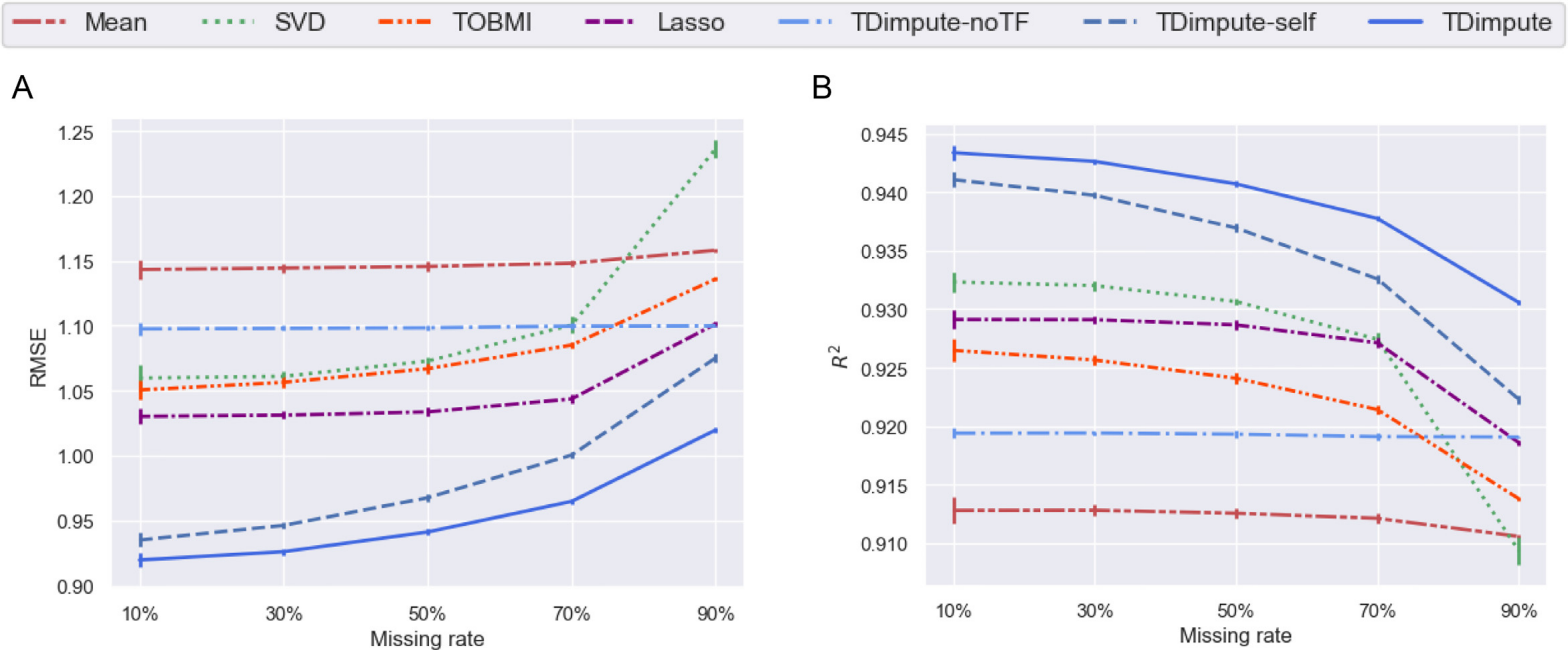
Imputation accuracy of each imputation method. Results were averaged across 16 imputed cancer datasets. (A) RMSE values of each method. (B) The squared Pearson correlation coefficient (${{\boldsymbol{R}}^2}$) between each sample of the imputed data and the original full data. TDimpute-self indicates TDimpute trained and predicted on the target cancer dataset. TDimpute-noTF indicates TDimpute trained on the pan-cancer dataset (excluding the target cancer) and predicted on the target cancer dataset. The error bar shows the standard deviation.

The imputation methods were evaluated by the mean values of the root mean square error (RMSE), mean absolute error (MAE), and the squared Pearson correlation coefficient (${R^2}$) across 16 cancer datasets. After selecting one portion (i.e., 1.0 minus the missing rate) of samples for constructing/fine-tuning the models, the models were then applied to the remained samples. As shown in Fig. 2A (Fig S2 for each dataset), Lasso [7] achieved similar but consistently lower RMSE than TOBMI [5], which indicated that penalized regression was better able to predict the regulation between methylation and gene expression. The SVD method [2] performed worse, demonstrating a slow increase of RMSE from 1.06 to 1.10 as the missing rates changed from 10% to 70%, but then a sharp increase to 1.24 that was even higher than the result by the Mean method. Overall, the Mean method had the worst performance, which coincided with the trend in the previous study [5]. By comparison, TDimpute-self (indicates TDimpute trained and predicted on the target cancer dataset) without using transfer learning yielded 2–9% lower RMSE than Lasso from the highest (90%) to lowest missing rate (10%). The small improvement at the highest missing rate is because the limited training data cannot provide enough information for the model to learn the methylation-expression correlations. This performance degradation at the highest missing rate is reduced by TDimpute, which yielded 7% lower RMSE than Lasso, indicating the advantage of using transfer learning over the non-transfer method. TDimpute-noTF, as a general model, was trained on the pan-cancer dataset (excluding the target cancer). The model did not use information from the target cancer and thus showed a constant performance. It did not perform well but produced lower imputation RMSE than the Mean method. The RMSE was even lower than that of SVD and TOBMI when the missing rate was >70%. TDimpute, a further transfer learning on the target cancer from TDimpute-noTF, decreased the RMSE by 7–16% over TDimpute-noTF. These results confirmed the power of our TDimpute method in transferring knowledge from other cancer types to improve imputation performance. We also noted that SVD, Lasso, and TOBMI had close to constant RMSE values at missing rates from 10% and 70%, indicating that tripling the sample sizes did not contribute much to increasing the imputation accuracies. By comparison, the deep learning methods, TDimpute and TDimpute-self, decreased the RMSE by 5% and 7%, respectively, indicating the potential for further improvement with an increase of sample sizes in the future.

Because RMSE is sensitive to outliers, we also evaluated the imputation performance with MAE, which is less affected by anomalous outliers. As shown in Fig S1, TDimpute consistently performed the best at all missing rates, with MAE of 0.616–0.685. Slightly differently, TOBMI's performance became similar to that of Lasso, with lower MAE at 10% while slightly higher MAE at missing rates >50%.

When measured by the squared correlation (${R^2}$) between the imputed and actual values by each sample (Fig. 2B, Fig. S3 for each dataset), approximatively the same trends could be observed for all methods. Differently, SVD ranked third except at a missing rate of 90%, where SVD had the lowest ${R^2}$ of 0.909. The imputations by the mean gene expressions kept the lowest performance. Hereafter, we focus on the comparison with SVD, Lasso, and TOBMI methods.

### Effects on the methylation-expression correlations and the identification of methylation-driving genes

**Figure 3:**
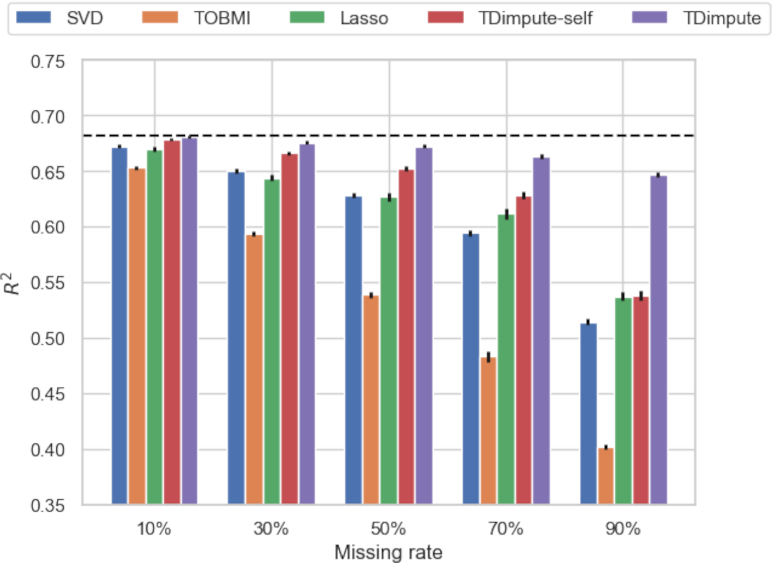
The mean correlations (${{R}^2}$) of the top 100 CpG-gene pairs over 16 cancer datasets by 5 imputed methods. Dashed black line indicates the correlations from the actual dataset. Error bars show the standard error of the mean.

For a multi-omics dataset, a proper imputation method should preserve the correlation structures between different types of omics. Because the most correlated CpG-gene pairs play the most important roles, we only compared the effect of imputation methods by the mean ${R^2}$ of the top 100 CpG-gene pairs from full datasets. As shown in Fig. 3 (Fig. S4 for each dataset), all imputation methods caused decreases in the correlations, and differences increase with the missing rates. In general, TDimpute had the highest recovery power for the methylation-expression correlation, with ${R^2}$ values close to the actual correlation (${R^2}$ = 0.68). The performance is followed by TDimpute-self. At a missing rate of 90%, TDimpute-self by using a single dataset had a large decrease in ${R^2}$ to the same level with Lasso. Although TOBMI performed better than SVD according to RMSE, TOBMI consistently had the lowest correlations, likely because TOBMI imputes gene expressions simply by nearest neighbors in DNA methylations, which obscures the complex methylation-expression relations.

We further investigated whether the preservation of correlations can obtain better performance in identifications of methylation-driving genes. As presented in Table 2 and Table S2.1, TDimpute consistently showed superiority in preserving methylation-driving genes from original data with the highest PR-AUC (area under precision-recall curve) and top 100 overlaps, respectively. TDimpute-self ranked second in selecting methylation-driving genes, followed by SVD, Lasso, and TOBMI. Compared with TDimpute-self, TDimpute achieved 0.3–34% and 1–49% improvements for PR-AUC and overlap ration, respectively. The improvement was especially pronounced at high rates of missing data. These results confirm the consistency between correlation preservation and methylation-driving gene identification.

**Table 2:** Mean PR-AUC over 16 cancers for recovering methylation-driving genes according to the imputed relative to the actual gene expression data

Missing rate	SVD	TOBMI	Lasso	TDimpute-self	TDimpute
10%	0.972	0.971	0.965	0.980	0.983
30%	0.867	0.867	0.842	0.908	0.927
50%	0.737	0.703	0.702	0.807	0.848
70%	0.587	0.521	0.565	0.678	0.749
90%	0.391	0.308	0.390	0.397	0.601

Mean performance across 16 imputed cancer datasets is reported. TDimpute had the best results in all comparisons. All differences were statistically significant (paired*t*-test, *P*-value < 0.05) between TDimpute and other methods.

The PR-AUC and overlap of top 100 methylation-driving genes per cancer dataset are detailed in Tables S1 and S2.2, respectively.

### Effects on the identification of prognosis-related genes

We investigated the recovery power of different imputation methods on the identification of significantly prognosis-related genes. To evaluate the selected genes, we compared the PR-AUC and the overlaps between the top 100 genes identified from the imputed and the actual data in Table 3 and Table S4.1, respectively. We found that TDimpute consistently achieved the best performance in all missing rates, with 2–28% higher PR-AUC values and 4–54% greater number of overlapped genes than those by the TOBMI method. Lasso achieved lower values than TOBMI, except at a missing rate of 90%, where Lasso slightly overtook TOBMI. The high agreements and overlap ratios indicate that the imputed gene expressions by TDimpute are more relevant to the clinical outcome.

**Table 3:** The mean PR-AUC for recovering prognosis-related genes according to the imputed relative to the actual gene expression data.

Missing rate	SVD	TOBMI	Lasso	TDimpute-self	TDimpute
10%	5.53*	5.83	5.55	5.71	5.91
30%	3.46*	4.08	3.71*	4.22	4.25
50%	2.08*	2.74	2.42*	2.94	3.06
70%	1.14*	1.60*	1.33*	1.87	2.03
90%	0.56*	0.47*	0.49*	0.89*	1.14

*Statistical significance (paired *t*-test, *P*-value < 0.05) between TDimpute and other methods. TDimpute had the best results in all comparisons.

The top 100 genes (ranked by *P*-values) were additionally compared with the prognosis-related gene list downloaded from The Human Protein Atlas (THPA) [30] by the enrichment relative to random selections. As reported in Table 4, TDimpute achieved the largest enrichment factors (see Methods section for definition), indicating its ability to identify the really validated prognosis-related genes. At missing rates <70%, TOBMI consistently outperformed Lasso and SVD performed the worst. At the missing rate of 90%, all methods except TDimpute performed worse than random selection with enrichment factors <1.0.

**Table 4:** The average enrichment factors of top 100 prognosis-related genes overlapped with the genes collected in the Human Protein Atlas.

Missing rate	SVD	TOBMI	Lasso	TDimpute-self	TDimpute
10%	0.901	0.912	0.912	0.923	0.927
30%	0.711	0.747	0.742	0.768	0.784
50%	0.561	0.595	0.590	0.627	0.652
70%	0.428	0.454	0.449	0.487	0.523
90%	0.286	0.287	0.292	0.311	0.376

All differences were statistically significant (paired *t-*test, *P*-value < 0.05) between TDimpute and other methods. TDimpute had the best results in all comparisons.

The PR-AUC, overlap of top 100 prognostic genes, and the enrichment factors per cancer dataset are detailed in Tables S3, S4.2, and S5, respectively.

### Effects on the performance of clustering analysis and survival analysis

**Figure 4:**
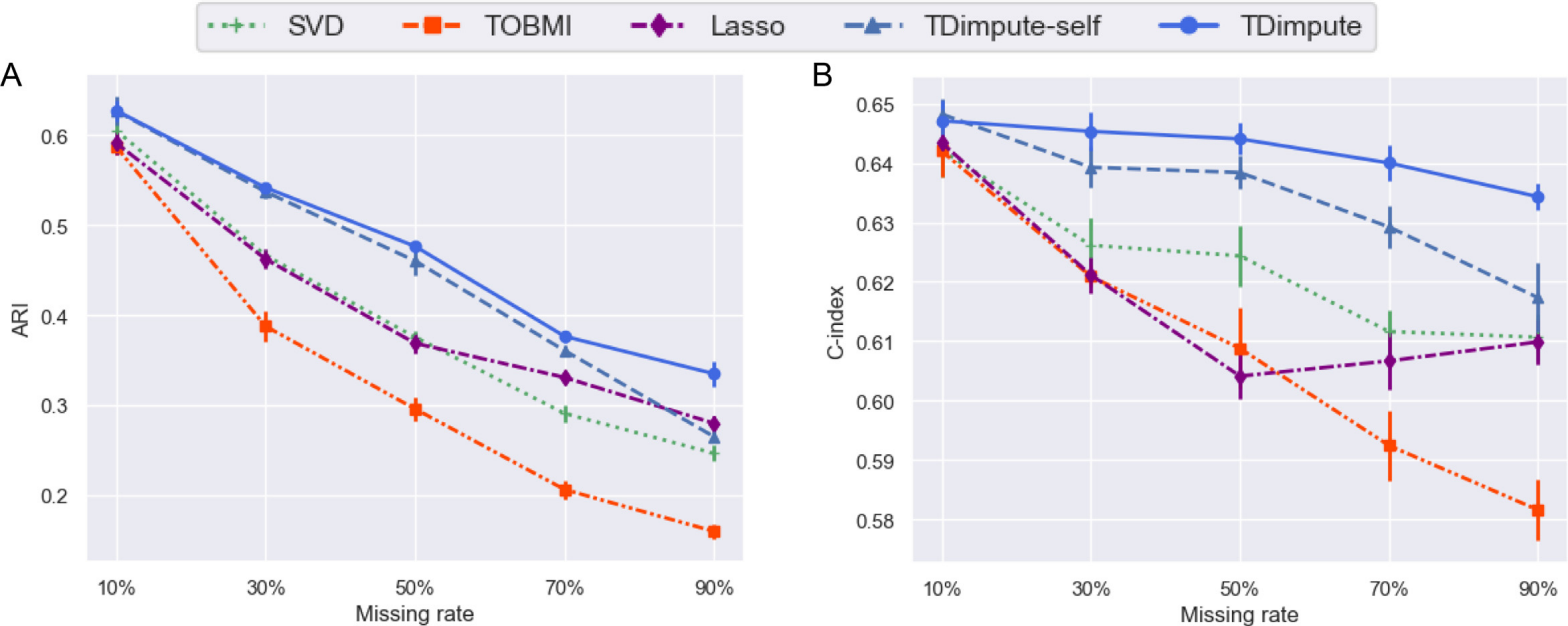
(A) Mean adjusted Rand index (ARI) of the clusters from the imputed and actual data, and (B) mean C-index by survival analyses based on imputed data over 16 cancers. Error bars show the standard error of the mean.

We also evaluated the effects of different imputation methods on clustering analysis and survival analysis. By input of the top 100 prognosis-related genes, the K-means algorithm was used to divide the samples into 2 clusters. Fig. 4A (Fig. S5 for each dataset) shows the adjusted Rand index (ARI) for evaluating the concordance between clusters from the imputed and actual gene expression. For all methods, accuracy decreased with the increase of the missing rate, agreeing with the previous study [10]. As expected, TDimpute achieved the highest clustering concordance among the 5 imputation methods consistently under different missing rates.

A further survival analysis (Fig. 4B, Fig. S6 for each dataset) shows that TDimpute consistently outperformed the competing methods. Interestingly, the C-index was not as sensitive to the missing rates. Even at a missing rate of 90%, imputed data caused a decrease of 2–9% in C-index, much smaller than the decreases of 59–68% in PR-AUC for recovering prognosis-related genes and 45–74% in ARI for clustering analysis. This is likely because the Cox models were trained with contributions from multiple gene features, and thus bad imputations could be avoided. Similar results were also found in previous studies [31, 32]. For reference, the C-index was 0.669 according to TNM typing [26] assigned by clinicians on the basis of tumor phenotypes, indicating the necessity of combining genotype and phenotype for survival analyses in the future.

### Validation on the UCEC from TCGA dataset

As a real data application, we performed survival analysis on the uterine corpus endometrial carcinoma (UCEC) dataset because it has the largest proportion of missing gene expression in TCGA (172 samples with RNA-seq and 267 without). By using the imputed samples, TDimpute achieved the highest C-index of 0.588, compared with TDimpute-self, SVD, TOBMI, and Lasso with C-index of 0.575, 0.55, 0.553, and 0.508, respectively. Fig. S7 shows the survival curves of 2 groups separated by K-means. The 2 groups for TDimpute show a significant difference (*P* = 1.7e−04) in the survival curves according to the log-rank test. By comparison, the *P*-values were 1.1e−03, 1.9e−03, 9.5e−03, and 1.2e−03 for TDimpute-self, Lasso, TOBMI, and SVD, respectively.

### Independent test on the TARGET dataset

**Figure 5:**
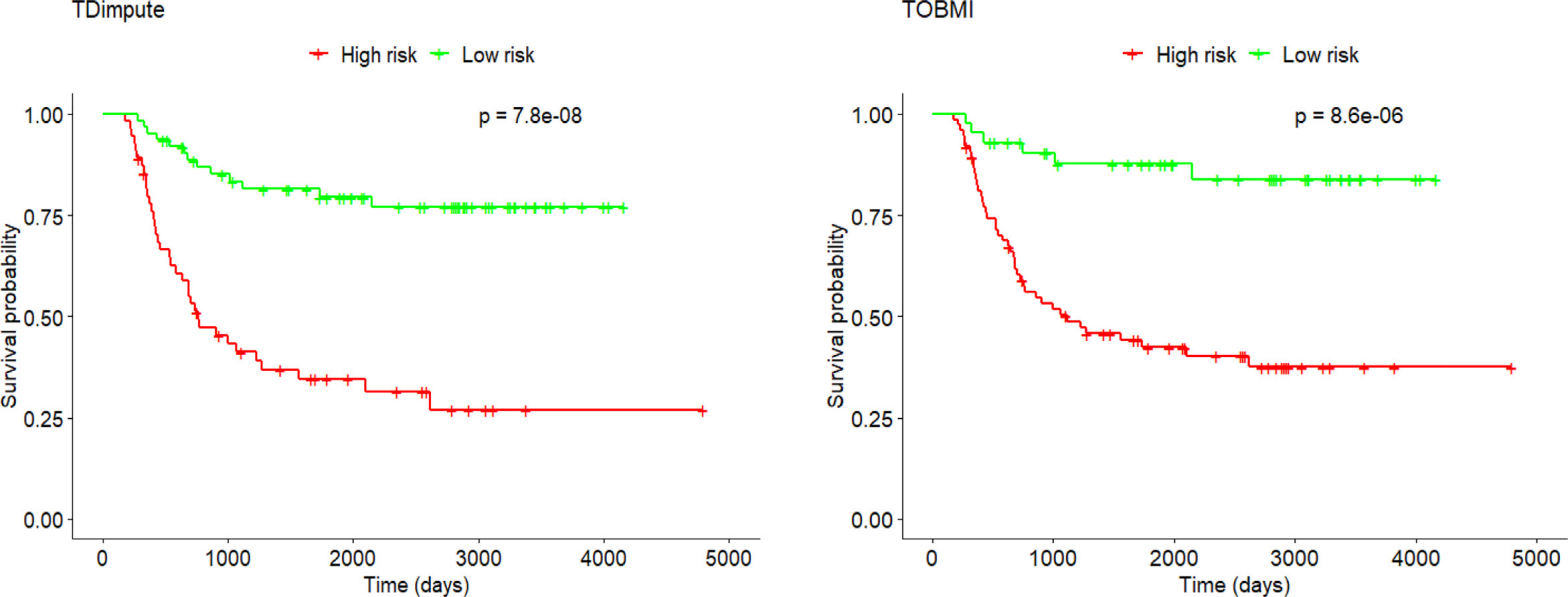
Kaplan-Meier plot for the 2 clusters obtained from the Wilms tumor dataset imputed by TDimpute and TOBMI, respectively. Censored data are marked with a small vertical bar in the graph.

As an independent test beyond TCGA, we selected the smallest Wilms tumor dataset from the TARGET project. By fine-tuning the TCGA pan-cancer model with 59 randomly selected samples (50% of the dataset), the model was tested on the remaining samples. As expected, TDimpute achieved the lowest RMSE of 0.955 (TDimpute-self: 0.98; SVD: 1.064; Lasso: 1.006; TOBMI: 1.018). This demonstrates the generalization of the TCGA-pretrained model on an independent dataset. The K-means method is used to cluster the 118 samples after imputation, and the 2 resulting clusters were used to plot the survival curves. For the Kaplan-Meier survival curves in Fig. 5, TDimpute achieved the best prognostic stratification with *P*-value of 7.81e−08, compared with 4.53e−07, 8.64e−06, 3.80e−03, and 2.99e−02 for TDimpute-self, TOBMI, Lasso, and SVD, respectively. TDimpute achieved the highest C-index of 0.592, compared with 0.591, 0.523, 0.558, and 0.501 for TDimpute-self, SVD, TOBMI, and Lasso, respectively.

## Discussion

In this study, we used TDimpute to perform missing gene expression imputation by building a highly nonlinear mapping from DNA methylation to gene expression data. Owing to the limited size of cancer datasets in TCGA, we used transfer learning to capture the commonalties in the pan-cancer dataset for pretraining parameters. We compared TDimpute with and without transfer learning, Lasso, SVD, and TOBMI methods by RMSE, MAE, and correlation ${R^2}$, and methylation-expression correlations. Because the main task of imputations is to recover biologically meaningful gene expression data for downstream analysis, we also evaluated the effect of imputations on the identification of methylation-driving genes, prognosis-related genes, clustering analysis, and survival prognosis. It is worth noting that although only methylation and gene expression data are illustrated in this study, our framework is capable of incorporating other omics data.

Experimental results on 16 cancer datasets confirmed that our TDimpute method without transfer learning already outperformed the Lasso, SVD, and TOBMI methods in different evaluation metrics. By including transfer learning, the TDimpute method can further improve its performance especially at high rates of missing data. In addition, the ranking of the Lasso, SVD, and TOBMI methods on imputation accuracy (RMSE, MAE, and correlation ${R^2}$) does not strictly agree with their performance in preservation of methylation-expression correlations, clustering analysis, and survival prognosis, but our TDimpute method consistently performed the best in both imputation accuracy and downstream analyses.

Besides the good performance in imputation accuracy and downstream analyses, another main advantage of our proposed method is its computational efficiency and convenience. Based on GPU acceleration, our TDimpute method is capable of processing a large-scale pan-cancer dataset including tens of thousands of samples and hundreds of thousands of features, while Lasso, TOBMI, and SVD are hindered by poor scalability due to the computational complexity of distance matrix computations and SVD operations. Based on the pretrained model, a transfer learning framework can also accelerate the training process on the target dataset.

In previous study of genome-wide association analysis without directly measured gene expression [6, 33], gene expressions could be imputed from genomic data to perform transcriptome-wide association analysis that can reduce the multiple-testing burden and identify associated genes. Recently, many studies have been proposed to impute gene expressions from genomic data, or even from pathology images [34]. In the future, the predicted gene expression from other omics data, such as genomics, pathology, or/and radiomics, can also be integrated in epigenome-wide association studies [35].

Future work can focus on reducing the amount of model parameters and integrating more related training samples. Because we only used the correlations between omics for imputations, one possible direction is to leverage prior knowledge of the gene-gene interaction network. The known relationships between variables/genes have a demonstrated ability to significantly reduce model parameters by enforcing sparsity on the connections of a neural network [36]. The performance of this approach is dependent on the quality of the gene-gene networks, and more investigation needs to be done in this direction.

In the preprocessing step, we removed sites with low-variance DNA methylation over all samples, which might remove some sites that are useful for specific cancer types. To test whether the cancer-specific CpG sites from the pan-cancer dataset are necessary for gene expression prediction, we updated the neural networks by taking the top 20,000 variable CpG sites specific to each cancer as auxiliary input (Fig. S8). Fig. S9 shows that the improvement is relatively limited with only 0.4–1.4% decrease of RMSE, compared to the network without including the cancer-specific sites. These results indicate that the CpG sites with high variances (>0.05) over all samples are adequate for predicting gene expression across different cancers.

## Methods

### Network architecture and model training

#### Neural network architecture

As shown in Fig. 1, TDimpute is a 3-layer neural network with sizes of [27717, 4000, 19027]. The nodes between layers are fully connected and the sigmoid activation function is adopted. The loss function for training is the RMSE, which minimizes the difference between the experimentally measured and predicted gene expression value:

(1) \begin{equation*} \mathrm{RMSE}\left( {y,{{\mathrm{y}}^0}} \right){\mathrm{\ }} = \sqrt {\frac{1}{N}\mathop \sum \nolimits_{i = 1}^N {{\left({y_i} - {\mathrm{y}}_{\mathrm{i}}^0\right)}^2}}, \end{equation*}

where $y_i^0\ $and ${y_i}$ are the experimentally measured and predicted expression value for gene *i*, and *N* is the dimension of the output vector (i.e., the number of genes). The network can be considered as a highly nonlinear regression function that maps DNA methylation data (input) to gene expression data (output).

The network was trained using Adam optimizer with default parameters (learning rate set as 0.0001) [37]. The method was implemented with TensorFlow [38]. All the codes and pretrained pan-cancer models are available on Github [39].

#### Transfer learning setting

To train the prediction model for 1 target cancer in TCGA, the datasets of other cancer types were combined to generate a multi-cancer model that was then fine-tuned by the target cancer data (Fig. 1). The data of the target cancer were excluded to train the multi-cancer model as we needed to remove different portions of the data for the target cancer to evaluate our imputation model. During the fine-tuning process, we reused the network architecture and all the initialization parameters from the pretrained model. Here, we did not freeze any layer because this demonstrated better performance.

#### Hyper-parameter tuning

For our neural network, we selected the BRCA dataset to optimize all hyper-parameters by RMSE through 5-fold cross-validation. Here, we optimized the hyper-parameters based on the BRCA dataset and then applied the optimal hyper-parameters to all cancer types. Based on the pan-caner dataset (excluding the BRCA dataset), we first optimized the pretrained model to determine the architecture of the neural network, i.e., the number of hidden layers, the hidden layer size, and the epochs to stop training. Details on the performance analysis for each hyper-parameter are provided in Table S6 and Fig. S10 in the supplementary material. For the fine-tuning process on the BRCA dataset, the only hyper-parameter we need to choose is the training epoch because the network architecture was determined by the pretrained model. Fig. S11 shows the convergence process of different levels of missingness on the validation dataset of BRCA.

Finally, we selected the following hyper-parameters for the pan-cancer model: 1 hidden layer (selected from 1 and 3) including 4,000 nodes (from 500, 1,000, 2,000, 4,000, and 5,000), Sigmoid activation function (from Tanh, Relu, and Sigmoid), epochs of 300 (from 50, 100, 150, 300, and 500), and batch size of 128. For the fine-tuning stage, 150 epochs (from 50, 100, 150, 300, and 500) were used and the batch size was set as 16 because of small sample sizes under large missing rates. Dropout was not used because it decreased the performance [40].

### Training and testing datasets

In the transfer learning for each target cancer type of TCGA, 32 cancer types in the TCGA dataset (except the target cancer type) were used as the source domain dataset for pretraining a model. During the test, to simulate the performance under different missing rates, we used 5 fractions (10%, 30%, 50%, 70%, 90%). At each missing rate, we used the sample function (without replacement) in Pandas [41] to get 1 fraction (i.e., 1.0 minus the missing rate) of the target cancer samples as training set and the remaining samples as testing set with gene expression removed. On the training set, we fine-tuned the models that were then tested on the testing set to predict the gene expression. The predicted values were then compared with the actual values to evaluate the model performance. To remove random fluctuations, we used a bootstrapping strategy to repeat this process for 5 times and reported the mean performance.

### Performance comparison

We compared our method with other imputation methods, including Lasso [7], TOBMI [5], and SVD methods [2]. The default or suggested parameters were used for these methods. We also evaluated the performance by using the mean expression of each gene for reference.

### Preservation of methylation-expression correlations and methylation-driving genes

Here, we used the squared Pearson correlation coefficient ${R^2}$ to evaluate the effect of imputation methods on the correlations between DNA methylation and gene expression. For each gene, we only considered the CpG site with the strongest correlation. Based on the methylation-expression regulation, many studies have been conducted to identify cancer-related DNA methylation-driving (hyper- and hypomethylated) genes [42]. Hence, we also evaluated the effects of imputation methods on the identification of methylation-driving genes. The methylation-driving genes (i.e., significantly correlated CpG-gene pairs) were defined with the ${R^2} \ge 0.5$ and $\mathrm{FDR}-q{\mathrm{\ }} \le {\mathrm{\ }}0.05$. According to the correlated pairs from the original gene expression data, we can compute the area under precision-recall curve (PR-AUC). We also computed the overlap between the top 100 ranked genes identified from the imputed datasets and original full datasets.

### Preservation of prognosis-related genes

A common task in the analysis of gene expression data is the identification of prognostic genes. To evaluate the effect of different imputation methods on the identification of potentially prognosis-related genes, we built univariate Cox proportional hazard regression models to select statistically significant genes correlated with overall survival. With the Cox model, each gene is assigned a *P*-value describing the significance of the relation between the gene and the target cancer, and genes with *P*-values $\le $ 0.05 were defined as prognosis-related genes. Similar to the evaluations by the methylation-driving genes, PR-AUC and overlapped top 100 genes were used to evaluate all imputation methods.

In addition, we compared our identified genes with the list of prognosis-related genes from THPA [30] through the enrichment fraction:

(2) \begin{equation*} \mathrm{EF}\ = {\mathrm{\ }}\left( {{N_{{\mathrm{True}}}}/{N_{{\mathrm{selected}}}}} \right)/\left( {{N_{{\mathrm{Active}}}}/{N_{{\mathrm{Total}}}}} \right), \end{equation*}

where ${N_{{\mathrm{True}}}}$ is the number of genes appearing in both THPA and our top ${N_{{\mathrm{selected}}}}$ ranked genes, and ${N_{{\mathrm{Active}}}}$ and ${N_{{\mathrm{Total}}}}\ $ are the number of prognosis-related genes and total number of genes in THPA, respectively.

### Effects on clustering analysis and survival analysis

We evaluated the relations of genes to cancer survival by *P*-value output from the univariate Cox models. By using the top 100 genes, their expression values were used to divide samples into 2 clusters by the K-means method. Clustering performance was assessed by means of the ARI, a measure of agreement between the predicted clustering labels (by imputed datasets) and the actual clustering labels (by original datasets). We further made survival predictions with significantly related genes (*P*$\le $ 0.05) by using the ridge regression regularized Cox model implemented through the glmnet package [43] in R, a model suitable for fitting regression models with high-dimensional data. The performance of the Cox model was assessed by the Harrell concordance index (C-index), which measures the concordance between predicted survival risks and actual survival times.

## Availability of Source Code and Requirements

Project name: Transfer learning for imputing missing RNA-seq data from DNA methylation

Project home page: https://github.com/sysu-yanglab/TDimpute

Operating system(s): Platform independent

Programming language: Python

License: MIT

biotoolsID: TDimpute

RRID:SCR_018306

## Availability of Supporting Data and Materials

The datasets and pretrained pan-cancer models supporting the results of this article are available in the Synapse with ID: syn21438134 [44]. Snapshots of our code and data further supporting this work are openly available in the *GigaScience* repository, GigaDB [45].

## Additional Files

**Figure S1**. Mean absolute error of each imputation method. Results were averaged across 16 imputed cancer datasets. TDimpute-self indicates the TDimpute trained and predicted on the target cancer dataset. TDimpute-noTF indicates the TDimpute trained on the pan-cancer dataset (excluding the target cancer) and predicted on the target cancer dataset. The error bars show the standard deviation.

**Figure S2**. RMSE on 16 imputed cancer datasets with different missing rates. The results were averaged over 5 random replicates. The error bars show the standard deviation.

**Figure S3**. The squared Pearson correlation coefficients ${{\boldsymbol{R}}^2}$ between each sample of the imputed data and the original full data on 16 imputed cancer datasets with different missing rates. The results were averaged over 5 random replicates. The error bars show the standard deviation.

**Figure S4**. The squared Pearson correlation coefficients ${{\boldsymbol{R}}^2}$ between gene expression and methylation sites on 16 imputed cancer datasets with different missing rates. The results were averaged over 5 random replicates. Dashed black line is drawn as a reference indicating the correlations from the original full dataset. The error bars show the standard error of the mean.

**Figure S5**. ARI on 16 imputed cancer datasets with different missing rates. The results were averaged over 5 random replicates. The error bars show the standard error of the mean.

**Figure S6**. C-index on 16 imputed cancer datasets with different missing rates. The results were averaged over 5 random replicates. The error bars show the standard error of the mean.

**Figure S7**. Kaplan-Meier plot for the 2 clusters obtained from the UCEC dataset imputed by TDimpute and SVD, respectively.

**Figure S8**. The architecture of transfer learning–based neural network (TDimpute) with cancer-specific CpG sites as auxiliary input. The input at the fine-tuning stage consists of 2 parts: the cancer-specific part takes the highly variable CpG sites from the target cancer as input, and the transfer part takes the commonly variable CpG sites from the pan-cancer dataset as input.

**Figure S9**. RMSE values of each imputation method with top 20,000 CpG sites as input. For SVD, TOBMI, Lasso, and TDimpute-self, the top 20,000 variable cancer-specific CpG sites are used as input, while the input of TDimpute includes both the pan-cancer and cancer-specific CpG sites. Results were averaged across 16 imputed cancer datasets. The error bars show the standard deviation.

**Figure S10**. Loss curves for different hidden layer shape.

**Figure S11**. The loss curves of different missing rates on the validation dataset of BRCA.

**Table S1**. PR-AUC for detecting methylation-driving genes on imputed cancer datasets over 16 cancer types.

**Table S2.1**. Overlap of top 100 methylation-driving genes from imputed dataset and full dataset.

**Table S2.2**. Overlap of top 100 methylation-driving genes between imputed dataset and full dataset over 16 cancer types.

**Table S3**. PR-AUC for detecting significantly prognostic gene on imputed datasets over 16 cancer types.

**Table S4.1**. Overlap of top 100 significantly prognostic genes identified by univariate Cox model between imputed datasets and full datasets.

**Table S4.2**. Overlap of top 100 prognostic genes identified by univariate Cox model between imputed dataset and full dataset over 16 cancer types.

**Table S5**. The enrichment factors of the top 100 ranked genes in the gene list from The Human Protein Atlas across 16 cancer types.

**Table S6**. Hyper-parameter analysis for hidden layer shape, the number of hidden layers, and training epochs on pan-cancer dataset (excluding BRCA dataset).

giaa076_GIGA-D-19-00438_Original_Submission

giaa076_GIGA-D-19-00438_Revision_1

giaa076_GIGA-D-19-00438_Revision_2

giaa076_Response_to_Reviewer_Comments_Original_Submission

giaa076_Response_to_Reviewer_Comments_Revision_1

giaa076_Reviewer_1_Report_Original_Submission Ali Sharifi-Zarchi -- 2/18/2020 Reviewed

giaa076_Reviewer_2_Report_Original_Submission Jiangning Song -- 2/23/2020 Reviewed

giaa076_Reviewer_2_Report_Revision_1 Jiangning Song -- 5/13/2020 Reviewed

giaa076_Supplemental_File

## Abbreviations

ARI: adjusted Rand index; C-index: concordance index; CNN: convolutional neural network; DAE: denoising autoencoder; FDR: false discovery rate; KNN: k-nearest neighbor; LASSO: least absolute shrinkage and selection operator; MAE: mean absolute error; PR-AUC: area under precision-recall curve; RMSE: root mean square error; SVD: singular value decomposition imputation; TARGET: Therapeutically Applicable Research to Generate Effective Treatments; TCGA: The Cancer Genome Atlas; TDimpute: transfer learning–based deep neural network for imputation; THPA: The Human Protein Atlas; TNM: tumor–node–metastasis; TOBMI: trans-omics block missing data imputation; UCSC: University of California Santa Cruz.

## Competing Dnterests

The authors declare that they have no competing interests.

## Funding

This work has been supported by the National Key R&D Program of China (2018YFC0910500), National Natural Science Foundation of China (61772566, U1611261, and 81801132), Guangdong Key Field R&D Plan (2018B010109006 and 2019B020228001), Natural Science Foundation of Guangdong, China (2019A1515012207), and Introducing Innovative and Entrepreneurial Teams (2016ZT06D211).

## Authors' Contributions

Y.Y. and C.L. conceived the study. Y.Y. and X.Z. contributed to the design and development of the model. X.Z. and H.C. implemented the software and led the curation of the datasets. Y.Y., H.Z., and X.Z. led the analytics. All authors wrote and approved the final manuscript.
