# Supplementary material for: Imputing missing RNA-sequencing data from DNA methylation by using a transfer learning–based neural network
Source: Gigascience. 2020 Jul 10;9(7):giaa076. doi: 10.1093/gigascience/giaa076 (PMC7350980; doi:10.1093/gigascience/giaa076)
Supplement: giaa076_GIGA-D-19-00438_Original_Submission [file giaa076_giga-d-19-00438_original_submission.pdf]

GigaScience

Imputing missing RNA-seq data from DNA methylation by using transfer learning based neural network

--Manuscript Draft--

**Manuscript Number:**	GIGA-D-19-00438
**Full Title:**	Imputing missing RNA-seq data from DNA methylation by using transfer learning based neural network
**Article Type:**	Research
**Funding Information:**	National Key R&D Program of China (2018YFC0910500) — Dr. Yuedong Yang<br>National Natural Science Foundation of China (CN) (U1611261, 61772566, and 81801132) — Dr. Yuedong Yang<br>Guangdong Key Laboratory of Innovation Method and Decision Management System (CN) (2018B010109006, 2019B020228001) — Dr. Yuedong Yang<br>Natural Science Foundation of Guangdong Province (CN) (2019A1515012207) — Dr. Hua Chai<br>Startup Foundation for Introducing Talent of Nanjing University of Information Science and Technology (CN) (2016ZT06D211) — Dr. Yuedong Yang
**Abstract:**	As an epigenetic modification, DNA methylation plays an important role in regulating gene expression. Integrative analysis of DNA methylation and gene expression can capture the associations of the two omics, and thus provides a comprehensive view of the molecular basis underlying cancers. However, it is common that one type of omics data is missing due to various limitations in experiments, preventing downstream analyses that need complete dataset. Imputations from one type of omics data to another is becoming important, but current methods mainly focus on single cancer dataset with limited sample size, and thus are limited by their ability to capture information from large pan-cancer dataset. Here, we present a novel transfer learning-based neural network to impute missing gene expression data from DNA methylation data, namely TDimpute. In the method, the pan-cancer dataset from TCGA was utilized to train a general model for all cancers, which was then fine-tuned on the specific cancer dataset for each cancer. By testing on 16 cancer datasets, we found that our method significantly outperforms other state-of-the-art methods in terms of imputation accuracy (7%-11% increase with different missing rates). The imputed gene expression was also validated to be useful for all downstream analyses, including the identification of both DNA methylation-driving and prognosis-related genes, clustering analysis, and survival analysis. Our method was further proved based on the Wilms tumor dataset from TARGET cancer project.
**Corresponding Author:**	Yuedong Yang<br>Sun Yat-Sen University<br>Guangzhou, Guangdong CHINA
**Corresponding Author Secondary Information:**	
**Corresponding Author's Institution:**	Sun Yat-Sen University
**Corresponding Author's Secondary Institution:**	
**First Author:**	Xiang Zhou
**First Author Secondary Information:**	
**Order of Authors:**	Xiang Zhou<br>Hua Chai

Powered by Editorial Manager® and ProduXion Manager® from Aries Systems Corporation

	Huiying Zhao
	Ching-Hsing Luo
	Yuedong Yang
**Order of Authors Secondary Information:**	
**Additional Information:**	
**Question**	**Response**
Are you submitting this manuscript to a special series or article collection?	No
**Experimental design and statistics**<br><br>Full details of the experimental design and statistical methods used should be given in the Methods section, as detailed in our Minimum Standards Reporting Checklist. Information essential to interpreting the data presented should be made available in the figure legends.<br><br>Have you included all the information requested in your manuscript?	Yes
**Resources**<br><br>A description of all resources used, including antibodies, cell lines, animals and software tools, with enough information to allow them to be uniquely identified, should be included in the Methods section. Authors are strongly encouraged to cite Research Resource Identifiers (RRIDs) for antibodies, model organisms and tools, where possible.<br><br>Have you included the information requested as detailed in our Minimum Standards Reporting Checklist?	Yes
**Availability of data and materials**<br><br>All datasets and code on which the conclusions of the paper rely must be either included in your submission or deposited in publicly available repositories (where available and ethically	Yes

appropriate), referencing such data using a unique identifier in the references and in the "Availability of Data and Materials" section of your manuscript.<br><br>Have you have met the above requirement as detailed in our Minimum Standards Reporting Checklist?	

Powered by Editorial Manager® and ProduXion Manager® from Aries Systems Corporation

Click here to download Manuscript bio-TDimpute_zhouxiang_12-14.docx

Imputing missing RNA-seq data from DNA methylation by using transfer learning based neural network

Xiang Zhou[1], Hua Chai[1], Huiying Zhao[2], Ching-Hsing Luo[1]*, and Yuedong Yang[1,3]*

[1]School of Data and Computer Science, Sun Yat-sen University, Guangzhou, China, [2]Sun Yat-sen Memorial Hospital, Sun Yat-sen University, Guangzhou, China, [3]Key Laboratory of Machine Intelligence and Advanced Computing (Sun Yat-sen University), Ministry of Education, China

* yangyd25@mail.sysu.edu.cn; luojinx5@mail.sysu.edu.cn

Abstract

As an epigenetic modification, DNA methylation plays an important role in regulating gene expression. Integrative analysis of DNA methylation and gene expression can capture the associations of the two omics, and thus provides a comprehensive view of the molecular basis underlying cancers. However, it is common that one type of omics data is missing due to various limitations in experiments, preventing downstream analyses that need complete dataset. Imputations from one type of omics data to another is becoming important, but current methods mainly focus on single cancer dataset with limited sample size, and thus are limited by their ability to capture information from large pan-cancer dataset. Here, we present a novel transfer learning-based neural network to impute missing gene expression data from DNA methylation data, namely TDimpute. In the method, the pan-cancer dataset from TCGA was utilized to train a general model for all cancers, which was then fine-tuned on the specific cancer dataset for each cancer. By testing on 16 cancer datasets, we found that our method significantly outperforms other state-of-the-art methods in terms of imputation accuracy (7%-11% increase with different missing rates). The imputed gene expression was also validated to be useful for all downstream analyses, including the identification of both DNA methylation- driving and prognosis-related genes, clustering analysis, and survival analysis. Our method was further proved based on the Wilms tumor dataset from TARGET cancer project.

Introduction

Recent development of molecular biology and high-throughput technologies facilitates the simultaneous measurement of various biological omics data such as genomics, transcriptomics, epigenetics, proteomics, and metabolomics for a single patient. Compared with single-omics analysis, integrative analysis of multi-omics data provides comprehensive insights of cancer occurrence and progression, and thus strengthens our ability to predict cancer prognosis and to discover various levels of biomarker. However, due to technical limitations of experimental settings or high costs for acquiring the omics data, most samples aren't measured with all types of omics data, and lack one part of omics types (called "block missing"). This problem is prevalent in publicly available multi-omics dataset, such as The Cancer Genome Atlas (TCGA). Since gene expression affects clinical outcome and phenotype more directly than molecular features at DNA level (e.g. methylation and genetic variants) [1], we focused on the gene expression data imputation from DNA methylation data.

When the data is missing at random in single omics data, many methods have been proposed for imputing the missing values by using correlation structure among matrix entries, such as singular value decomposition imputation (SVD), k-nearest neighbor (KNN) [2]. However, these traditional methods may not be suitable for the cases lacking a whole set of features. In order to address this issue, several methods have been specifically designed. Voillet et al. used multiple hot-deck imputation approach to impute missing rows in multi-omics dataset

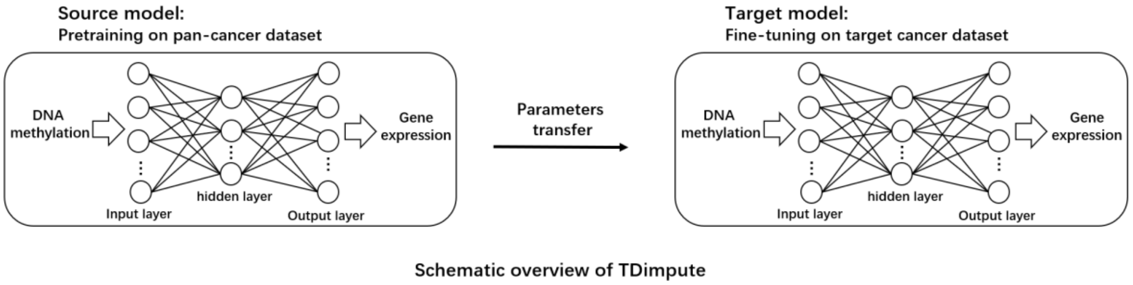

Fig 1. The architecture of transfer learning based neural network (TDimpute) for imputing missing gene expression values in multi-omics dataset. The neural network: DNA methylation data are transformed into gene expression data and the root mean squared error (RMSE) between the actual output and desired output is minimized. Transfer learning: pan-cancer dataset is used to train the general imputation model for pan cancers, which is specifically tuned for each type of cancer.

for multiple factor analysis [3]. To improve the reliability of gene network inference, Imbert et al. used multiple hot-deck imputation method to process RNA-seq data with missing rows, where they measured the similarities to cases in a standard database, and fixed the missing values according to the case with the highest similarity [4]. Obviously, this way to use only the most similar case (neighbor) might be unstable due to random fluctuations in its neighbors. Recently, Dong *et al*. proposed a k-nearest neighbor weighted method (named as TOBMI by the authors) to impute mRNA-missing samples through evaluating the sample similarity by DNA methylation data [5]. However, TOBMI suffers from poor scalability for dataset with large sample size and high dimensionality, and the accuracy is still limited since the size of specific cancer dataset is relatively small. More importantly, it cannot capture information from other related cancer datasets. Based on the regulations from genetic variants and DNA methylation to gene expression, two studies [6] [7] used the least absolute shrinkage and selection operator (LASSO) penalized regression to predict gene expression using genetic variants and DNA methylation, respectively.

In recent years, deep neural network has demonstrated its superiority on modeling complex nonlinear relationships and enjoys scalability and flexibility. One or multiple hidden layers and nonlinear activation function are employed to capture the nonlinear patterns between input and output data. For the gene expression imputation or prediction, many deep learning models have been proposed. Chen *et al*. built a multilayer feedforward neural network to predict the expression of target genes from the expression of ~1000 landmark genes [8]. With the ability to recover partially corrupted input data, denoising autoencoder (DAE) was used to impute missing values in single-cell RNA-seq data [9, 10]. Xie *et al.* constructed a similar deep model to infer gene expression from genotypes of genetic variants [11]. Based on convolutional neural network, Zeng *et al.* used promoter sequences and enhancer-promoter correlations to predict gene expression [12].

One obstacle for the application of these deep learning model to multi-omics dataset is the high dimensionality (>20,000 features) in omics data while a small sample size. Even the TCGA has only hundreds of samples for each cancer type. Thus, it is hard to train an accurate model with millions of parameters in deep learning architecture. In such scenarios, transfer learning is usually considered as a promising method, where parameters trained for a task with large amount of data are reused as the initialization parameters for a similar task with limited data [13]. The transfer learning has been widely used in the computer vision including object detection [14], image segmentation [15].

For the omics data analysis of cancers, the transfer learning strategy has been applied to different tasks. Li *et al.* built a pan-cancer Cox model for the prediction of survival time, where eight cancer types were combined to assist the training of target cancer dataset [13]. Yousefi *et al.* used transfer learning approach to predict the clinical outcomes utilizing samples from uterine corpus endometrial carcinoma and ovarian serous carcinoma to augment target breast cancer dataset to improve the prediction of clinical outcomes [16]. Hajiramezanali *et al.* learned information from the Head and Neck Squamous Cell Carcinoma cancer to subtype lung cancer [17]. Based on the assumption that different types of cancer may share common mechanisms [18, 19], transfer learning is becoming a useful approach for the prediction of missing data by learning from the data of different cancer types.

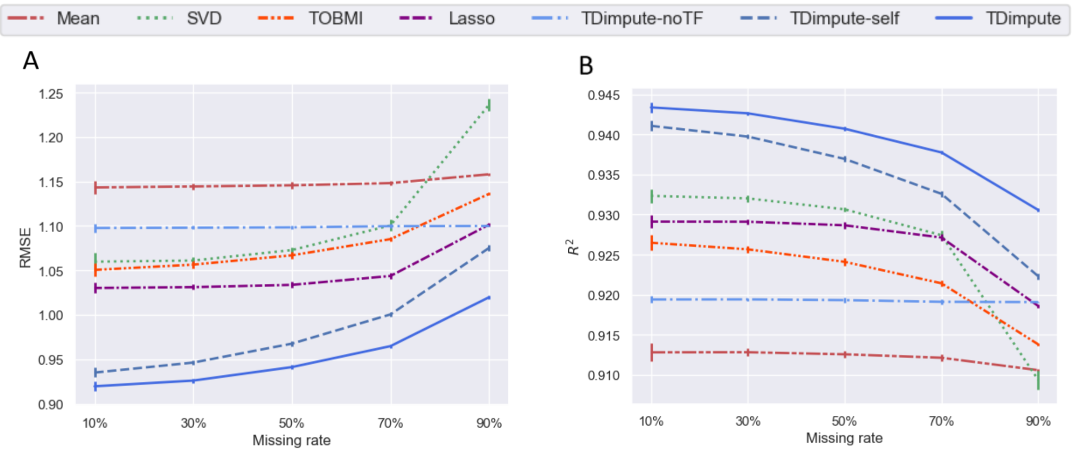

Fig 2. Imputation accuracy of each imputation method. Results were averaged across 16 imputed cancer datasets. (**A**) RMSE values of each method. (**B**) The squared Pearson correlation coefficient (R^2) between each sample of the imputed data and the original full data. TDimpute-self indicates the TDimpute trained and predicted on the target cancer dataset. TDimpute-noTF indicates the TDimpute trained on the pan-cancer dataset (excluding the target cancer) and predicted on the target cancer dataset. The error bar shows the standard deviation.

In this study, we propose a transfer learning based neural network for imputing gene expression from DNA methylation data, namely TDimpute. Specifically, we first train a neural network on the pan-cancer dataset to build a general imputation model for all cancers, which is then transferred to target cancer types (see Fig 1 for a schematic overview). To the best of our knowledge, this is the first time to employ the transfer learning for the imputation of gene expression from methylation. The method was tested to recover gene expressions for 16 cancer types at five different missing rates, and achieved better performances than other methods by measurement of the root mean square errors (RMSE) and Pearson correlation coefficients to actual values. We further evaluated the imputed gene expressions for the identification of methylation-driving genes, prognosis-related genes, clustering analysis, and survival analysis. The results show that our method consistently provides the best performances. These results confirm that TDimpute succeeds in transferring related information from pan-cancer data to target cancer data.

Results

Comparisons on the imputation accuracy

We evaluated the imputation accuracy of different imputation methods by the average root mean square errors (RMSE) and the squared Pearson correlation coefficient R^2 across 16 cancer datasets over different missing rates. The missing rate means the fraction of samples whose gene expression data are removed and evaluates the universal performance of imputation methods with different amount of training samples (especially on small datasets). The samples with missing gene expression are set as testing dataset and the remaining samples are set as training dataset (see Methods section for details of hyper-parameters determination). We compare our method with the Mean imputation method, Lasso [7], TOBMI [5], and SVD imputation method [2]. The default or suggested parameters were used for these methods.

As shown in Fig 2A, Lasso achieves similar but consistently lower RMSE than TOBMI, which indicates that penalized regression has better prediction of the regulation between methylation and gene expression. The performance of SVD is worse, demonstrating a slow increase of RMSE from 1.06 to 1.10 when missing rates change from 10% to 70%, but then a sharp increase to 1.24 that is even higher than the result by the Mean method. Overall, the Mean method has the worst performance, which is consistent with the trend in the original paper [5]. By comparison, TDimpute-self (indicates the TDimpute trained and predicted on the target cancer dataset) without using transfer learning yields 2%-9% lower RMSE than Lasso at different missing rates. TDimpute-noTF, as a general model, is trained on the pan-cancer dataset (excluding the target cancer). The model doesn't use information from the target cancer and thus shows a constant performance. It doesn't perform well but better than the Mean method. The performance is even better than SVD and TOBMI when the missing rate is above 70%. TDimpute, a further transfer learning of the target cancer from TDimpute-noTF, decreases the RMSE by 7%-16%

over TDimpute-noTF. The RMSE by TDimpute is also 2%-5% lower than TDimpute-self with a bigger difference at a higher missing rate. These results confirm the power of our TDimpute method in transferring knowledge from the other cancer types to improve the imputation performance. We also noted SVD, Lasso, and TOBMI have close to constant RMSE values for missing rates 70% and 10%, indicating that 3 times increase of sample sizes don't contribute much to increase the imputation accuracy. Instead, deep learning methods, TDimpute and TDimpute-self, decrease the RMSE by 5% and 7%, respectively, indicating the ability of further improvement with an increase of sample size in future.

When measured by the squared correlation (R^2) between the imputed and actual values by each sample (Fig 2B), TDimpute is consistently the best, followed by the TDimput-self. Differently, SVD ranks the 3rd except at a missing rate of 90%, where SVD has the lowest R^2 of 0.909. The Mean imputation keeps the lowest performance. Hereafter, we will focus on the comparison with SVD, Lasso, and TOBMI methods.

Impact on the methylation-expression correlations and the identification of methylation-driving genes

For multi-omics dataset, proper imputation method should preserve the correlation structures between different types of omics. Since the most correlated CpG-gene pairs play the most important roles, we only compare the impact of imputation methods by the average R^2 of top 100 CpG-gene pairs from full datasets. As shown in Fig 3, Lasso and TOBMI display a dramatic decrease of R^2 (from 0.78 to 0.64, from 0.79 to 0.63, respectively), with the increase of missing rates. SVD method has a small decrease of R^2 from 0.80 to 0.79 when missing rates increase from 10% to 50%, but a large drop to 0.72 at a missing rate of 90%. In contrast, both TDimpute and TDimpute-self have R^2 values fluctuating around the ideal values (R^2=0.8) when missing rates are less than 70%. At a missing rate of 90%, TDimpute-self by using single dataset has a drop of R^2 to 0.75, while TDimpute doesn't show any decrease. This is as expected because transfer learning has been widely proven to solve the problem of small sample sizes.

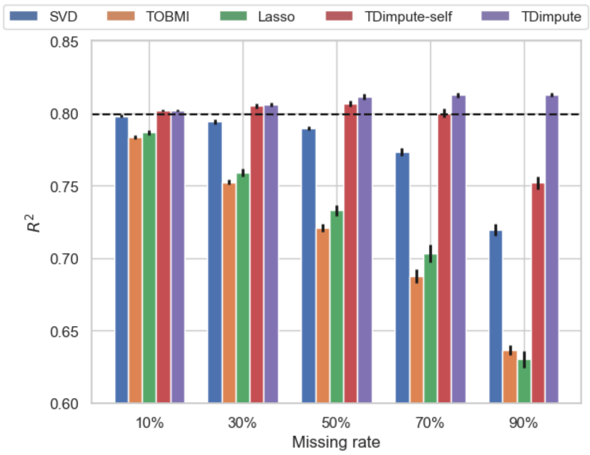

Fig 3. The average correlations R^2 of top 100 CpG-gene pairs over 16 cancer datasets by four imputed methods. Dashed black line is the correlations from the actual dataset. The error bar shows the standard error of the mean.

Table 1. The average PR-AUC over 16 cancers for recovering methylation-driving genes according to the imputed relative to the actual gene expression data. Average performance across 16 imputed cancer datasets are reported. Best results are highlighted in bold face. The * indicates statistical significance (paired T-test, p-value < 0.05) between TDimpute and other methods.

Missing rate	SVD	TOBMI	Lasso	TDimpute-self	TDimpute
10%	0.988*	0.988*	0.985*	0.992*	**0.993**
30%	0.954*	0.956*	0.942*	0.969*	**0.974**
50%	0.900*	0.898*	0.874*	0.932*	**0.943**
70%	0.818*	0.807*	0.794*	0.867*	**0.892**
90%	0.665*	0.651*	0.676*	0.712*	**0.788**

We further investigate whether the preservation of correlations can obtain better performance in the identification of methylation-driving genes. The performance is evaluated by PR-AUC and the overlap of top 100 methylation-driving gene from imputed and full datasets. A higher value means stronger concordance with the gene list identified from actual dataset. Tables 1 and S3.1 show that our proposed TDimpute method has the highest PR-AUC values and overlap with true methylation-driving genes among the four methods across different missing rates. TDimpute-self ranks the second in selecting methylation-driving genes, followed by SVD, TOBMI, and Lasso. Compared with SVD, TDimpute achieves 0.5%-19% improvement for PR-AUC, and 3%-103% improvement for overlapped genes. The improvement is especially pronounced at high missing rates.

Table 2. The average PR-AUC for recovering prognosis-related genes according to the imputed relative to the actual gene expression data. The * indicates statistical significance (paired T-test, p-value < 0.05) between TDimpute and other methods.

Missing rate	SVD	TOBMI	Lasso	TDimpute-self	TDimpute
10%	0.901*	0.912*	0.912*	0.923*	0.927
30%	0.711*	0.747*	0.742*	0.768*	0.784
50%	0.561*	0.595*	0.590*	0.627*	0.652
70%	0.428*	0.454*	0.449*	0.487*	0.523
90%	0.286*	0.287*	0.292*	0.311*	0.376

Table 3. The average enrichment factors of top 100 prognosis-related genes overlapped with the genes collected in the Human Protein Atlas. The * indicates statistical significance (paired T-test, p-value < 0.05) between TDimpute and other methods.

Missing rate	SVD	TOBMI	Lasso	TDimpute-self	TDimpute
10%	5.53*	5.83	5.55	5.71	**5.91**
30%	3.46*	4.08	3.71*	4.22	**4.25**
50%	2.08*	2.74	2.42*	2.94	**3.06**
70%	1.14*	1.60*	1.33*	1.87	**2.03**
90%	0.56*	0.47*	0.49*	0.89*	**1.14**

Impact on the identification of prognosis-related genes

We investigated the recovery power of different imputation methods on the identification of significantly prognosis-related genes. To evaluate the selected genes, we compared the genes identified from the imputed to those from the actual data. Consistent with the performance in the imputation accuracy, Tables 2 and S5.1 show that TDimpute method achieves 2%-28% higher PR-AUC values, and 4%-54% more number of overlapped genes than those by the TOBMI method. Lasso achieves lower values than TOBMI, except at a missing rate of 90%, where Lasso performs slightly better than TOBMI.

We also investigate the enrichment of the top 100 genes (ranked by p-values) overlapped with the prognosis-related gene list downloaded from The Human Protein Atlas [20] relative to the random. Table 3 demonstrates that TDimpute achieves the largest enrichment factors (see Methods section for definition), indicating its ability to identify the really validated prognosis-related genes.

Impact on the performance of clustering analysis and survival analysis

We also evaluate the effects of different imputation methods on clustering analysis and survival analysis. By input of top 100 prognosis-related genes, K-means algorithm is used to divided the samples into two clusters. The adjusted rand index (ARI) for evaluating the concordance between the clusters from the imputed and actual data is shown in Fig 4A. For all methods, accuracy decreases with increasing missing rates, which is consistent with the previous study [10]. As expected, TDimpute achieves the highest clustering concordance among the five imputation methods consistently under different missing rates.

A further survival analysis (Fig 4B) shows that TDimpute achieves the best C-index, followed by TDimpute-self, SVD method, TOBMI method, and Lasso method. Despite showing a worse performance in the imputation accuracy, SVD performs better than TOBMI in this evaluation metric. In addition, the C-index of TDimpute, TDimpute-self, and SVD are relatively robust to the missing rates compared to TOBMI that showed a 9% decrease in C-index with 90% of samples missing gene expression values.

For all the mentioned experiments, the results per cancer dataset are detailed in S1-S5 Figs, and S2-S6 Tables.

Independent test on Wilms tumor from the TARGET dataset

Our method was further tested on TARGET dataset from an independent source. We constructed a model by fine-tuning the TCGA pan-cancer model using randomly selected 59 samples (50% of the dataset) with the same hyper-parameters optimized in the TCGA experiments. As expected, TDimpute achieves the lowest RMSE of 0.955 (TDimpute-self: 0.98; SVD: 1.064; Lasso: 1.006; TOBMI: 1.018). K-means method is used to cluster the 118 samples after imputation, and two resulted clusters are

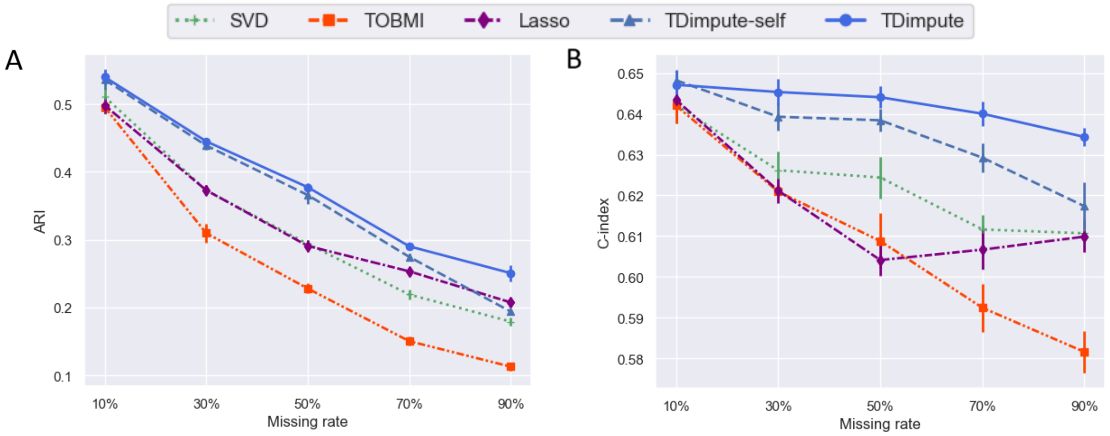

Fig 4. **(A)** The average adjusted rand index (ARI) of the clusters from the imputed and actual data, and **(B)** the average C-index by survival analyses based on imputed data over 16 cancers. The error bar shows the standard error of the mean.

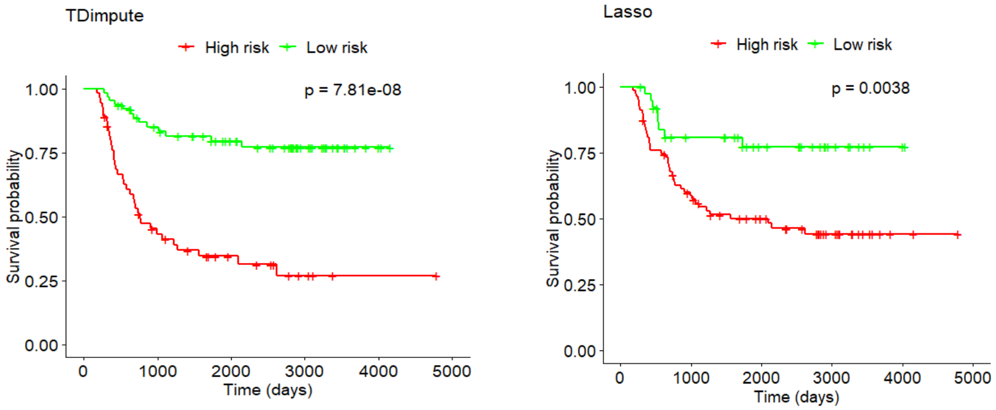

Fig 5. Kaplan-Meier plot for the two clusters obtained from the Wilms tumor dataset imputed by TDimpute and Lasso, respectively.

used to plot the survival curve. The log-rank test was used to evaluate the difference in prognosis of each cluster. As shown in Fig 5, TDimpute achieves more significant difference between the two clusters, where the p-value is decreased from 0.0038 (Lasso) to 7.81e-08 (TDimpute). TDimpute-self and TOBMI achieve the second and third highest significance with p-value of 4.53e-07 and 8.64e-06, respectively, while the p-value of SVD is much higher (0.0299). The survival analysis on the imputed dataset by TDimpute are also enhanced with the largest C-index of 0.592, compared to TDimpute-self, SVD, TOBMI, Lasso with C-index of 0.591, 0.523, 0.558, and 0.501, respectively.

Discussion

In this paper, TDimpute perform missing gene expression imputation by building a highly nonlinear mapping from DNA methylation data to gene expression data. Due to the limited size of cancer datasets in TCGA, we use transfer learning to capture the commonalties in pan-cancer dataset for parameter pre-training. We compare TDimpute with/without transfer learning, Lasso, SVD and TOBMI method in imputation accuracy of RMSE and correlation R^2, methylation-expression correlations. Since the main task of imputation is to recover biologically meaningful gene expression data for downstream analysis, we also evaluate the imputation performance for the identification of methylation-driving gene, prognosis-related genes, clustering analysis and survival prediction. It is worthy to note that although only methylation and gene expression data are illustrated in this paper, our method is capable of incorporating other omics data by following a similar framework.

Experimental results on 16 cancer datasets confirm that our TDimpute method without transfer learning outperforms Lasso, SVD, and TOBMI method in different evaluation metrics. Based on transfer learning, our TDimpute method can further improve the performance especially at large missing rate. In addition, the ranking of Lasso, SVD, and TOBMI method by imputation accuracy (RMSE and correlation R^2) are not strictly

correlated with their performance in preservation of methylation-expression correlation, clustering analysis and survival prediction, but our TDimpute method provide approximately consistent performance in downstream analysis.

Besides the good performance of imputation accuracy and downstream analysis, another main benefit of our proposed methods is the computational efficiency and convenience. Based on GPU acceleration, our TDimpute method is capable of processing large-scale pan-cancer dataset including tens of thousands of samples and hundreds of thousands of features, while Lasso, TOBMI, and SVD suffer poor scalability due to the computational complexity of distance matrix computation and singular value decomposition operations. Based on pre-trained model, transfer learning framework can also accelerate the training process on the target dataset.

In previous study of genome-wide association analysis (GWAS) without directly measured gene expression [6, 21], gene expression were imputed from genetic data to perform transcriptome-wide association analysis (TWAS) that can reduce multiple-testing burden and identify associated genes. In future, the predicted gene expression from the methylation data can also be integrated in epigenome-wide association studies (EWAS) [22].

Future work can focus on reducing the amount of model parameters and integrating more related training samples. Since we only use the correlation between omics for imputation, one possible direction is to leverage prior knowledge of gene-gene interaction network. The known relationships between variables/genes has demonstrated its ability to significantly reduce the model parameters by enforcing sparsity on the connections of neural network [23]. The performance of this approach is dependent on the quality of the gene-gene networks, and more investigation need to be done in this direction.

Methods

Public datasets and preprocessing

TCGA: We obtained the data for 33 cancer types from The Cancer Genome Atlas (TCGA) using the R package TCGA-assembler [24], including RNA-seq gene expression data (UNC IlluminaHiSeq_RNASeqV2_RSEM), DNA methylation data (JHU-USC HumanMethylation450), and clinical information with follow-up. Originally, 20531 genes and 485577 methylation sites were collected. We excluded genes with zero values in the RNA-seq data across all samples. The remained 19027 genes were converted by the $\log_2(\mathrm{G}+1)$, where G is the raw gene expression value. For DNA methylation data, we excluded methylation sites with "NA" values, and 269023 methylation sites remain. By further removing sites with small variances (< 0.05) over all samples, 27717 CpG sites were kept. Here, for evaluating all imputing methods we kept only samples having both RNA-seq and DNA methylation data. Finally, the dataset contains 8856 samples with expression data for genes and methylation values for 33 cancers, namely pan-cancer dataset.

To keep enough sample size for downstream analysis, cancer types containing > 200 samples with complete DNA methylation, gene expression, and clinical data were selected. We used different missing rates to evaluate the performance of imputation methods under different number of training samples (especially on small datasets). After filtering, we obtained 16 cancer types for test: Breast adenocarcinoma (BRCA), Thyroid carcinoma (THCA), Brain lower grade glioma (LGG), head and neck squamous cesll carcinoma (HNSC), Prostate adenocarcinoma (PRAD), Lung adenocarcinoma (LUAD), Skin cutaneous melanoma (SKCM), Bladder urothelial carcinoma (BLCA), Liver hepatocellular carcinoma (LIHC), Lung squamous cell carcinoma (LUSC), Skin cutaneous melanoma (STAD), Kidney renal clear cell carcinoma (KIRC), Cervical squamous cell carcinoma and endocervical adenocarcinoma (CESC), Kidney renal papillary cell carcinoma (KIRP), Colon carcinoma (COAD), and Sarcoma (SARC). The cancer types and their sample sizes are detailed in Table S1.

TARGET: For an independent test, we constructed one dataset developed from another cancer project: the Therapeutically Applicable Research To Generate Effective Treatments (TARGET) project. We chose the Wilms tumor (the most common type of childhood kidney cancer) as it has the smallest sample size. Here, the DNA methylation data was downloaded from the TARGET Data Matrix [25], and its corresponding RNA-seq data (RSEM estimated read counts) was downloaded from UCSC Xena [26].

Finally, we obtained 118 samples with complete gene expression data, methylation data, and clinical data, which were randomly split into training and test datasets with proportion of 1:1. Quantile normalization [27] was performed to remove technical variabilities between TCGA and TARGET datasets: specifically, the data from TCGA were used as reference to normalize the TARGET data into the same distribution.

The architecture of our imputation method

Neural network was employed for imputing missing gene expression values from the DNA methylation. To expand the sample size for training the model, we leverage the pan-cancer dataset to generate a model for all cancer types. Then, the model is fine-tuned respectively on each cancer type to obtain specific models.

Neural network architecture. As shown in Fig 1, the neural network includes input layer, output layer, and one or multiple hidden layers. The nodes between layers are fully connected. Here, we use x^0 to represent the input of network, and the output vector x^l at lth layer can be formulated as

$$\boldsymbol{x}^l = f(\boldsymbol{W}^l \boldsymbol{x}^{l-1} + \boldsymbol{b}^l) \quad (1)$$

where $\boldsymbol{x}^{l-1}$ denotes the output of previous layer $l-1$, $f(\cdot)$ is the activation function such as the sigmoid and Relu functions, and $\boldsymbol{W}$ and $\boldsymbol{b}$ are weight matrix and bias vector, respectively. $\boldsymbol{W}$ and $\boldsymbol{b}$ are parameters that need to be learned.

The loss function for training is the root mean squared error (RMSE):

$$L(y, \mathrm{y}^0) = \sqrt{\frac{1}{N}\sum_{i=1}^{N}(y_i - \mathrm{y}_\mathrm{i}^0)^2} \quad (2)$$

where y_i^0 and y_i are the experimentally measured and predicted expression value for gene i, and N is the dimension of output vector (i.e., the number of genes). The network can be considered as a highly nonlinear regression function that maps DNA methylation data (input) to gene expression data (output).

Transfer learning-based models. To train the prediction model for one target cancer in the TCGA, the datasets of other cancer types are combined to generate a multi-cancer model that is then fine-tuned by the target cancer data (Fig 1). The data of the target cancer was excluded to train the multi-cancer model as we need to remove different portions of the data for the target cancer to evaluate our imputation model.

Missing data simulations and hyper-parameters setting

To simulate the missing values in omics, we randomly selected increasing fractions (10%, 30%, 50%, 70%, 90%) of samples in the full dataset and remove their gene expression data. The samples with missing gene expression are set as testing dataset and the remaining samples with complete omics are set as training dataset. Different missing rates were designed to evaluate all imputation methods with different number of training samples (especially on small datasets). At each level of missing rate, we repeat this procedure 5 times to obtain a robust evaluation of each method and the averaged results are reported in all the following experiments. The statistical significance is assessed by paired T-test. The original full dataset is referred as a gold standard for our comparisons. For TDimpute and SVD method, gene expression data are scaled to the range of [0, 1].

For our neural network, we selected BRCA dataset to optimize all hyper-parameters by RMSE through 5-fold cross validation. Here, we selected the hyper-parameters on the BRCA dataset and then applied the optimal hyper-parameters to all cancer types. Based on the pan-caner dataset (excluding BRCA dataset), we first tune the pretrained model to determine the architecture of neural network, i.e., the number of hidden layers and the hidden layer size, and the epochs to stop training. Details on the performance analysis for each hyper-parameter are provided in tables S7 and Fig S6 in the supplementary material. For the fine-tune process on the BRCA dataset, the only hyper-parameter we need to choose is the training epoch since the network architecture is fixed by the pretrained model. Some transfer learning strategies freeze weights for certain layers and fine-tune other layers. Here, we don’t freeze any layer, as this demonstrated better performance. In Fig S7, we show the convergence process of different missing rates on the validation dataset of BRCA.

Finally, we selected the following hyper-parameters for pan-cancer model: 1 hidden layer (selected from 1 and 3) including 4000 nodes (from

500, 1000, 2000, 4000, and 5000), Sigmoid activation function (from Tanh, Relu, and Sigmoid), epochs of 300 (from 50, 100, 150, 300, and 500), and batch size of 128. For the fine-tuning stage, 150 epochs (from 50, 100, 150, 300, and 500) were used and the batch size was set as 16 because of small training dataset under large missing rate. The model was trained using Adam optimizer with default parameters (learning rate set as 0.0001) [28]. Dropout wasn't used as it decreased the performance [29]. The method was implemented with TensorFlow [30].

All the codes and pretrained pan-cancer models are available on Github: https://github.com/sysu-yanglab/TDimpute.

Preservation of methylation-expression correlations and methylation-driving genes

Here, we use the squared Pearson correlation coefficient R^2 to evaluate the effect of imputation method on the correlations between DNA methylation and gene expression. Since one gene might be associated with multiple CpG sites, we only considered the CpG-gene pair with the strongest correlation in this paper. Based on the methylation-expression regulation, many studies have been conducted to identify cancer-related DNA methylation-driving (hyper and hypo methylated) genes [31]. Hence, we also evaluate the effect of imputation methods on the identification of methylation-driving genes. We define the methylation-driving genes (i.e., significantly correlated CpG-gene pairs) with the $R^2 \geq 0.5$ and FDR-$q \leq 0.05$. The pairs with R^2 greater than a threshold are considered to be correlated, according to which we can obtain the area under precision-recall curve (PR-AUC). We also computed the overlap between the top 100 ranked genes identified from imputed datasets and the original full datasets.

Preservation of prognosis-related genes

A common task in the analysis of gene expression data is the identification of prognostic genes. In order to evaluate the effect of different imputation method on the identification of potentially prognosis-related gene, we build univariate Cox proportional hazard regression models to select statistically significant genes correlated with overall survivals. With the Cox model, each gene is assigned a p-value describing the significance of the relation between the gene and a target cancer. The prognosis-related genes are identified with p-value ≤ 0.05. We rank the genes by their p-values, and evaluate the consistency between the gene lists from imputed datasets and the original full datasets using PR-AUC and the overlapped top 100 ranked genes.

To validate our gene rankings with independent information, we download the list of prognosis-related genes from The Human Protein Atlas (THPA) [20], and compare the enrichment factors of the top 100 ranked genes in the list from THPA. The enrichment factor is calculated with $EF = (N_{\mathrm{True}}/N_{\mathrm{selected}})/(N_{\mathrm{Active}}/N_{\mathrm{Total}})$, where N_{True} is the number of true positives, N_{selected} is the number of top k selected genes, N_{Active} and N_{Total} are the number of prognosis-related genes and total number of genes in THPA, respectively.

Impact on clustering analysis and survival analysis

We evaluated the relation of genes to cancer survivals by p-values output from the univariate Cox model. By using the top 100 genes, their expression values were used to divided samples into 2 clusters by the K-means. The clustering performance was assessed by adjusted rand index (ARI), which is a measure of agreement between the predicted cluster labels (on imputed dataset) and the true cluster labels (on original full dataset). We further made survival prediction with significantly related genes ($p \leq 0.05$) by using the ridge regression regularized Cox model. Here, the glmnet package [32] in R was used for model construction, which is suitable for fitting regression model with high-dimensional data. The performance of the Cox model was assessed by the Harrell's concordance index (C-index) that measures the concordance between predicted survival risks and actual survival times. We used 5-fold cross validation (CV) to evaluate the performance.

Availability of source code and pretrained model

All the codes and pretrained pan-cancer models are available on Github: https://github.com/sysu-yanglab/TDimpute.

Acknowledgements

We'd like to acknowledge TCGA to make the data publicly available.

Competing interests

The authors declare that they have no competing interests.

Funding

This work has been supported by the National Key R&D Program of China (2018YFC0910500), National Natural Science Foundation of China (U1611261, 61772566, and 81801132), Guangdong Frontier & Key Tech Innovation Program (2018B010109006, 2019B020228001), Natural Science Foundation of Guangdong, China (2019A1515012207), and Introducing Innovative and Entrepreneurial Teams (2016ZT06D211).

References

1. Wang W, Baladandayuthapani V, Morris JS, Broom BM, Manyam G, Do K-A. iBAG: integrative Bayesian analysis of high-dimensional multiplatform genomics data. Bioinformatics. 2013;29(2):149-159. doi: 10.1093/bioinformatics/bts655.
2. Troyanskaya O, Cantor M, Sherlock G, Brown P, Hastie T, Tibshirani R, et al. Missing value estimation methods for DNA microarrays. Bioinformatics. 2001;17(6):520-525.
3. Voillet V, Besse P, Liaubet L, San Cristobal M, Gonzalez I. Handling missing rows in multi-omics data integration: multiple imputation in multiple factor analysis framework. BMC Bioinformatics. 2016;17(1):402. doi: 10.1186/s12859-016-1273-5 PMID: 27716030.
4. Imbert A, Valsesia A, Le Gall C, Armenise C, Lefebvre G, Gourraud PA, et al. Multiple hot-deck imputation for network inference from RNA sequencing data. Bioinformatics. 2018;34(10):1726-1732. doi: 10.1093/bioinformatics/btx819 PMID: 29280999.
5. Dong X, Lin L, Zhang R, Zhao Y, Christiani DC, Wei Y, et al. TOBMI: Trans-omics block missing data imputation using a k-Nearest Neighbor weighted approach. Bioinformatics. 2018;35(8):1278-1283. doi: 10.1093/bioinformatics/bty796 PMID: 30202885.
6. Hu Y, Li M, Lu Q, Weng H, Wang J, Zekavat SM, et al. A statistical framework for cross-tissue transcriptome-wide association analysis. Nat Genet. 2019;51(3):568-576. doi: 10.1038/s41588-019-0345-7 PMID: 30804563.
7. Zhong H, Kim S, Zhi D, Cui X. Predicting gene expression using DNA methylation in three human populations. PeerJ. 2019;7. doi: 10.7717/peerj.6757.
8. Chen Y, Li Y, Narayan R, Subramanian A, Xie X. Gene expression inference with deep learning. Bioinformatics. 2016;32(12):1832-9. doi: 10.1093/bioinformatics/btw074 PMID: 26873929.
9. Eraslan G, Simon LM, Mircea M, Mueller NS, Theis FJ. Single-cell RNA-seq denoising using a deep count autoencoder. Nat Commun. 2019;10(1):390. doi: 10.1038/s41467-018-07931-2 PMID: 30674886.
10. Tian T, Wan J, Song Q, Wei Z. Clustering single-cell RNA-seq data with a model-based deep learning approach. Nature Machine Intelligence. 2019;1(4):191-198. doi: 10.1038/s42256-019-0037-0.
11. Xie R, Wen J, Quitadamo A, Cheng J, Shi X. A deep auto-encoder model for gene expression prediction. BMC Genomics. 2017;18(Suppl 9):845. doi: 10.1186/s12864-017-4226-0 PMID: 29219072.
12. Zeng W, Wang Y, Jiang R. Integrating distal and proximal information to predict gene expression via a densely connected convolutional neural network. Bioinformatics. 2019. doi: 10.1093/bioinformatics/btz562 PMID: 31318408.
13. Li Y, Wang L, Wang J, Ye J, Reddy CK, editors. Transfer learning for survival analysis via efficient L2, 1-norm regularized Cox regression. 2016 IEEE 16th International Conference on Data Mining (ICDM); 2016: IEEE.
14. Girshick R, Donahue J, Darrell T, Malik J, editors. Rich feature hierarchies for accurate object detection and semantic segmentation. Proceedings of the IEEE conference on computer vision and pattern recognition; 2014.
15. He K, Gkioxari G, Dollár P, Girshick R, editors. Mask r-cnn. Proceedings of the IEEE international conference on computer vision; 2017.
16. Yousefi S, Amrollahi F, Amgad M, Dong C, Lewis JE, Song C, et al. Predicting clinical outcomes from large scale cancer genomic profiles with deep survival models. Sci Rep. 2017;7(1):11707. doi: 10.1038/s41598-017-11817-6 PMID: 28916782.

17. Hajiramezanali E, Dadaneh SZ, Karbalayghareh A, Zhou M, Qian X, editors. Bayesian multi-domain learning for cancer subtype discovery from next-generation sequencing count data. Advances in Neural Information Processing Systems; 2018.
18. Yang X, Gao L, Zhang S. Comparative pan-cancer DNA methylation analysis reveals cancer common and specific patterns. Brief Bioinform. 2017;18(5):761-773. doi: 10.1093/bib/bbw063 PMID: 27436122.
19. Hoadley KA, Yau C, Wolf DM, Cherniack AD, Tamborero D, Ng S, et al. Multiplatform analysis of 12 cancer types reveals molecular classification within and across tissues of origin. Cell. 2014;158(4):929-944. doi: 10.1016/j.cell.2014.06.049 PMID: 25109877.
20. Uhlen M, Zhang C, Lee S, Sjostedt E, Fagerberg L, Bidkhori G, et al. A pathology atlas of the human cancer transcriptome. Science. 2017;357(6352). doi: 10.1126/science.aan2507 PMID: 28818916.
21. Gamazon ER, Wheeler HE, Shah KP, Mozaffari SV, Aquino-Michaels K, Carroll RJ, et al. A gene-based association method for mapping traits using reference transcriptome data. Nature Genetics. 2015;47(9):1091-1098. doi: 10.1038/ng.3367.
22. Xu J, Zhao L, Liu D, Hu S, Song X, Li J, et al. EWAS: epigenome-wide association study software 2.0. Bioinformatics. 2018;34(15):2657-2658. doi: 10.1093/bioinformatics/bty163 PMID: 29566144.
23. Kong Y, Yu T. A graph-embedded deep feedforward network for disease outcome classification and feature selection using gene expression data. Bioinformatics. 2018;34(21):3727-3737. doi: 10.1093/bioinformatics/bty429 PMID: 29850911.
24. Wei L, Jin Z, Yang S, Xu Y, Zhu Y, Ji Y. TCGA-assembler 2: software pipeline for retrieval and processing of TCGA/CPTAC data. Bioinformatics. 2018;34(9):1615-1617. doi: 10.1093/bioinformatics/btx812 PMID: 29272348.
25. https://ocg.cancer.gov/programs/target/data-matrix.
26. Vivian J, Rao AA, Nothaft FA, Ketchum C, Armstrong J, Novak A, et al. Toil enables reproducible, open source, big biomedical data analyses. Nat Biotechnol. 2017;35(4):314-316. doi: 10.1038/nbt.3772 PMID: 28398314.
27. Bolstad B. preprocessCore: A collection of pre-processing functions. R package version 1.48.0. Available from: https://github.com/bmbolstad/preprocessCore.
28. Kingma DP, Ba J. Adam: A Method for Stochastic Optimization. arXiv e-prints [Internet]. 2014 December 01, 2014. Available from: https://ui.adsabs.harvard.edu/abs/2014arXiv1412.6980K.
29. Srivastava N, Hinton G, Krizhevsky A, Sutskever I, Salakhutdinov R. Dropout: a simple way to prevent neural networks from overfitting. The journal of machine learning research. 2014;15(1):1929-1958.
30. Abadi M, Barham P, Chen J, Chen Z, Davis A, Dean J, et al. TensorFlow: a system for large-scale machine learning. Proceedings of the 12th USENIX conference on Operating Systems Design and Implementation; Savannah, GA, USA. 3026899: USENIX Association; 2016. p. 265-283.
31. Champion M, Brennan K, Croonenborghs T, Gentles AJ, Pochet N, Gevaert O. Module Analysis Captures Pancancer Genetically and Epigenetically Deregulated Cancer Driver Genes for Smoking and Antiviral Response. EBioMedicine. 2018;27:156-166. doi: 10.1016/j.ebiom.2017.11.028 PMID: 29331675.
32. Friedman J, Hastie T, Tibshirani R. Regularization paths for generalized linear models via coordinate descent. Journal of statistical software. 2010;33(1):1.

Supplementary Figure 1

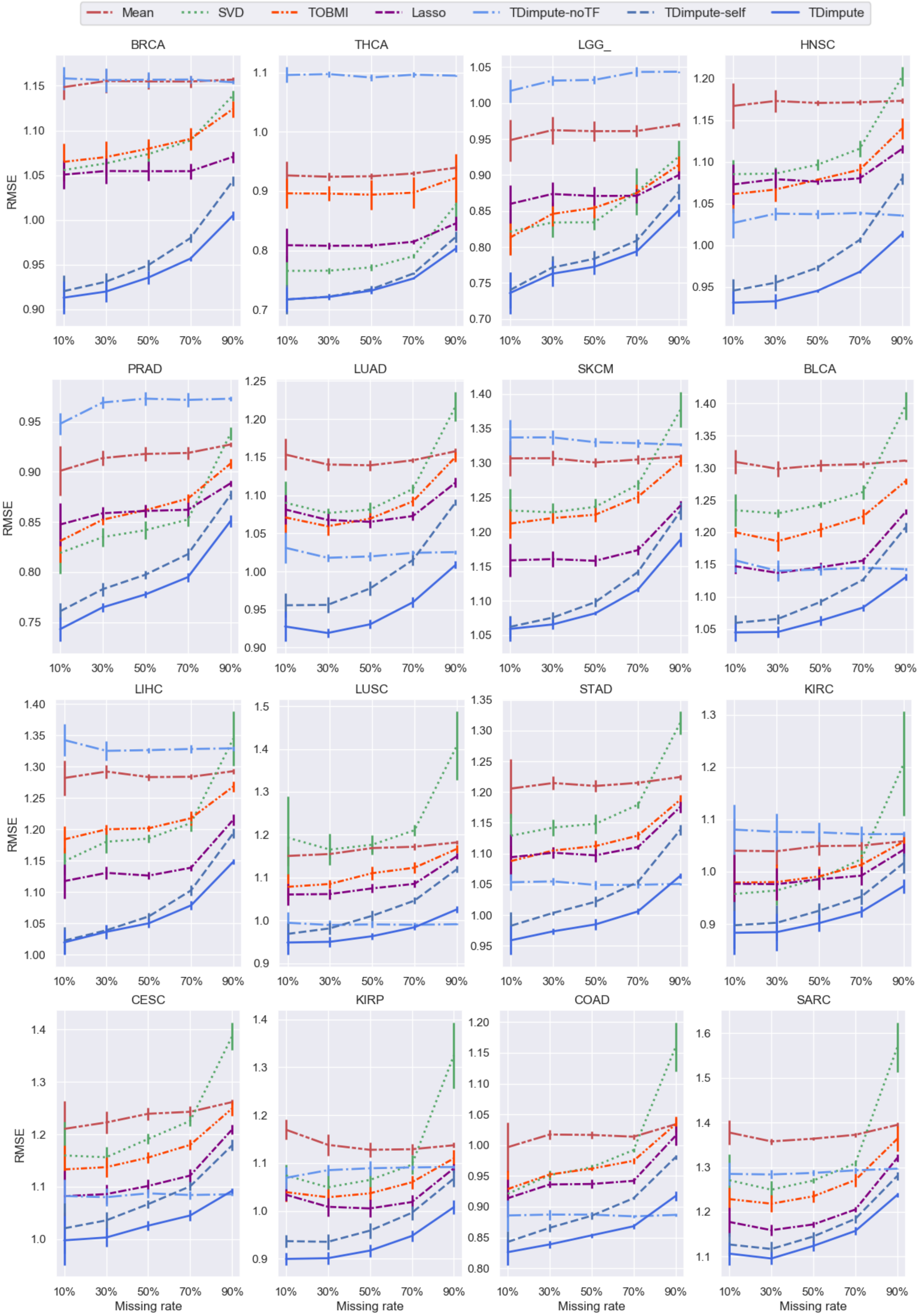

Fig S1. RMSE on 16 imputed cancer datasets with different missing rates. The results were averaged over 5 random replicas. The error bar shows the standard deviation.

Supplementary Figure 2

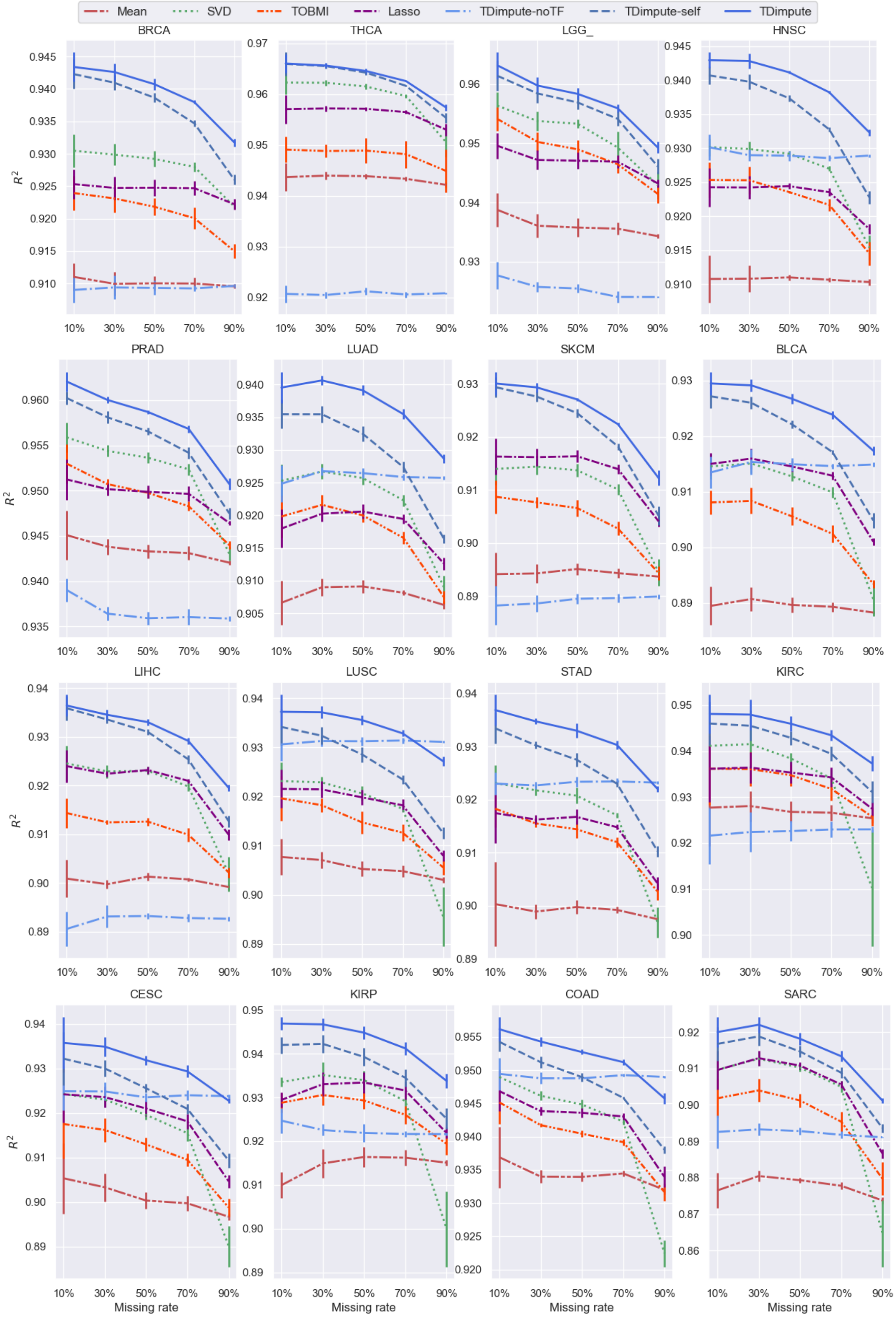

Fig S2. The squared Pearson correlation coefficients R^2 between each sample of the imputed data and the original full data on 16 imputed cancer datasets with different missing rates. The results were averaged over 5 random replicas. The error bar shows the standard deviation.

Supplementary Figure 3

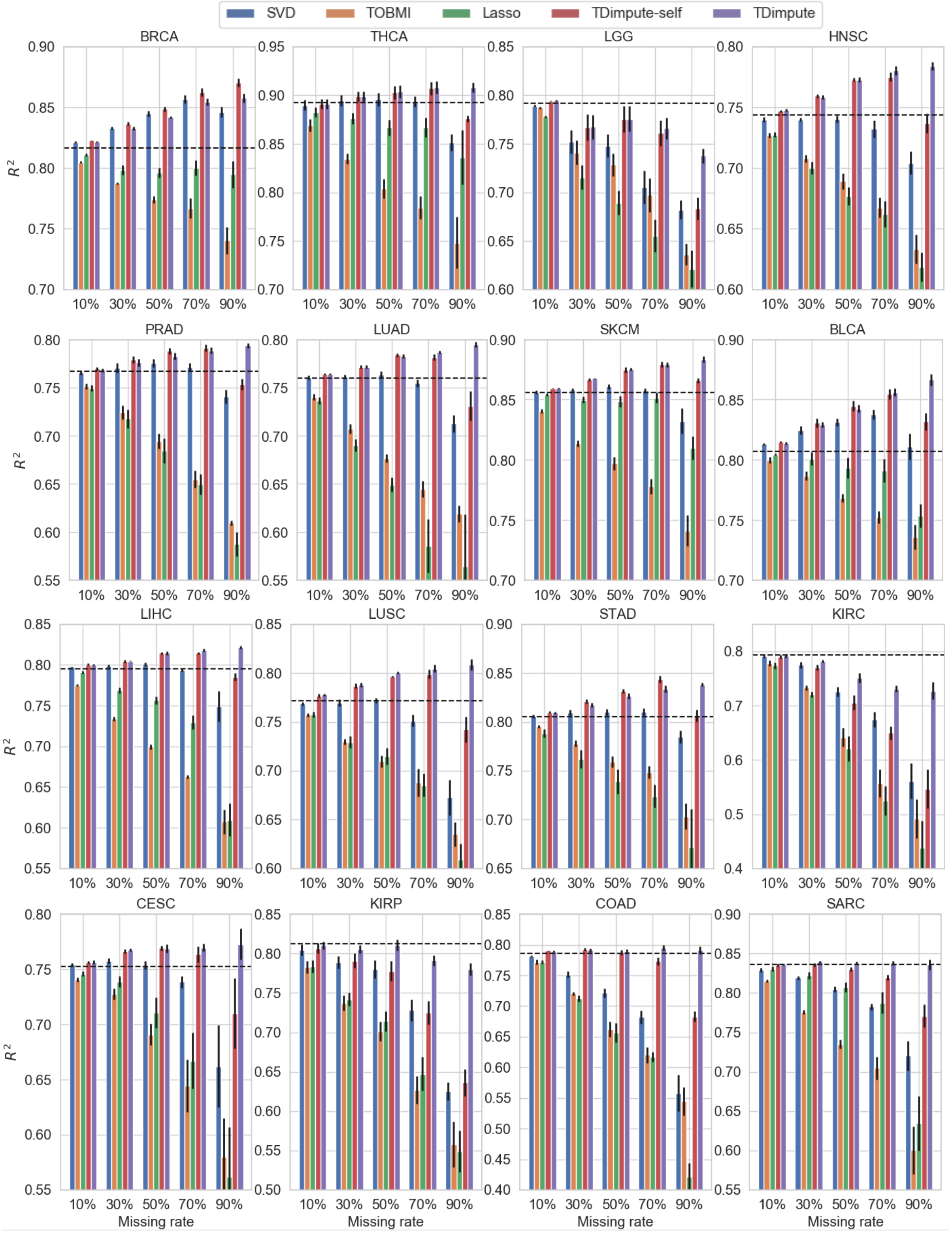

Fig S3. The squared Pearson correlation coefficients R^2 between gene expression and methylation sites on 16 imputed cancer datasets with different missing rates. The results were averaged over 5 random replicas. Dashed black line is drawn as a reference indicating the correlations from the original full dataset. The error bar shows the standard error of the mean.

Supplementary Figure 4

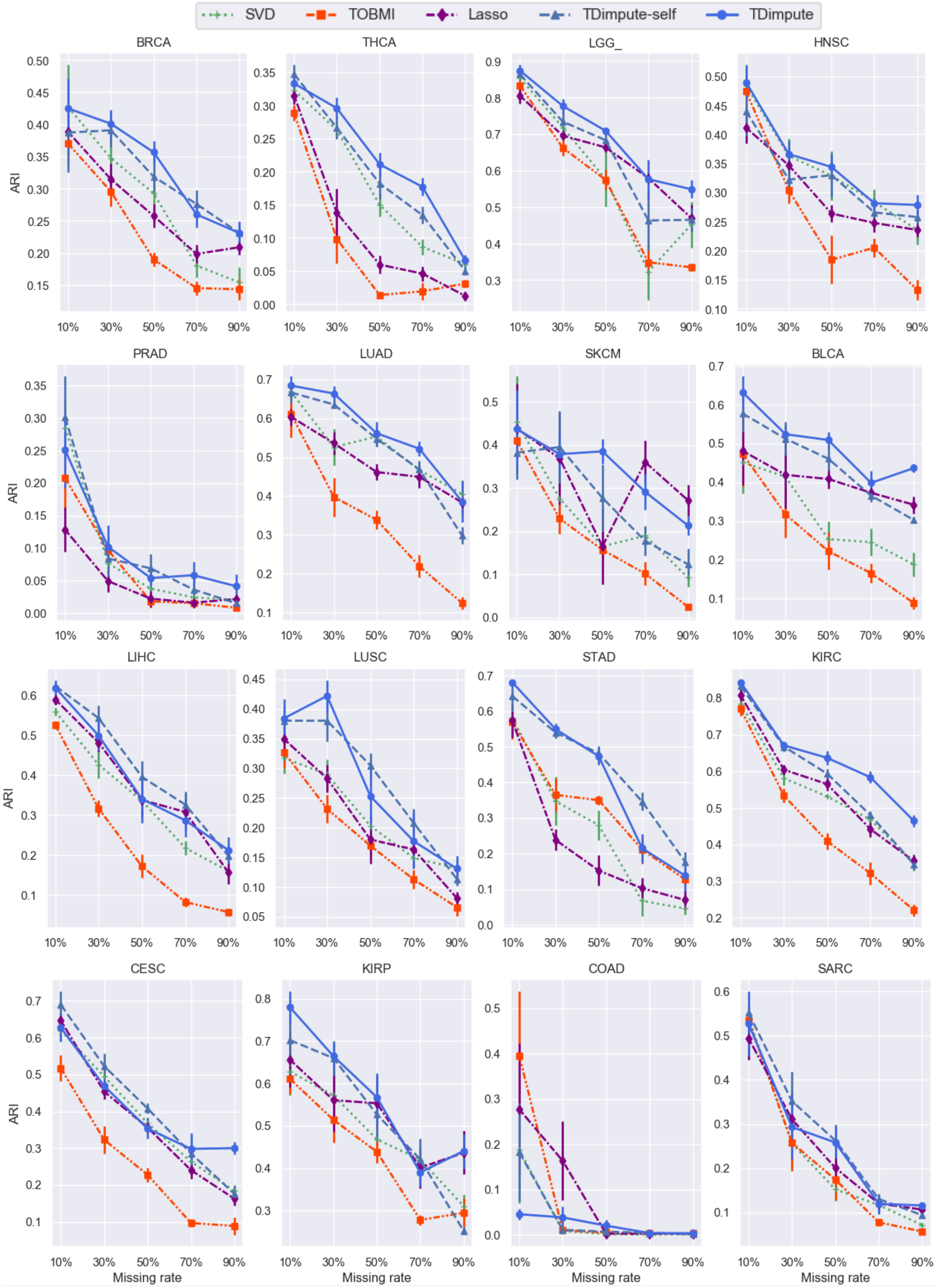

Fig S4. ARI on 16 imputed cancer datasets with different missing rates. The results were averaged over 5 random replicas. The error bar shows the standard error of the mean.

Supplementary Figure 5

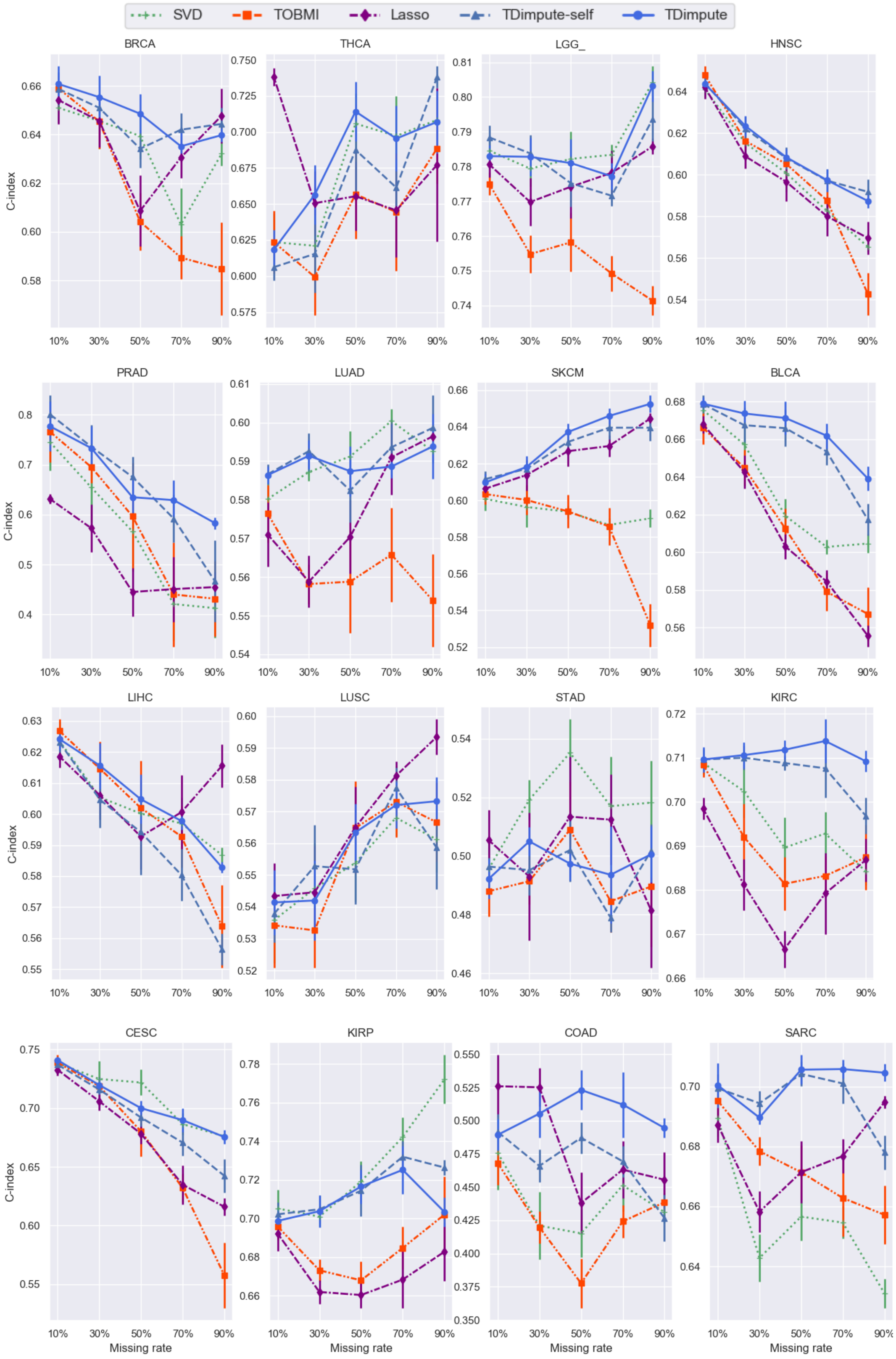

Fig S5. C-index on 16 imputed cancer datasets with different missing rates. The results were averaged over 5 random replicas. The error bar shows the standard error of the mean.

Table S1. The TCGA cancer types and their sample sizes used for test.

**Cancers**	**Dataset size**
BRCA	867
THCA	562
HNSC	541
LGG_	541
PRAD	532
LUAD	477
SKCM	472
BLCA	424
LIHC	416
LUSC	378
STAD	371
KIRC	342
CESC	308
COAD	297
KIRP	297
SARC	262

Table S2. PR-AUC for detecting methylation-driving genes on imputed cancer datasets over 16 cancer types.

Missing rate	BRCA					THCA				
	SVD	TOBMI	Lasso	TDimpute-self	TDimpute	SVD	TOBMI	Lasso	TDimpute-self	TDimpute
10%	**1**	0.994	0.99	**1**	**1**	**0.998**	0.99	0.99	**0.998**	**0.998**
30%	0.978	0.972	0.956	0.988	**0.99**	0.98	0.97	0.966	**0.986**	**0.986**
50%	0.94	0.926	0.898	0.97	**0.97**	0.96	0.92	0.91	**0.964**	**0.964**
70%	0.872	0.862	0.832	0.932	**0.94**	0.926	0.854	0.86	0.932	**0.934**
90%	0.76	0.736	0.758	0.826	**0.854**	0.828	0.754	0.778	0.84	**0.856**
Missing rate	PRAD					LUAD				
	SVD	TOBMI	Lasso	TDimpute-self	TDimpute	SVD	TOBMI	Lasso	TDimpute-self	TDimpute
10%	0.994	0.992	0.99	**0.996**	**0.996**	0.982	0.986	0.98	**0.99**	**0.99**
30%	0.978	0.974	0.96	0.98	**0.984**	0.93	0.95	0.914	0.956	**0.964**
50%	0.946	0.93	0.9	0.958	**0.962**	0.86	0.89	0.788	0.908	**0.928**
70%	0.9	0.834	0.794	0.918	**0.926**	0.76	0.782	0.648	0.812	**0.85**
90%	0.768	0.676	0.69	0.792	**0.838**	0.554	0.602	0.546	0.606	**0.714**
Missing rate	LIHC					LUSC				
	SVD	TOBMI	Lasso	TDimpute-self	TDimpute	SVD	TOBMI	Lasso	TDimpute-self	TDimpute
10%	**0.99**	**0.99**	0.988	**0.99**	**0.99**	0.978	0.986	0.984	**0.99**	**0.99**
30%	0.954	0.95	0.94	0.968	**0.97**	0.92	0.948	0.938	0.96	**0.964**
50%	0.904	0.896	0.892	0.928	**0.936**	0.846	0.874	0.872	0.906	**0.928**
70%	0.836	0.814	0.83	0.866	**0.882**	0.73	0.77	0.776	0.824	**0.862**
90%	0.686	0.646	0.664	0.728	**0.774**	0.522	0.572	0.638	0.648	**0.738**
Missing rate	CESC					KIRP				
	SVD	TOBMI	Lasso	TDimpute-self	TDimpute	SVD	TOBMI	Lasso	TDimpute-self	TDimpute
10%	0.986	**0.988**	0.986	**0.988**	**0.988**	0.968	0.97	0.962	0.98	**0.984**
30%	0.94	0.944	0.938	0.96	**0.966**	0.92	0.924	0.9	0.942	**0.952**
50%	0.87	0.876	0.858	0.91	**0.928**	0.85	0.852	0.832	0.888	**0.914**
70%	0.758	0.746	0.76	0.814	**0.856**	0.764	0.75	0.75	0.804	**0.85**
90%	0.582	0.59	0.622	0.622	**0.748**	0.616	0.602	0.652	0.642	**0.744**
Missing rate	LGG					HNSC				
	SVD	TOBMI	Lasso	TDimpute-self	TDimpute	SVD	TOBMI	Lasso	TDimpute-self	TDimpute
10%	0.99	0.99	0.99	0.994	**1**	0.99	0.99	0.99	0.992	**0.994**
30%	0.966	0.964	0.948	0.976	**0.98**	0.958	0.956	0.94	0.972	**0.98**
50%	0.916	0.908	0.882	0.952	**0.956**	0.89	0.888	0.87	0.94	**0.952**
70%	0.814	0.816	0.806	0.898	**0.906**	0.778	0.776	0.77	0.856	**0.896**
90%	0.714	0.676	0.706	0.722	**0.77**	0.588	0.576	0.644	0.62	**0.762**
Missing rate	SKCM					BLCA				
	SVD	TOBMI	Lasso	TDimpute-self	TDimpute	SVD	TOBMI	Lasso	TDimpute-self	TDimpute
10%	0.99	0.994	0.992	**0.996**	**0.996**	0.99	0.99	0.99	**0.992**	**0.992**
30%	0.964	0.97	0.968	0.98	**0.982**	0.962	0.966	0.964	0.974	**0.978**
50%	0.894	0.91	0.918	0.942	**0.952**	0.92	0.922	0.926	0.944	**0.952**
70%	0.78	0.798	0.85	0.866	**0.9**	0.86	0.862	0.89	0.896	**0.916**
90%	0.65	0.64	0.712	0.676	**0.77**	0.726	0.726	0.8	0.786	**0.838**
Missing rate	STAD					KIRC				
	SVD	TOBMI	Lasso	TDimpute-self	TDimpute	SVD	TOBMI	Lasso	TDimpute-self	TDimpute
10%	0.992	0.992	0.99	**0.996**	**0.996**	0.98	0.974	0.972	**0.986**	**0.986**
30%	0.97	0.976	0.962	0.984	**0.986**	0.928	0.898	0.88	0.938	**0.948**
50%	0.94	0.952	0.912	0.964	**0.968**	0.852	0.792	0.752	0.866	**0.892**
70%	0.894	0.91	0.846	0.934	**0.94**	0.742	0.646	0.622	0.764	**0.822**
90%	0.78	0.774	0.738	0.832	**0.866**	0.56	0.518	0.516	0.56	**0.706**
Missing rate	COAD					SARC				
	SVD	TOBMI	Lasso	TDimpute-self	TDimpute	SVD	TOBMI	Lasso	TDimpute-self	TDimpute
10%	0.992	0.992	0.99	0.994	**0.996**	0.98	0.988	0.98	**0.99**	**0.99**
30%	0.968	0.97	0.954	0.978	**0.98**	0.946	0.958	0.942	0.964	**0.97**
50%	0.926	0.918	0.89	0.948	**0.954**	0.892	0.912	0.89	0.926	**0.934**
70%	0.856	0.852	0.83	0.902	**0.922**	0.82	0.838	0.836	0.86	**0.876**
90%	0.642	0.674	0.634	0.764	**0.856**	0.664	0.658	0.712	0.72	**0.772**

The results are averaged over 5 random replicas. Best results are highlighted in bold face.

Table S3.1. Overlap of top 100 methylation-driving genes from imputed dataset and full dataset

Missing rate	SVD	TOBMI	Lasso	TDimpute-self	TDimpute
10%	89.61*	87.66*	86.45*	91.59	**91.98**
30%	76.03*	71.83*	68.15*	83.14*	**84.00**
50%	62.44*	55.21*	52.43*	73.09*	**76.24**
70%	45.43*	37.70*	37.70*	57.96*	**65.66**
90%	24.83	18.48*	22.56*	26.73*	**50.43**

The results are averaged over 5 random replicas. Best results are highlighted in bold face.
* indicates statistical significance (p-value < 0.05) between TDimpute and other methods.

Table S3.2. Overlap of top 100 methylation-driving genes between imputed dataset and full dataset over 16 cancer types

Missing rate	BRCA					THCA				
	SVD	TOBMI	Lasso	TDimpute-self	TDimpute	SVD	TOBMI	Lasso	TDimpute-self	TDimpute
10%	96.2	93	92.8	95.8	**95.2**	**97.6**	96	94.8	97.4	**97.6**
30%	93.2	82.8	80.2	92.2	**93.4**	94	87.2	88.8	**94.8**	94.2
50%	86.8	69.4	71.8	**90.4**	88.8	88	68.2	77.2	89.8	**90.8**
70%	73.6	44	53.4	81.6	**85.8**	75.6	57.6	66.8	83.2	**85.4**
90%	34.6	9.6	21.4	45.6	**66.2**	34.2	31.6	41.2	52.4	**71**
Missing rate	PRAD					LUAD				
	SVD	TOBMI	Lasso	TDimpute-self	TDimpute	SVD	TOBMI	Lasso	TDimpute-self	TDimpute
10%	84.4	81	77.6	89	**90.2**	93.8	92.4	77.6	**94.6**	93.8
30%	61.8	59.4	54.6	**78.8**	77.4	84.4	82.4	54.6	87.2	**87.6**
50%	48.4	47.8	42.6	**66.2**	64.4	77.8	73.2	42.6	82.2	**83.6**
70%	38.6	37	35.2	51.2	**52.6**	63.8	63.4	35.2	73.8	**77.8**
90%	26.2	21.8	27	26.2	**36**	40.2	38.6	27	38.6	**68.4**
Missing rate	LIHC					LUSC				
	SVD	TOBMI	Lasso	TDimpute-self	TDimpute	SVD	TOBMI	Lasso	TDimpute-self	TDimpute
10%	85.6	87.2	91.4	91.4	**92**	94.6	93.2	93.6	94	**95.4**
30%	72.6	72.4	68.8	80.6	**81.6**	86.4	83.4	75	**89.6**	89
50%	64.8	53.2	50	72.4	**76.2**	75.6	71	55.8	82.6	**83.8**
70%	50	25.2	24.6	56.6	**60**	51.4	46.2	32	75.2	**78.4**
90%	27.4	8.2	11.6	31	**51.2**	18.2	16.2	13.6	23.2	**65**
Missing rate	CESC					KIRP				
	SVD	TOBMI	Lasso	TDimpute-self	TDimpute	SVD	TOBMI	Lasso	TDimpute-self	TDimpute
10%	89.2	82.4	81.2	**91**	90.2	83.2	79.4	78.6	84.8	**87.2**
30%	73.2	64.2	60.8	78.8	**82.2**	68.4	57.8	60.2	74	**78.2**
50%	47	40.8	44.6	57.6	**70.4**	53.2	45	50.2	59.6	**67**
70%	30.4	30.6	35.6	33.2	**54.2**	37.6	22.8	29.4	42.8	**54**
90%	18.2	16.4	20.4	17.8	**38.4**	16.2	10.6	17.2	12	**36.6**
Missing rate	LGG					HNSC				
	SVD	TOBMI	Lasso	TDimpute-self	TDimpute	SVD	TOBMI	Lasso	TDimpute-self	TDimpute
10%	81.6	81.8	77.2	88.4	**89.8**	91.6	90.6	86.8	**92.2**	92
30%	67.2	63.6	56.8	75.8	**79.2**	80	79.8	66.4	**84.6**	83.4
50%	59.8	57.4	52.6	64	**67.6**	66.2	66.6	44.8	77.6	**80.4**
70%	48.2	45.2	48.6	52.2	**53**	42.6	52.6	31.2	65.6	**72.8**
90%	33.2	21.4	38	26.4	**34.6**	18.2	30.6	16.4	22.2	**58.2**
Missing rate	SKCM					BLCA				
	SVD	TOBMI	Lasso	TDimpute-self	TDimpute	SVD	TOBMI	Lasso	TDimpute-self	TDimpute
10%	95.6	94.8	95.8	**96.4**	**96.4**	94.4	92.2	92	**95**	94.6
30%	86.2	83.6	88.6	91.4	**92.6**	85.6	78.6	75.4	**90.2**	90
50%	78.4	72	81.8	86.4	**89.6**	72	55.8	54.8	85.6	**86.8**
70%	60.4	47.2	68.2	71	**78.4**	51	36.2	37.2	72.2	**80**
90%	36	24.4	44.2	43.8	**64.2**	33.4	23.8	18	29.8	**67**
Missing rate	STAD					KIRC				
	SVD	TOBMI	Lasso	TDimpute-self	TDimpute	SVD	TOBMI	Lasso	TDimpute-self	TDimpute
10%	84.4	84.2	77.2	**87.4**	**87.4**	88.4	85.2	81	89.6	**91**
30%	69.4	69.8	60.8	**81**	79	71.4	66.6	56.6	74.8	**77.8**
50%	54.4	55.6	47.8	**69.4**	68.8	55.4	47.4	37.8	60.8	**65.6**
70%	36.4	41.6	37.8	55.2	**61.6**	36.8	30.8	25.6	45.4	**54**
90%	19.4	15.2	32	25.8	**44**	24.2	19.8	20.4	13.2	**39.2**
Missing rate	COAD					SARC				
	SVD	TOBMI	Lasso	TDimpute-self	TDimpute	SVD	TOBMI	Lasso	TDimpute-self	TDimpute
10%	83.4	81.4	82.6	**86.8**	86.4	89.8	87.8	91.2	91.6	**92.4**
30%	47.6	55	49.2	71.4	**72**	75	62.6	79.4	85	**86.4**
50%	19	31.2	23.8	54.8	**60.4**	52.2	28.8	58.6	70	**75.6**
70%	5.4	12.6	10	27.4	**42.6**	25	10.2	36.8	40.8	**60**
90%	3.4	2	3.2	1.2	**23.8**	14.2	5.4	6	18.4	**43**

The results are averaged over 5 random replicas. Best results are highlighted in bold face.

Table S4. PR-AUC for detecting significantly prognostic gene on imputed datasets over 16 cancer types.

Missing rate	BRCA					THCA				
	SVD	TOBMI	Lasso	TDimpute-self	TDimpute	SVD	TOBMI	Lasso	TDimpute-self	TDimpute
10%	0.892	0.918	0.91	0.932	**0.938**	0.878	0.884	0.882	0.9	**0.906**
30%	0.738	0.82	0.802	0.844	**0.852**	0.658	0.642	0.66	0.714	**0.718**
50%	0.548	0.664	0.63	0.71	**0.736**	0.466	0.498	0.514	0.556	**0.588**
70%	0.382	0.446	0.39	0.526	**0.542**	0.344	0.352	0.358	0.42	**0.468**
90%	0.306	0.358	0.294	0.376	**0.406**	0.184	0.166	0.17	0.178	**0.238**
Missing rate	PRAD					LUAD				
	SVD	TOBMI	Lasso	TDimpute-self	TDimpute	SVD	TOBMI	Lasso	TDimpute-self	TDimpute
10%	**0.936**	**0.936**	0.932	0.924	**0.936**	0.936	0.94	0.942	**0.952**	**0.952**
30%	0.65	0.648	0.648	0.638	**0.67**	0.814	0.846	0.83	0.87	**0.882**
50%	0.42	0.432	0.412	0.43	**0.46**	0.676	0.706	0.694	0.748	**0.764**
70%	0.294	0.3	0.282	0.3	**0.316**	0.536	0.564	0.544	0.596	**0.654**
90%	0.104	0.092	0.096	0.104	**0.122**	0.36	0.402	0.38	0.398	**0.504**
Missing rate	LIHC					LUSC				
	SVD	TOBMI	Lasso	TDimpute-self	TDimpute	SVD	TOBMI	Lasso	TDimpute-self	TDimpute
10%	0.84	0.832	0.834	0.856	**0.866**	0.822	0.838	0.834	**0.864**	0.86
30%	0.614	0.648	0.642	0.676	**0.704**	0.556	0.544	0.564	0.574	**0.602**
50%	0.44	0.476	0.47	0.508	**0.524**	0.376	0.346	0.37	0.4	**0.43**
70%	0.288	0.308	0.304	0.356	**0.418**	0.22	0.21	0.22	0.236	**0.27**
90%	0.162	0.146	0.154	0.178	**0.248**	0.122	0.106	0.118	0.132	**0.178**
Missing rate	CESC					KIRP				
	SVD	TOBMI	Lasso	TDimpute-self	TDimpute	SVD	TOBMI	Lasso	TDimpute-self	TDimpute
10%	0.932	0.938	0.944	0.946	**0.948**	0.938	0.956	0.944	0.956	**0.96**
30%	0.802	0.816	0.804	0.828	**0.842**	0.812	0.864	0.848	0.866	**0.884**
50%	0.662	0.666	0.66	0.694	**0.708**	0.752	0.754	0.728	0.758	**0.78**
70%	0.47	0.438	0.472	0.516	**0.534**	0.582	0.642	0.592	0.608	**0.658**
90%	0.26	0.23	0.256	0.27	**0.378**	0.352	0.386	0.374	0.376	**0.452**
Missing rate	LGG					HNSC				
	SVD	TOBMI	Lasso	TDimpute-self	TDimpute	SVD	TOBMI	Lasso	TDimpute-self	TDimpute
10%	0.988	0.99	0.99	**0.992**	**0.992**	0.922	0.944	0.944	0.948	**0.952**
30%	0.964	0.966	0.966	0.97	**0.97**	0.754	0.82	0.808	0.83	**0.84**
50%	0.926	0.924	0.928	0.932	**0.938**	0.572	0.66	0.642	0.688	**0.728**
70%	0.872	0.868	0.886	0.878	**0.886**	0.448	0.496	0.47	0.518	**0.57**
90%	0.796	0.728	0.788	0.756	**0.79**	0.292	0.268	0.27	0.31	**0.418**
Missing rate	SKCM					BLCA				
	SVD	TOBMI	Lasso	TDimpute-self	TDimpute	SVD	TOBMI	Lasso	TDimpute-self	TDimpute
10%	0.184	0.184	**0.924**	0.184	0.182	0.866	0.91	0.914	0.922	**0.926**
30%	0.182	0.184	**0.79**	0.182	0.182	0.592	0.732	0.738	0.764	**0.778**
50%	0.188	0.188	**0.67**	0.182	0.182	0.462	0.562	0.57	0.652	**0.68**
70%	0.188	0.188	**0.604**	0.182	0.18	0.342	0.412	0.434	0.496	**0.554**
90%	0.198	0.202	**0.404**	0.192	0.184	0.238	0.218	0.258	0.292	**0.424**
Missing rate	STAD					KIRC				
	SVD	TOBMI	Lasso	TDimpute-self	TDimpute	SVD	TOBMI	Lasso	TDimpute-self	TDimpute
10%	0.876	0.862	0.852	0.884	**0.886**	0.984	0.99	0.99	0.99	**0.992**
30%	0.544	0.6	0.58	0.65	**0.656**	0.944	0.962	0.962	0.966	**0.968**
50%	0.372	0.424	0.394	**0.46**	0.454	0.898	0.918	0.912	0.934	**0.938**
70%	0.158	0.224	0.202	0.246	**0.248**	0.802	0.84	0.826	0.872	**0.882**
90%	0.078	0.112	0.09	0.116	**0.132**	0.648	0.674	0.662	0.664	**0.726**
Missing rate	COAD					SARC				
	SVD	TOBMI	Lasso	TDimpute-self	TDimpute	SVD	TOBMI	Lasso	TDimpute-self	TDimpute
10%	0.778	0.804	0.816	0.824	**0.836**	0.926	0.936	0.932	0.94	**0.942**
30%	0.42	0.458	0.446	0.462	**0.512**	0.738	0.79	0.784	0.8	**0.816**
50%	0.202	0.228	0.224	0.23	**0.298**	0.552	0.614	0.616	0.618	**0.658**
70%	0.118	0.122	0.126	0.136	**0.194**	0.424	0.454	0.476	0.472	**0.528**
90%	0.058	0.058	0.058	0.064	**0.106**	0.246	0.29	0.292	0.316	**0.398**

The results are averaged over 5 random replicas. Best results are highlighted in bold face.

Table S5.1. Overlap of top 100 significantly prognostic genes identified by univariate Cox model between imputed datasets and full datasets.

Missing rate	SVD	TOBMI	Lasso	TDimpute-self	TDimpute
10%	73.5*	74.8*	68.69*	76.4*	**77.5**
30%	50.6*	52.3*	46.24*	56.6	**57.3**
50%	37.6*	37.8*	31.43*	44.2	**44.9**
70%	27.3*	26.6*	21.04*	33.2*	**35.2**
90%	15.8*	16*	11.59*	20.6*	**24.6**

The results are averaged over 5 random replicas. Best results are highlighted in bold face.
* indicates statistical significance (p-value < 0.05) between TDimpute and other methods.

Table S5.2. Overlap of top 100 prognostic genes identified by univariate Cox model between imputed dataset and full dataset over 16 cancer types.

Missing rate	BRCA					THCA				
	SVD	TOBMI	Lasso	TDimpute-self	TDimpute	SVD	TOBMI	Lasso	TDimpute-self	TDimpute
10%	63.4	70	67	**73**	72.4	69.2	68.2	71.2	70.2	**70.6**
30%	40.6	52.2	49.4	52.8	**54.8**	46	42.2	44.2	**49.4**	49
50%	27.2	33.8	28.4	36.2	**39.4**	31.2	28.2	29.4	33.4	**35.2**
70%	13.8	18	13	21.2	**24**	22.2	18.4	18.4	**25.2**	23.8
90%	6.2	5.8	4.8	9.4	**12.6**	**16.4**	8	7.2	12.8	15.4
Missing rate	PRAD					LUAD				
	SVD	TOBMI	Lasso	TDimpute-self	TDimpute	SVD	TOBMI	Lasso	TDimpute-self	TDimpute
10%	86	85.4	85.6	83.4	**85.6**	75.8	76.6	75	79.8	**82.8**
30%	51.4	49.2	50.6	52	**54.6**	58	53.8	53.4	**66**	64
50%	29.4	29	29.2	32.6	**33.4**	48	41.4	40.4	51.8	**53**
70%	18.6	19	16	19	**19.4**	33.2	27	26.4	37.8	**47.2**
90%	2.8	3.4	3.4	3.6	**4.4**	19.4	9.8	14	21	**32**
Missing rate	LIHC					LUSC				
	SVD	TOBMI	Lasso	TDimpute-self	TDimpute	SVD	TOBMI	Lasso	TDimpute-self	TDimpute
10%	65	64.2	63	67.8	**68**	59.8	63	63	63.4	**68.8**
30%	45.2	48	48.2	**53.2**	50.2	32.6	35.4	34.8	**38.6**	**38.6**
50%	29.8	32.2	33.4	**37**	34.2	20.4	20.2	20.2	23.6	**24.8**
70%	**23.2**	19	21.4	21.4	22.2	10.4	9.4	10	11	**14.2**
90%	7.2	4.6	5.8	8	**13**	2.2	2	2.8	2.8	**6**
Missing rate	CESC					KIRP				
	SVD	TOBMI	Lasso	TDimpute-self	TDimpute	SVD	TOBMI	Lasso	TDimpute-self	TDimpute
10%	81.6	79.6	82.8	80.2	**82.2**	88.6	87.8	77.4	87.8	**88.8**
30%	58.8	60.4	59.2	59	**63.6**	**85.2**	68	53.2	85	81
50%	43.6	47.2	47	48.4	**50.4**	**83.8**	55.6	36.8	82.2	77.6
70%	21.2	21.4	26	**31.2**	30.2	80.2	57.4	30.6	**83.2**	82
90%	4.4	5.4	5.4	8.4	**13**	49.4	49.6	26.8	59.4	**67.4**
Missing rate	LGG					HNSC				
	SVD	TOBMI	Lasso	TDimpute-self	TDimpute	SVD	TOBMI	Lasso	TDimpute-self	TDimpute
10%	90.2	89.6	76.4	91.2	**92.6**	69	70.6	71.8	**77.2**	**77.2**
30%	87.4	87.2	66.4	**88.6**	88	47	50.4	48.4	52.2	**56**
50%	88	86.4	55.2	**89**	88	28.2	33.6	31.2	37.6	**42.4**
70%	84.4	90.4	61.4	90.6	**91.2**	16.4	18.8	18	22.2	**28.4**
90%	80.8	85.4	65.2	**92.6**	90	6.4	3.6	4.4	7.2	**13.8**
Missing rate	SKCM					BLCA				
	SVD	TOBMI	Lasso	TDimpute-self	TDimpute	SVD	TOBMI	Lasso	TDimpute-self	TDimpute
10%	61	66.2	0	**66.4**	65.2	69.4	74.6	74.2	74.8	**75.6**
30%	30.2	37.2	0.8	37.4	**39.2**	37.6	47.6	49.4	**54.6**	54.2
50%	11.8	15.2	0.6	**21**	20.4	21.2	26.8	31	**41.8**	41.6
70%	3.2	6.2	0	8.6	**11.8**	14.6	15.2	18	27.2	**31.8**
90%	1.8	0.4	0	1.4	**4.4**	7.8	4.6	7.6	10.2	**20.6**
Missing rate	STAD					KIRC				
	SVD	TOBMI	Lasso	TDimpute-self	TDimpute	SVD	TOBMI	Lasso	TDimpute-self	TDimpute
10%	71.6	70.2	69	72.2	**73**	91.4	89.6	84.6	**91.4**	91.2
30%	37.8	42.4	42.2	44.4	**45.2**	75.8	72.6	55	81.8	**83.2**
50%	25.4	26.4	25.2	25.8	**29.6**	65.4	64.4	36.6	**81.2**	78.2
70%	8.2	9.4	7.2	12.2	**12.6**	57.8	58.6	32.8	**76.6**	72.4
90%	**3.8**	3.4	1.8	3.2	3.6	38.6	49.8	24.4	**63.6**	62
Missing rate	COAD					SARC				
	SVD	TOBMI	Lasso	TDimpute-self	TDimpute	SVD	TOBMI	Lasso	TDimpute-self	TDimpute
10%	59.8	63.4	62.2	64.8	**65.6**	73.4	77	75.8	79.4	**79.6**
30%	30.4	33	30.8	32.8	**37.8**	45.8	57.2	53.8	57.8	**58**
50%	18.4	**21**	20.4	19.2	20.6	30	43	37.8	46	**49**
70%	7.8	6.6	8.4	9.2	**15**	20.8	30	29	35.2	**37.2**
90%	2	2.4	1.4	2.4	**8.2**	3.2	18.2	10.4	23.2	**27**

The results are averaged over 5 random replicas. Best results are highlighted in bold face.

Table S6. The enrichment factors of the top 100 ranked genes in the gene list from The Human Protein Atlas across 16 cancer types

Missing rate	BRCA					THCA				
	SVD	TOBMI	Lasso	TDimpute-self	TDimpute	SVD	TOBMI	Lasso	TDimpute-self	TDimpute
10%	7.637	8.625	8.098	8.493	**8.888**	8.664	9.102	**9.322**	8.554	9.102
30%	4.411	**6.584**	5.925	6.452	6.386	5.045	6.251	6.141	**6.799**	6.361
50%	2.765	4.740	3.489	**5.267**	**5.267**	3.400	4.058	4.167	**4.496**	**4.496**
70%	1.383	2.436	1.185	3.226	**3.424**	2.303	2.961	2.632	**3.509**	2.522
90%	0.790	0.988	0.593	1.580	**1.843**	1.755	1.206	0.987	**2.303**	1.864
Missing rate	PRAD					LUAD				
	SVD	TOBMI	Lasso	TDimpute-self	TDimpute	SVD	TOBMI	Lasso	TDimpute-self	TDimpute
10%	14.60	**15.08**	**15.08**	13.40	14.12	8.476	8.476	8.242	**8.944**	**8.944**
30%	8.86	**9.57**	9.33	8.86	8.14	6.839	6.079	5.962	**7.541**	**7.541**
50%	4.31	**5.74**	5.50	4.55	4.55	5.436	4.618	4.559	5.904	**5.962**
70%	3.11	**4.79**	3.35	3.59	2.15	3.566	2.923	3.040	4.150	**5.904**
90%	0.96	1.68	1.44	**2.15**	1.44	2.455	0.877	1.695	2.747	**4.092**
Missing rate	LIHC					LUSC				
	SVD	TOBMI	Lasso	TDimpute-self	TDimpute	SVD	TOBMI	Lasso	TDimpute-self	TDimpute
10%	1.004	0.951	0.977	1.017	**1.030**	1.403	1.637	1.461	1.578	**1.695**
30%	**0.700**	0.647	0.687	**0.700**	0.674	0.760	1.169	0.877	1.286	**1.461**
50%	0.396	0.449	**0.489**	0.436	0.370	0.526	0.760	0.468	0.701	**0.818**
70%	0.304	0.225	**0.330**	0.225	0.277	0.292	**0.526**	0.234	0.292	0.468
90%	**0.119**	0.000	0.066	0.092	**0.119**	0.000	**0.058**	0.000	0.000	**0.058**
Missing rate	CESC					KIRP				
	SVD	TOBMI	Lasso	TDimpute-self	TDimpute	SVD	TOBMI	Lasso	TDimpute-self	TDimpute
10%	13.09	13.19	13.30	13.19	**13.51**	0.363	0.363	0.300	**0.370**	0.363
30%	9.62	10.62	9.88	10.04	**10.83**	**0.351**	0.287	0.223	**0.351**	0.332
50%	6.73	8.15	7.78	7.99	**8.83**	**0.370**	0.236	0.134	**0.370**	0.338
70%	2.58	3.68	3.94	4.73	**5.10**	0.332	0.300	0.147	**0.363**	0.338
90%	0.47	0.68	0.95	1.31	**2.05**	0.217	0.217	0.108	0.255	**0.281**
Missing rate	LGG					HNSC				
	SVD	TOBMI	Lasso	TDimpute-self	TDimpute	SVD	TOBMI	Lasso	TDimpute-self	TDimpute
10%	NA					6.29	6.92	6.97	**7.45**	7.40
30%						3.84	4.71	4.71	5.00	**5.53**
50%						1.97	3.32	2.64	3.80	**4.23**
70%						0.96	1.63	1.35	2.21	**2.64**
90%						0.34	0.19	0.24	0.72	**1.01**
Missing rate	SKCM					BLCA				
	SVD	TOBMI	Lasso	TDimpute-self	TDimpute	SVD	TOBMI	Lasso	TDimpute-self	TDimpute
10%	2.78	**3.53**	0	2.78	3.16	6.65	7.24	7.45	7.28	**7.56**
30%	1.11	**2.41**	0	2.23	1.86	3.13	4.94	4.91	**5.74**	5.71
50%	0.19	1.11	0	0.93	**1.49**	1.67	2.82	2.85	**4.42**	4.14
70%	0.00	0.74	0	0.00	**1.11**	0.91	1.57	1.85	2.79	**3.31**
90%	0.00	0.00	0	0.00	**0.37**	0.59	0.35	0.59	0.97	**2.26**
Missing rate	STAD					KIRC				
	SVD	TOBMI	Lasso	TDimpute-self	TDimpute	SVD	TOBMI	Lasso	TDimpute-self	TDimpute
10%	5.09	5.09	5.09	**5.47**	5.35	0.281	0.281	0.268	**0.287**	**0.287**
30%	2.80	2.80	2.67	3.18	**3.69**	0.255	0.261	0.166	0.255	**0.268**
50%	0.89	1.65	1.40	1.53	**1.78**	0.210	0.223	0.077	0.268	**0.274**
70%	0.00	0.38	0.51	**0.89**	0.76	0.185	0.204	0.089	0.249	**0.268**
90%	0.00	0.13	0.13	0.00	**0.25**	0.096	0.185	0.038	0.217	**0.236**
Missing rate	COAD					SARC				
	SVD	TOBMI	Lasso	TDimpute-self	TDimpute	SVD	TOBMI	Lasso	TDimpute-self	TDimpute
10%	1.02	1.09	1.09	1.15	**1.28**	NA				
30%	0.77	**0.83**	**0.51**	0.70	0.77					
50%	0.32	0.45	0.26	**0.51**	0.32					
70%	0.00	0.00	0.00	0.00	**0.19**					
90%	0.06	0.06	0.00	0.06	**0.13**					

The results are averaged over 5 random replicas. Best results are highlighted in bold face.

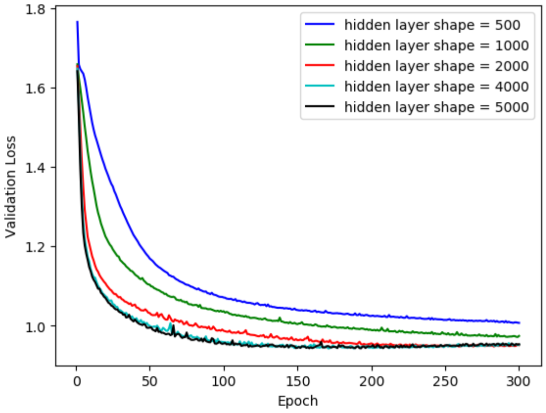

RMSE for cross-validation (5CV) when using different hyper-parameters.

the number of hidden layers	hidden layer shape	epoch 50	100	150	**300**	500
**1**	500	1.253	1.131	1.09	1.052	1.04
	1000	1.146	1.076	1.047	1.016	1.013
	2000	1.074	1.029	1.009	0.995	1.009
	**4000**	1.043	1.003	0.991	0.997	1.015
	5000	1.035	0.999	0.988	0.995	1.015
3	(4000, 500, 4000)	1.13	1.067	1.039	1.01	1.011
	(4000, 1000, 4000)	1.107	1.046	1.032	1.011	1.035
	(4000, 2000, 4000)	1.092	1.042	1.021	1.025	1.064
	(5000, 500, 5000)	1.085	1.034	1.021	1.028	1.078
	(5000, 1000, 5000)	1.097	1.052	1.025	1.014	1.04
	(5000, 2000, 5000)	1.124	1.062	1.036	1.008	1.012

Seleced hyper-parameters are highlighted in bold face.

Fig S6 (left). Loss curves for different hidden layer shape and **Table S7** (right). hyper-parameter analysis for hidden layer shape, the number of hidden layers, and training epochs on pan-cancer dataset (excluding BRCA dataset).

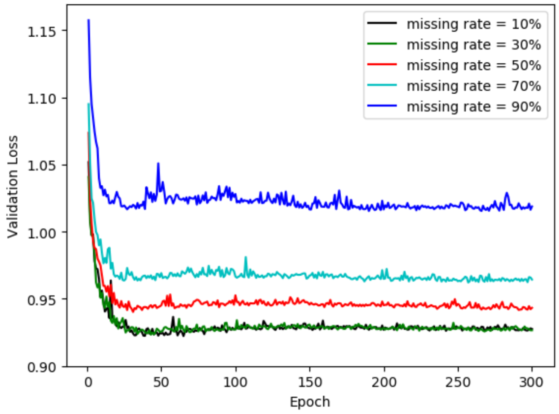

Fig S7. The loss curves of different missing rates on the validation dataset of BRCA
