# Supplementary material for: Imputing missing RNA-sequencing data from DNA methylation by using a transfer learning–based neural network
Source: Gigascience. 2020 Jul 10;9(7):giaa076. doi: 10.1093/gigascience/giaa076 (PMC7350980; doi:10.1093/gigascience/giaa076)
Supplement: giaa076_Supplemental_File [file giaa076_supplemental_file.pdf]

Supplement figures and tables

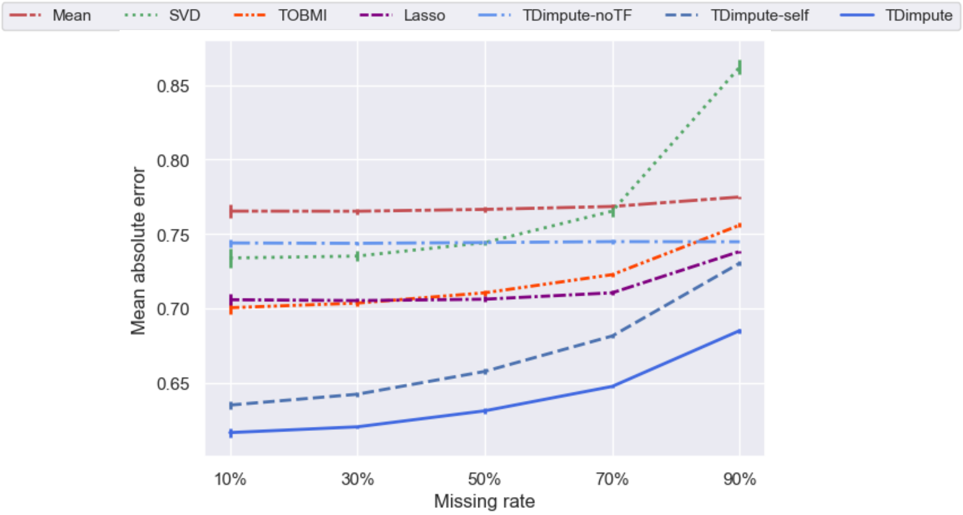

Fig S1. Mean absolute error of each imputation method. Results were averaged across 16 imputed cancer datasets. TDimpute-self indicates the TDimpute trained and predicted on the target cancer dataset. TDimpute-noTF indicates the TDimpute trained on the pan-cancer dataset (excluding the target cancer) and predicted on the target cancer dataset. The error bar shows the standard deviation.

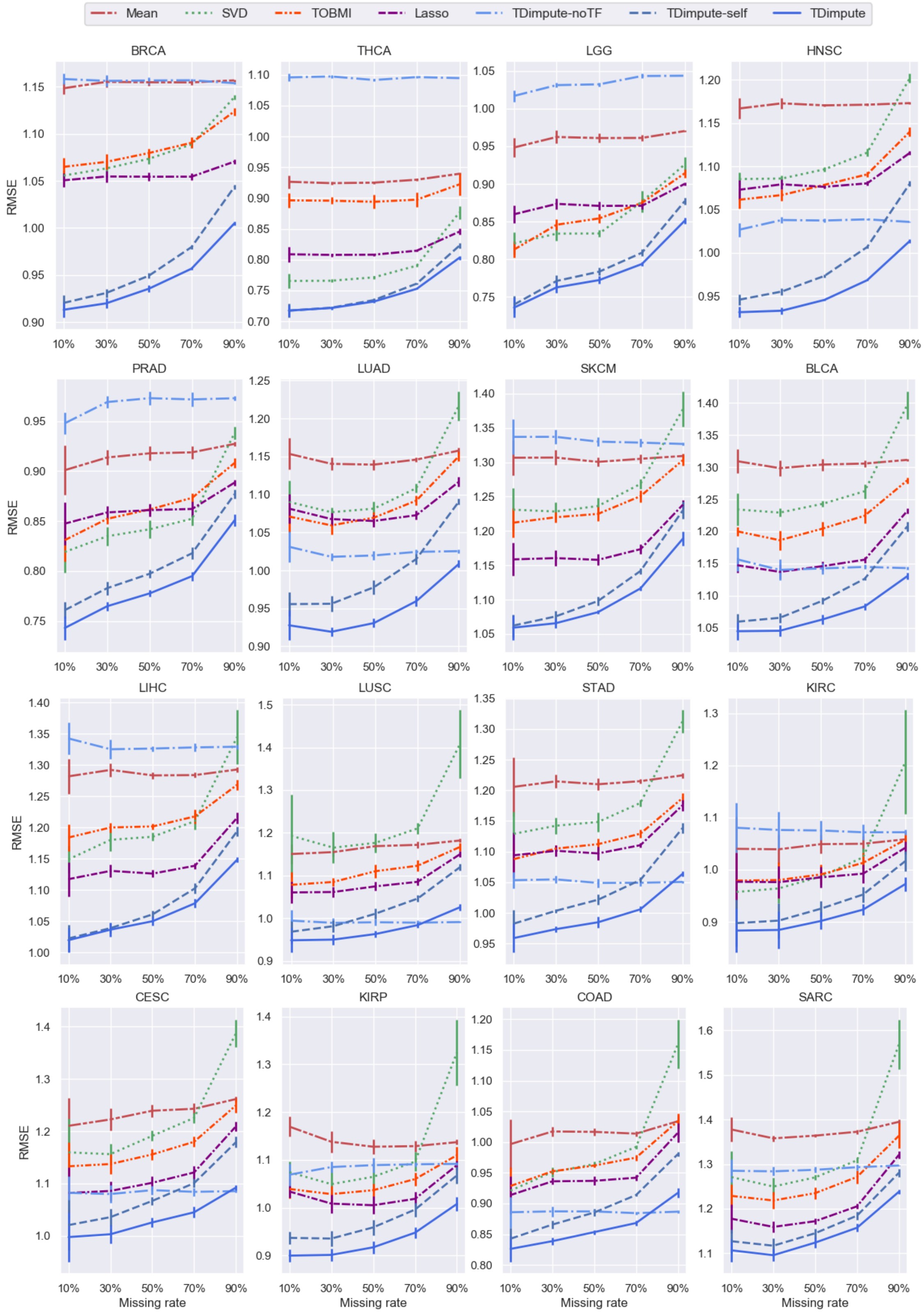

Fig S2. RMSE on 16 imputed cancer datasets with different missing rates. The results were averaged over 5 random replicas. The error bar shows the standard deviation.

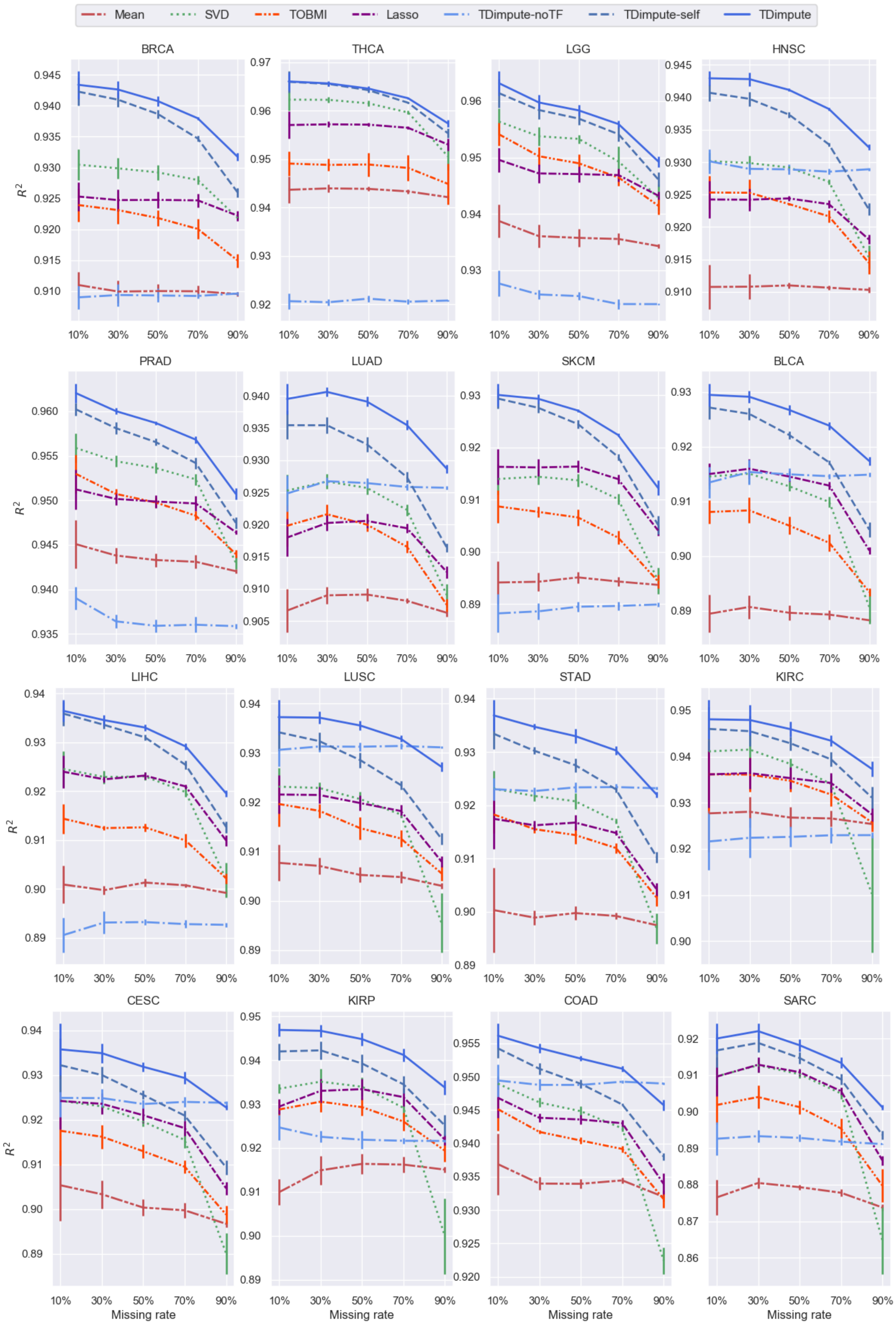

Fig S3. The squared Pearson correlation coefficients R^2 between each sample of the imputed data and the original full data on 16 imputed cancer datasets with different missing rates. The results were averaged over 5 random replicas. The error bar shows the standard deviation.

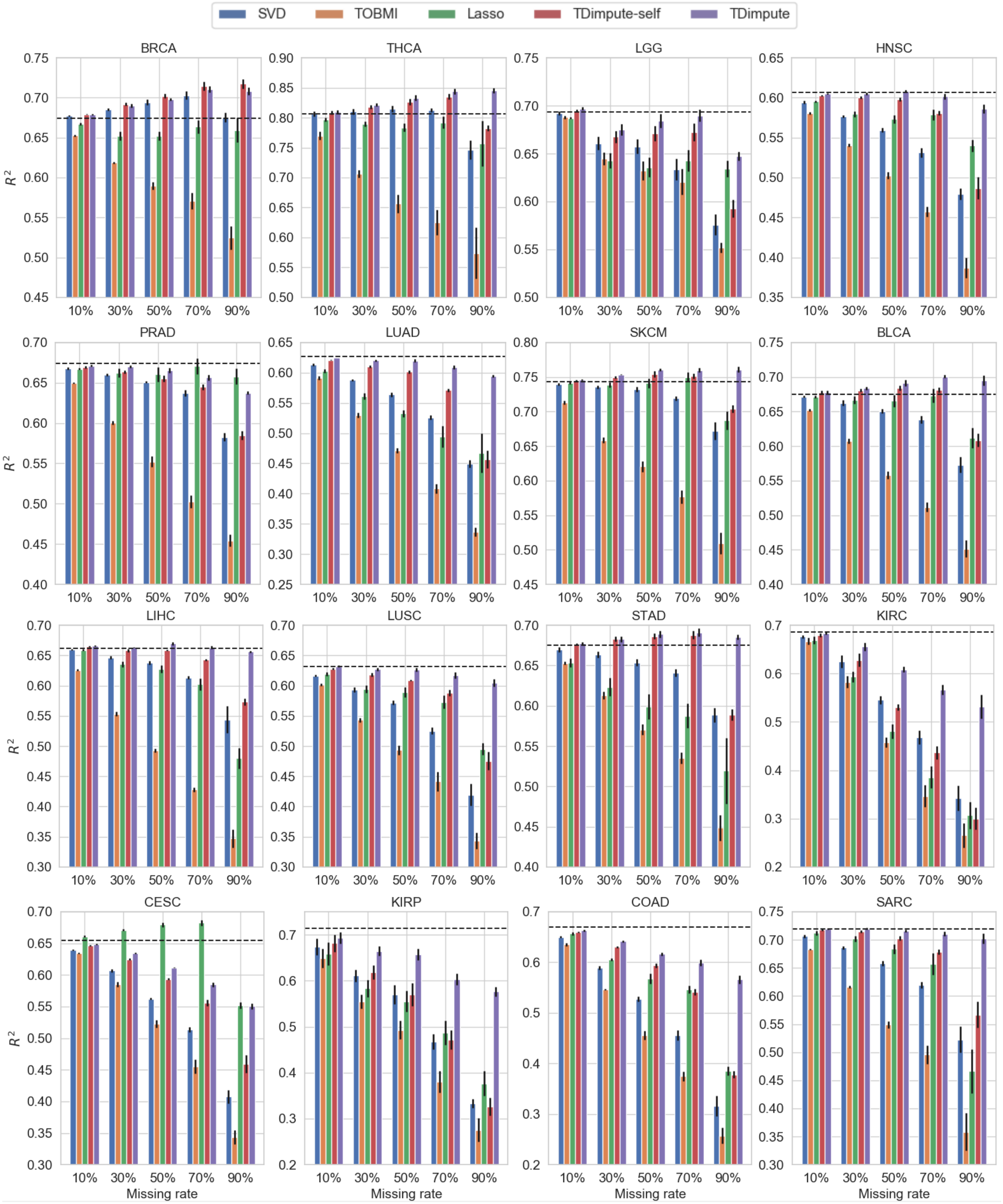

Fig S4. The squared Pearson correlation coefficients R^2 between gene expression and methylation sites on 16 imputed cancer datasets with different missing rates. The results were averaged over 5 random replicas. Dashed black line is drawn as a reference indicating the correlations from the original full dataset. The error bar shows the standard error of the mean.

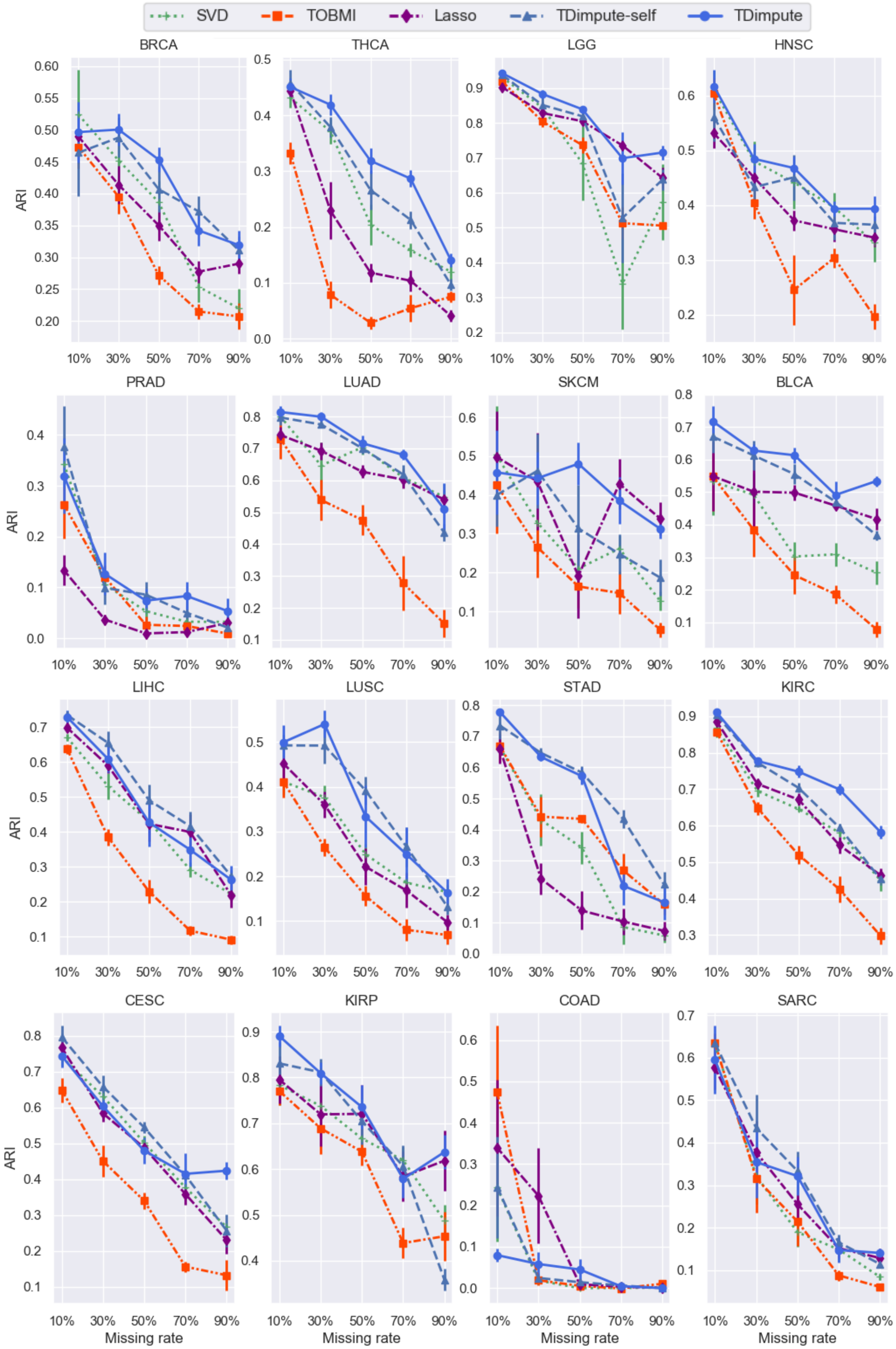

Fig S5. ARI on 16 imputed cancer datasets with different missing rates. The results were averaged over 5 random replicas. The error bar shows the standard error of the mean.

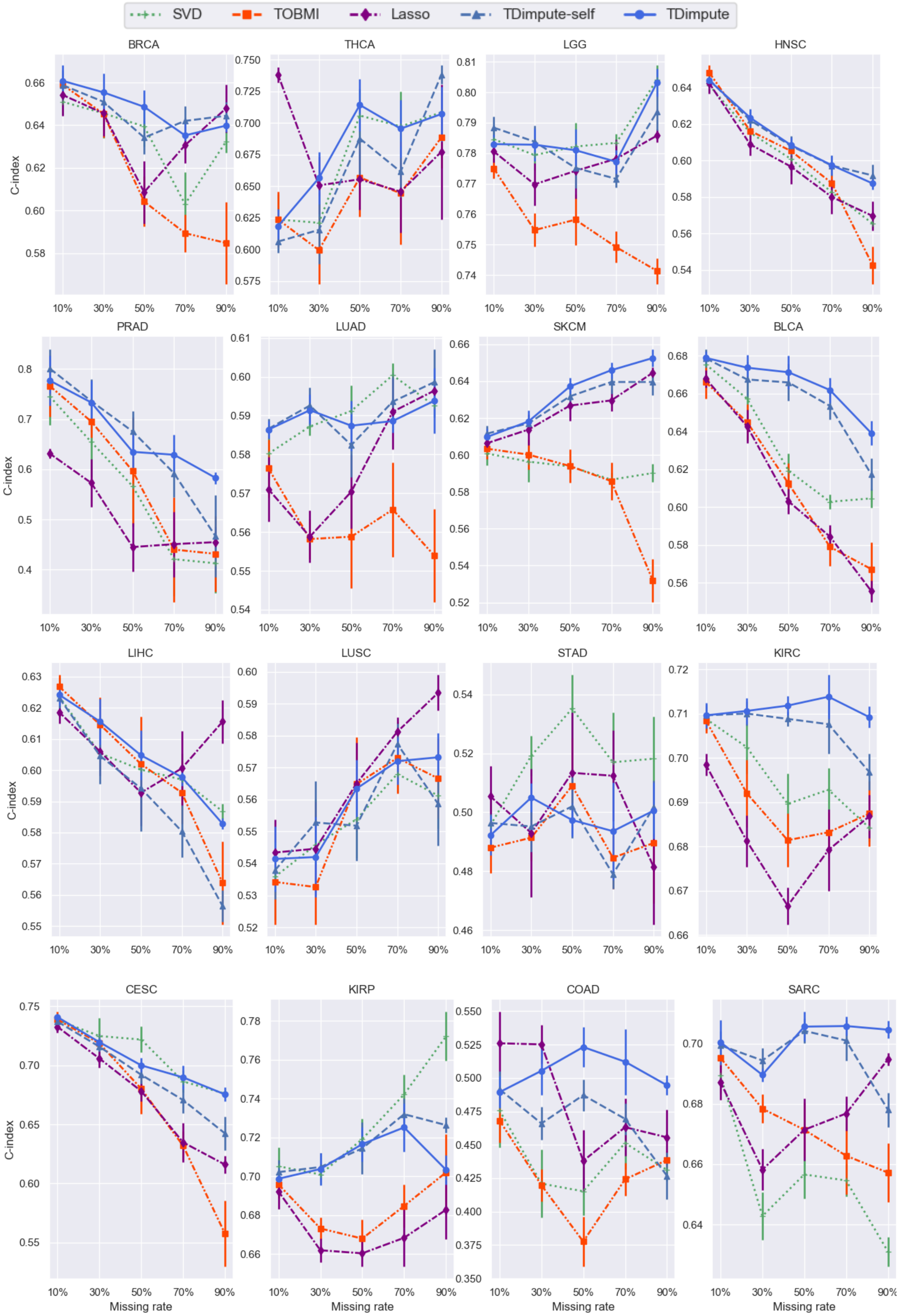

Fig S6. C-index on 16 imputed cancer datasets with different missing rates. The results were averaged over 5 random replicas. The error bar shows the standard error of the mean.

Table S1. PR-AUC for detecting methylation-driving genes on imputed cancer datasets over 16 cancer types.

Missing rate	BRCA						THCA				
	SVD	TOBMI	Lasso	TDimpute-self	TDimpute		SVD	TOBMI	Lasso	TDimpute-self	TDimpute
10%	**1**	0.994	0.99	**1**	**1**		**0.998**	0.99	0.99	**0.998**	**0.998**
30%	0.978	0.972	0.956	0.988	**0.99**		0.98	0.97	0.966	**0.986**	**0.986**
50%	0.94	0.926	0.898	0.97	**0.97**		0.96	0.92	0.91	**0.964**	**0.964**
70%	0.872	0.862	0.832	0.932	**0.94**		0.926	0.854	0.86	0.932	**0.934**
90%	0.76	0.736	0.758	0.826	**0.854**		0.828	0.754	0.778	0.84	**0.856**
Missing rate	PRAD						LUAD				
	SVD	TOBMI	Lasso	TDimpute-self	TDimpute		SVD	TOBMI	Lasso	TDimpute-self	TDimpute
10%	0.994	0.992	0.99	**0.996**	**0.996**		0.982	0.986	0.98	**0.99**	**0.99**
30%	0.978	0.974	0.96	0.98	**0.984**		0.93	0.95	0.914	0.956	**0.964**
50%	0.946	0.93	0.9	0.958	**0.962**		0.86	0.89	0.788	0.908	**0.928**
70%	0.9	0.834	0.794	0.918	**0.926**		0.76	0.782	0.648	0.812	**0.85**
90%	0.768	0.676	0.69	0.792	**0.838**		0.554	0.602	0.546	0.606	**0.714**
Missing rate	LIHC						LUSC				
	SVD	TOBMI	Lasso	TDimpute-self	TDimpute		SVD	TOBMI	Lasso	TDimpute-self	TDimpute
10%	**0.99**	**0.99**	0.988	**0.99**	**0.99**		0.978	0.986	0.984	**0.99**	**0.99**
30%	0.954	0.95	0.94	0.968	**0.97**		0.92	0.948	0.938	0.96	**0.964**
50%	0.904	0.896	0.892	0.928	**0.936**		0.846	0.874	0.872	0.906	**0.928**
70%	0.836	0.814	0.83	0.866	**0.882**		0.73	0.77	0.776	0.824	**0.862**
90%	0.686	0.646	0.664	0.728	**0.774**		0.522	0.572	0.638	0.648	**0.738**
Missing rate	CESC						KIRP				
	SVD	TOBMI	Lasso	TDimpute-self	TDimpute		SVD	TOBMI	Lasso	TDimpute-self	TDimpute
10%	0.986	**0.988**	0.986	**0.988**	**0.988**		0.968	0.97	0.962	0.98	**0.984**
30%	0.94	0.944	0.938	0.96	**0.966**		0.92	0.924	0.9	0.942	**0.952**
50%	0.87	0.876	0.858	0.91	**0.928**		0.85	0.852	0.832	0.888	**0.914**
70%	0.758	0.746	0.76	0.814	**0.856**		0.764	0.75	0.75	0.804	**0.85**
90%	0.582	0.59	0.622	0.622	**0.748**		0.616	0.602	0.652	0.642	**0.744**
Missing rate	LGG						HNSC				
	SVD	TOBMI	Lasso	TDimpute-self	TDimpute		SVD	TOBMI	Lasso	TDimpute-self	TDimpute
10%	0.99	0.99	0.99	0.994	**1**		0.99	0.99	0.99	0.992	**0.994**
30%	0.966	0.964	0.948	0.976	**0.98**		0.958	0.956	0.94	0.972	**0.98**
50%	0.916	0.908	0.882	0.952	**0.956**		0.89	0.888	0.87	0.94	**0.952**
70%	0.814	0.816	0.806	0.898	**0.906**		0.778	0.776	0.77	0.856	**0.896**
90%	0.714	0.676	0.706	0.722	**0.77**		0.588	0.576	0.644	0.62	**0.762**
Missing rate	SKCM						BLCA				
	SVD	TOBMI	Lasso	TDimpute-self	TDimpute		SVD	TOBMI	Lasso	TDimpute-self	TDimpute
10%	0.99	0.994	0.992	**0.996**	**0.996**		0.99	0.99	0.99	**0.992**	**0.992**
30%	0.964	0.97	0.968	0.98	**0.982**		0.962	0.966	0.964	0.974	**0.978**
50%	0.894	0.91	0.918	0.942	**0.952**		0.92	0.922	0.926	0.944	**0.952**
70%	0.78	0.798	0.85	0.866	**0.9**		0.86	0.862	0.89	0.896	**0.916**
90%	0.65	0.64	0.712	0.676	**0.77**		0.726	0.726	0.8	0.786	**0.838**
Missing rate	STAD						KIRC				
	SVD	TOBMI	Lasso	TDimpute-self	TDimpute		SVD	TOBMI	Lasso	TDimpute-self	TDimpute
10%	0.992	0.992	0.99	**0.996**	**0.996**		0.98	0.974	0.972	**0.986**	**0.986**
30%	0.97	0.976	0.962	0.984	**0.986**		0.928	0.898	0.88	0.938	**0.948**
50%	0.94	0.952	0.912	0.964	**0.968**		0.852	0.792	0.752	0.866	**0.892**
70%	0.894	0.91	0.846	0.934	**0.94**		0.742	0.646	0.622	0.764	**0.822**
90%	0.78	0.774	0.738	0.832	**0.866**		0.56	0.518	0.516	0.56	**0.706**
Missing rate	COAD						SARC				
	SVD	TOBMI	Lasso	TDimpute-self	TDimpute		SVD	TOBMI	Lasso	TDimpute-self	TDimpute
10%	0.992	0.992	0.99	0.994	**0.996**		0.98	0.988	0.98	**0.99**	**0.99**
30%	0.968	0.97	0.954	0.978	**0.98**		0.946	0.958	0.942	0.964	**0.97**
50%	0.926	0.918	0.89	0.948	**0.954**		0.892	0.912	0.89	0.926	**0.934**
70%	0.856	0.852	0.83	0.902	**0.922**		0.82	0.838	0.836	0.86	**0.876**
90%	0.642	0.674	0.634	0.764	**0.856**		0.664	0.658	0.712	0.72	**0.772**

The results are averaged over 5 random replicas. Best results are highlighted in bold face.

Table S2.1. Overlap of top 100 methylation-driving genes from imputed dataset and full dataset

Missing rate	SVD	TOBMI	Lasso	TDimpute-self	TDimpute
10%	89.61*	87.66*	86.45*	91.59	**91.98**
30%	76.03*	71.83*	68.15*	83.14*	**84.00**
50%	62.44*	55.21*	52.43*	73.09*	**76.24**
70%	45.43*	37.70*	37.70*	57.96*	**65.66**
90%	24.83	18.48*	22.56*	26.73*	**50.43**

The results are averaged over 5 random replicas. Best results are highlighted in bold face.
* indicates statistical significance (p-value < 0.05) between TDimpute and other methods.

Table S2.2. Overlap of top 100 methylation-driving genes between imputed dataset and full dataset over 16 cancer types

Missing rate	BRCA					THCA				
	SVD	TOBMI	Lasso	TDimpute-self	TDimpute	SVD	TOBMI	Lasso	TDimpute-self	TDimpute
10%	96.2	93	92.8	95.8	**95.2**	**97.6**	96	94.8	97.4	**97.6**
30%	93.2	82.8	80.2	92.2	**93.4**	94	87.2	88.8	**94.8**	94.2
50%	86.8	69.4	71.8	**90.4**	88.8	88	68.2	77.2	89.8	**90.8**
70%	73.6	44	53.4	81.6	**85.8**	75.6	57.6	66.8	83.2	**85.4**
90%	34.6	9.6	21.4	45.6	**66.2**	34.2	31.6	41.2	52.4	**71**
Missing rate	PRAD					LUAD				
	SVD	TOBMI	Lasso	TDimpute-self	TDimpute	SVD	TOBMI	Lasso	TDimpute-self	TDimpute
10%	84.4	81	77.6	89	**90.2**	93.8	92.4	77.6	**94.6**	93.8
30%	61.8	59.4	54.6	**78.8**	77.4	84.4	82.4	54.6	87.2	**87.6**
50%	48.4	47.8	42.6	**66.2**	64.4	77.8	73.2	42.6	82.2	**83.6**
70%	38.6	37	35.2	51.2	**52.6**	63.8	63.4	35.2	73.8	**77.8**
90%	26.2	21.8	27	26.2	**36**	40.2	38.6	27	38.6	**68.4**
Missing rate	LIHC					LUSC				
	SVD	TOBMI	Lasso	TDimpute-self	TDimpute	SVD	TOBMI	Lasso	TDimpute-self	TDimpute
10%	85.6	87.2	91.4	91.4	**92**	94.6	93.2	93.6	94	**95.4**
30%	72.6	72.4	68.8	80.6	**81.6**	86.4	83.4	75	**89.6**	89
50%	64.8	53.2	50	72.4	**76.2**	75.6	71	55.8	82.6	**83.8**
70%	50	25.2	24.6	56.6	**60**	51.4	46.2	32	75.2	**78.4**
90%	27.4	8.2	11.6	31	**51.2**	18.2	16.2	13.6	23.2	**65**
Missing rate	CESC					KIRP				
	SVD	TOBMI	Lasso	TDimpute-self	TDimpute	SVD	TOBMI	Lasso	TDimpute-self	TDimpute
10%	89.2	82.4	81.2	**91**	90.2	83.2	79.4	78.6	84.8	**87.2**
30%	73.2	64.2	60.8	78.8	**82.2**	68.4	57.8	60.2	74	**78.2**
50%	47	40.8	44.6	57.6	**70.4**	53.2	45	50.2	59.6	**67**
70%	30.4	30.6	35.6	33.2	**54.2**	37.6	22.8	29.4	42.8	**54**
90%	18.2	16.4	20.4	17.8	**38.4**	16.2	10.6	17.2	12	**36.6**
Missing rate	LGG					HNSC				
	SVD	TOBMI	Lasso	TDimpute-self	TDimpute	SVD	TOBMI	Lasso	TDimpute-self	TDimpute
10%	81.6	81.8	77.2	88.4	**89.8**	91.6	90.6	86.8	**92.2**	92
30%	67.2	63.6	56.8	75.8	**79.2**	80	79.8	66.4	**84.6**	83.4
50%	59.8	57.4	52.6	64	**67.6**	66.2	66.6	44.8	77.6	**80.4**
70%	48.2	45.2	48.6	52.2	**53**	42.6	52.6	31.2	65.6	**72.8**
90%	33.2	21.4	38	26.4	**34.6**	18.2	30.6	16.4	22.2	**58.2**
Missing rate	SKCM					BLCA				
	SVD	TOBMI	Lasso	TDimpute-self	TDimpute	SVD	TOBMI	Lasso	TDimpute-self	TDimpute
10%	95.6	94.8	95.8	**96.4**	**96.4**	94.4	92.2	92	**95**	94.6
30%	86.2	83.6	88.6	91.4	**92.6**	85.6	78.6	75.4	**90.2**	90
50%	78.4	72	81.8	86.4	**89.6**	72	55.8	54.8	85.6	**86.8**
70%	60.4	47.2	68.2	71	**78.4**	51	36.2	37.2	72.2	**80**
90%	36	24.4	44.2	43.8	**64.2**	33.4	23.8	18	29.8	**67**
Missing rate	STAD					KIRC				
	SVD	TOBMI	Lasso	TDimpute-self	TDimpute	SVD	TOBMI	Lasso	TDimpute-self	TDimpute
10%	84.4	84.2	77.2	**87.4**	**87.4**	88.4	85.2	81	89.6	**91**
30%	69.4	69.8	60.8	**81**	79	71.4	66.6	56.6	74.8	**77.8**
50%	54.4	55.6	47.8	**69.4**	68.8	55.4	47.4	37.8	60.8	**65.6**
70%	36.4	41.6	37.8	55.2	**61.6**	36.8	30.8	25.6	45.4	**54**
90%	19.4	15.2	32	25.8	**44**	24.2	19.8	20.4	13.2	**39.2**
Missing rate	COAD					SARC				
	SVD	TOBMI	Lasso	TDimpute-self	TDimpute	SVD	TOBMI	Lasso	TDimpute-self	TDimpute
10%	83.4	81.4	82.6	**86.8**	86.4	89.8	87.8	91.2	91.6	**92.4**
30%	47.6	55	49.2	71.4	**72**	75	62.6	79.4	85	**86.4**
50%	19	31.2	23.8	54.8	**60.4**	52.2	28.8	58.6	70	**75.6**
70%	5.4	12.6	10	27.4	**42.6**	25	10.2	36.8	40.8	**60**
90%	3.4	2	3.2	1.2	**23.8**	14.2	5.4	6	18.4	**43**

The results are averaged over 5 random replicas. Best results are highlighted in bold face.

Table S3. PR-AUC for detecting significantly prognostic gene on imputed datasets over 16 cancer types.

Missing rate	BRCA					THCA				
	SVD	TOBMI	Lasso	TDimpute-self	TDimpute	SVD	TOBMI	Lasso	TDimpute-self	TDimpute
10%	0.892	0.918	0.91	0.932	**0.938**	0.878	0.884	0.882	0.9	**0.906**
30%	0.738	0.82	0.802	0.844	**0.852**	0.658	0.642	0.66	0.714	**0.718**
50%	0.548	0.664	0.63	0.71	**0.736**	0.466	0.498	0.514	0.556	**0.588**
70%	0.382	0.446	0.39	0.526	**0.542**	0.344	0.352	0.358	0.42	**0.468**
90%	0.306	0.358	0.294	0.376	**0.406**	0.184	0.166	0.17	0.178	**0.238**
Missing rate	PRAD					LUAD				
	SVD	TOBMI	Lasso	TDimpute-self	TDimpute	SVD	TOBMI	Lasso	TDimpute-self	TDimpute
10%	**0.936**	**0.936**	0.932	0.924	**0.936**	0.936	0.94	0.942	**0.952**	**0.952**
30%	0.65	0.648	0.648	0.638	**0.67**	0.814	0.846	0.83	0.87	**0.882**
50%	0.42	0.432	0.412	0.43	**0.46**	0.676	0.706	0.694	0.748	**0.764**
70%	0.294	0.3	0.282	0.3	**0.316**	0.536	0.564	0.544	0.596	**0.654**
90%	0.104	0.092	0.096	0.104	**0.122**	0.36	0.402	0.38	0.398	**0.504**
Missing rate	LIHC					LUSC				
	SVD	TOBMI	Lasso	TDimpute-self	TDimpute	SVD	TOBMI	Lasso	TDimpute-self	TDimpute
10%	0.84	0.832	0.834	0.856	**0.866**	0.822	0.838	0.834	**0.864**	0.86
30%	0.614	0.648	0.642	0.676	**0.704**	0.556	0.544	0.564	0.574	**0.602**
50%	0.44	0.476	0.47	0.508	**0.524**	0.376	0.346	0.37	0.4	**0.43**
70%	0.288	0.308	0.304	0.356	**0.418**	0.22	0.21	0.22	0.236	**0.27**
90%	0.162	0.146	0.154	0.178	**0.248**	0.122	0.106	0.118	0.132	**0.178**
Missing rate	CESC					KIRP				
	SVD	TOBMI	Lasso	TDimpute-self	TDimpute	SVD	TOBMI	Lasso	TDimpute-self	TDimpute
10%	0.932	0.938	0.944	0.946	**0.948**	0.938	0.956	0.944	0.956	**0.96**
30%	0.802	0.816	0.804	0.828	**0.842**	0.812	0.864	0.848	0.866	**0.884**
50%	0.662	0.666	0.66	0.694	**0.708**	0.752	0.754	0.728	0.758	**0.78**
70%	0.47	0.438	0.472	0.516	**0.534**	0.582	0.642	0.592	0.608	**0.658**
90%	0.26	0.23	0.256	0.27	**0.378**	0.352	0.386	0.374	0.376	**0.452**
Missing rate	LGG					HNSC				
	SVD	TOBMI	Lasso	TDimpute-self	TDimpute	SVD	TOBMI	Lasso	TDimpute-self	TDimpute
10%	0.988	0.99	0.99	**0.992**	**0.992**	0.922	0.944	0.944	0.948	**0.952**
30%	0.964	0.966	0.966	0.97	**0.97**	0.754	0.82	0.808	0.83	**0.84**
50%	0.926	0.924	0.928	0.932	**0.938**	0.572	0.66	0.642	0.688	**0.728**
70%	0.872	0.868	0.886	0.878	**0.886**	0.448	0.496	0.47	0.518	**0.57**
90%	0.796	0.728	0.788	0.756	**0.79**	0.292	0.268	0.27	0.31	**0.418**
Missing rate	SKCM					BLCA				
	SVD	TOBMI	Lasso	TDimpute-self	TDimpute	SVD	TOBMI	Lasso	TDimpute-self	TDimpute
10%	0.184	0.184	**0.924**	0.184	0.182	0.866	0.91	0.914	0.922	**0.926**
30%	0.182	0.184	**0.79**	0.182	0.182	0.592	0.732	0.738	0.764	**0.778**
50%	0.188	0.188	**0.67**	0.182	0.182	0.462	0.562	0.57	0.652	**0.68**
70%	0.188	0.188	**0.604**	0.182	0.18	0.342	0.412	0.434	0.496	**0.554**
90%	0.198	0.202	**0.404**	0.192	0.184	0.238	0.218	0.258	0.292	**0.424**
Missing rate	STAD					KIRC				
	SVD	TOBMI	Lasso	TDimpute-self	TDimpute	SVD	TOBMI	Lasso	TDimpute-self	TDimpute
10%	0.876	0.862	0.852	0.884	**0.886**	0.984	0.99	0.99	0.99	**0.992**
30%	0.544	0.6	0.58	0.65	**0.656**	0.944	0.962	0.962	0.966	**0.968**
50%	0.372	0.424	0.394	**0.46**	0.454	0.898	0.918	0.912	0.934	**0.938**
70%	0.158	0.224	0.202	0.246	**0.248**	0.802	0.84	0.826	0.872	**0.882**
90%	0.078	0.112	0.09	0.116	**0.132**	0.648	0.674	0.662	0.664	**0.726**
Missing rate	COAD					SARC				
	SVD	TOBMI	Lasso	TDimpute-self	TDimpute	SVD	TOBMI	Lasso	TDimpute-self	TDimpute
10%	0.778	0.804	0.816	0.824	**0.836**	0.926	0.936	0.932	0.94	**0.942**
30%	0.42	0.458	0.446	0.462	**0.512**	0.738	0.79	0.784	0.8	**0.816**
50%	0.202	0.228	0.224	0.23	**0.298**	0.552	0.614	0.616	0.618	**0.658**
70%	0.118	0.122	0.126	0.136	**0.194**	0.424	0.454	0.476	0.472	**0.528**
90%	0.058	0.058	0.058	0.064	**0.106**	0.246	0.29	0.292	0.316	**0.398**

The results are averaged over 5 random replicas. Best results are highlighted in bold face.

Table S4.1. Overlap of top 100 significantly prognostic genes identified by univariate Cox model between imputed datasets and full datasets.

Missing rate	SVD	TOBMI	Lasso	TDimpute-self	TDimpute
10%	73.5*	74.8*	68.69*	76.4*	**77.5**
30%	50.6*	52.3*	46.24*	56.6	**57.3**
50%	37.6*	37.8*	31.43*	44.2	**44.9**
70%	27.3*	26.6*	21.04*	33.2*	**35.2**
90%	15.8*	16*	11.59*	20.6*	**24.6**

The results are averaged over 5 random replicas. Best results are highlighted in bold face.
* indicates statistical significance (p-value < 0.05) between TDimpute and other methods.

Table S4.2. Overlap of top 100 prognostic genes identified by univariate Cox model between imputed dataset and full dataset over 16 cancer types.

Missing rate	BRCA					THCA				
	SVD	TOBMI	Lasso	TDimpute-self	TDimpute	SVD	TOBMI	Lasso	TDimpute-self	TDimpute
10%	63.4	70	67	**73**	72.4	69.2	68.2	71.2	70.2	**70.6**
30%	40.6	52.2	49.4	52.8	**54.8**	46	42.2	44.2	**49.4**	49
50%	27.2	33.8	28.4	36.2	**39.4**	31.2	28.2	29.4	33.4	**35.2**
70%	13.8	18	13	21.2	**24**	22.2	18.4	18.4	**25.2**	23.8
90%	6.2	5.8	4.8	9.4	**12.6**	**16.4**	8	7.2	12.8	15.4
Missing rate	PRAD					LUAD				
	SVD	TOBMI	Lasso	TDimpute-self	TDimpute	SVD	TOBMI	Lasso	TDimpute-self	TDimpute
10%	86	85.4	85.6	83.4	**85.6**	75.8	76.6	75	79.8	**82.8**
30%	51.4	49.2	50.6	52	**54.6**	58	53.8	53.4	**66**	64
50%	29.4	29	29.2	32.6	**33.4**	48	41.4	40.4	51.8	**53**
70%	18.6	19	16	19	**19.4**	33.2	27	26.4	37.8	**47.2**
90%	2.8	3.4	3.4	3.6	**4.4**	19.4	9.8	14	21	**32**
Missing rate	LIHC					LUSC				
	SVD	TOBMI	Lasso	TDimpute-self	TDimpute	SVD	TOBMI	Lasso	TDimpute-self	TDimpute
10%	65	64.2	63	67.8	**68**	59.8	63	63	63.4	**68.8**
30%	45.2	48	48.2	**53.2**	50.2	32.6	35.4	34.8	**38.6**	**38.6**
50%	29.8	32.2	33.4	**37**	34.2	20.4	20.2	20.2	23.6	**24.8**
70%	**23.2**	19	21.4	21.4	22.2	10.4	9.4	10	11	**14.2**
90%	7.2	4.6	5.8	8	**13**	2.2	2	2.8	2.8	**6**
Missing rate	CESC					KIRP				
	SVD	TOBMI	Lasso	TDimpute-self	TDimpute	SVD	TOBMI	Lasso	TDimpute-self	TDimpute
10%	81.6	79.6	82.8	80.2	**82.2**	88.6	87.8	77.4	87.8	**88.8**
30%	58.8	60.4	59.2	59	**63.6**	**85.2**	68	53.2	85	81
50%	43.6	47.2	47	48.4	**50.4**	**83.8**	55.6	36.8	82.2	77.6
70%	21.2	21.4	26	**31.2**	30.2	80.2	57.4	30.6	**83.2**	82
90%	4.4	5.4	5.4	8.4	**13**	49.4	49.6	26.8	59.4	**67.4**
Missing rate	LGG					HNSC				
	SVD	TOBMI	Lasso	TDimpute-self	TDimpute	SVD	TOBMI	Lasso	TDimpute-self	TDimpute
10%	90.2	89.6	76.4	91.2	**92.6**	69	70.6	71.8	**77.2**	**77.2**
30%	87.4	87.2	66.4	**88.6**	88	47	50.4	48.4	52.2	**56**
50%	88	86.4	55.2	**89**	88	28.2	33.6	31.2	37.6	**42.4**
70%	84.4	90.4	61.4	90.6	**91.2**	16.4	18.8	18	22.2	**28.4**
90%	80.8	85.4	65.2	**92.6**	90	6.4	3.6	4.4	7.2	**13.8**
Missing rate	SKCM					BLCA				
	SVD	TOBMI	Lasso	TDimpute-self	TDimpute	SVD	TOBMI	Lasso	TDimpute-self	TDimpute
10%	61	66.2	0	**66.4**	65.2	69.4	74.6	74.2	74.8	**75.6**
30%	30.2	37.2	0.8	37.4	**39.2**	37.6	47.6	49.4	**54.6**	54.2
50%	11.8	15.2	0.6	**21**	20.4	21.2	26.8	31	**41.8**	41.6
70%	3.2	6.2	0	8.6	**11.8**	14.6	15.2	18	27.2	**31.8**
90%	1.8	0.4	0	1.4	**4.4**	7.8	4.6	7.6	10.2	**20.6**
Missing rate	STAD					KIRC				
	SVD	TOBMI	Lasso	TDimpute-self	TDimpute	SVD	TOBMI	Lasso	TDimpute-self	TDimpute
10%	71.6	70.2	69	72.2	**73**	91.4	89.6	84.6	**91.4**	91.2
30%	37.8	42.4	42.2	44.4	**45.2**	75.8	72.6	55	81.8	**83.2**
50%	25.4	26.4	25.2	25.8	**29.6**	65.4	64.4	36.6	**81.2**	78.2
70%	8.2	9.4	7.2	12.2	**12.6**	57.8	58.6	32.8	**76.6**	72.4
90%	**3.8**	3.4	1.8	3.2	3.6	38.6	49.8	24.4	**63.6**	62
Missing rate	COAD					SARC				
	SVD	TOBMI	Lasso	TDimpute-self	TDimpute	SVD	TOBMI	Lasso	TDimpute-self	TDimpute
10%	59.8	63.4	62.2	64.8	**65.6**	73.4	77	75.8	79.4	**79.6**
30%	30.4	33	30.8	32.8	**37.8**	45.8	57.2	53.8	57.8	**58**
50%	18.4	**21**	20.4	19.2	20.6	30	43	37.8	46	**49**
70%	7.8	6.6	8.4	9.2	**15**	20.8	30	29	35.2	**37.2**
90%	2	2.4	1.4	2.4	**8.2**	3.2	18.2	10.4	23.2	**27**

The results are averaged over 5 random replicas. Best results are highlighted in bold face.

Table S5. The enrichment factors of the top 100 ranked genes in the gene list from The Human Protein Atlas across 16 cancer types

Missing rate	BRCA						THCA				
	SVD	TOBMI	Lasso	TDimpute-self	TDimpute		SVD	TOBMI	Lasso	TDimpute-self	TDimpute
10%	7.637	8.625	8.098	8.493	**8.888**		8.664	9.102	**9.322**	8.554	9.102
30%	4.411	**6.584**	5.925	6.452	6.386		5.045	6.251	6.141	**6.799**	6.361
50%	2.765	4.740	3.489	**5.267**	**5.267**		3.400	4.058	4.167	**4.496**	**4.496**
70%	1.383	2.436	1.185	3.226	**3.424**		2.303	2.961	2.632	**3.509**	2.522
90%	0.790	0.988	0.593	1.580	**1.843**		1.755	1.206	0.987	**2.303**	1.864
Missing rate	PRAD						LUAD				
	SVD	TOBMI	Lasso	TDimpute-self	TDimpute		SVD	TOBMI	Lasso	TDimpute-self	TDimpute
10%	14.60	**15.08**	**15.08**	13.40	14.12		8.476	8.476	8.242	**8.944**	**8.944**
30%	8.86	**9.57**	9.33	8.86	8.14		6.839	6.079	5.962	**7.541**	**7.541**
50%	4.31	**5.74**	5.50	4.55	4.55		5.436	4.618	4.559	5.904	**5.962**
70%	3.11	**4.79**	3.35	3.59	2.15		3.566	2.923	3.040	4.150	**5.904**
90%	0.96	1.68	1.44	**2.15**	1.44		2.455	0.877	1.695	2.747	**4.092**
Missing rate	LIHC						LUSC				
	SVD	TOBMI	Lasso	TDimpute-self	TDimpute		SVD	TOBMI	Lasso	TDimpute-self	TDimpute
10%	1.004	0.951	0.977	1.017	**1.030**		1.403	1.637	1.461	1.578	**1.695**
30%	**0.700**	0.647	0.687	**0.700**	0.674		0.760	1.169	0.877	1.286	**1.461**
50%	0.396	0.449	**0.489**	0.436	0.370		0.526	0.760	0.468	0.701	**0.818**
70%	0.304	0.225	**0.330**	0.225	0.277		0.292	**0.526**	0.234	0.292	0.468
90%	**0.119**	0.000	0.066	0.092	**0.119**		0.000	**0.058**	0.000	0.000	**0.058**
Missing rate	CESC						KIRP				
	SVD	TOBMI	Lasso	TDimpute-self	TDimpute		SVD	TOBMI	Lasso	TDimpute-self	TDimpute
10%	13.09	13.19	13.30	13.19	**13.51**		0.363	0.363	0.300	**0.370**	0.363
30%	9.62	10.62	9.88	10.04	**10.83**		**0.351**	0.287	0.223	**0.351**	0.332
50%	6.73	8.15	7.78	7.99	**8.83**		**0.370**	0.236	0.134	**0.370**	0.338
70%	2.58	3.68	3.94	4.73	**5.10**		0.332	0.300	0.147	**0.363**	0.338
90%	0.47	0.68	0.95	1.31	**2.05**		0.217	0.217	0.108	0.255	**0.281**
Missing rate	LGG						HNSC				
	SVD	TOBMI	Lasso	TDimpute-self	TDimpute		SVD	TOBMI	Lasso	TDimpute-self	TDimpute
10%	NA						6.29	6.92	6.97	**7.45**	7.40
30%							3.84	4.71	4.71	5.00	**5.53**
50%							1.97	3.32	2.64	3.80	**4.23**
70%							0.96	1.63	1.35	2.21	**2.64**
90%							0.34	0.19	0.24	0.72	**1.01**
Missing rate	SKCM						BLCA				
	SVD	TOBMI	Lasso	TDimpute-self	TDimpute		SVD	TOBMI	Lasso	TDimpute-self	TDimpute
10%	2.78	**3.53**	0	2.78	3.16		6.65	7.24	7.45	7.28	**7.56**
30%	1.11	**2.41**	0	2.23	1.86		3.13	4.94	4.91	**5.74**	5.71
50%	0.19	1.11	0	0.93	**1.49**		1.67	2.82	2.85	**4.42**	4.14
70%	0.00	0.74	0	0.00	**1.11**		0.91	1.57	1.85	2.79	**3.31**
90%	0.00	0.00	0	0.00	**0.37**		0.59	0.35	0.59	0.97	**2.26**
Missing rate	STAD						KIRC				
	SVD	TOBMI	Lasso	TDimpute-self	TDimpute		SVD	TOBMI	Lasso	TDimpute-self	TDimpute
10%	5.09	5.09	5.09	**5.47**	5.35		0.281	0.281	0.268	**0.287**	**0.287**
30%	2.80	2.80	2.67	3.18	**3.69**		0.255	0.261	0.166	0.255	**0.268**
50%	0.89	1.65	1.40	1.53	**1.78**		0.210	0.223	0.077	0.268	**0.274**
70%	0.00	0.38	0.51	**0.89**	0.76		0.185	0.204	0.089	0.249	**0.268**
90%	0.00	0.13	0.13	0.00	**0.25**		0.096	0.185	0.038	0.217	**0.236**
Missing rate	COAD						SARC				
	SVD	TOBMI	Lasso	TDimpute-self	TDimpute		SVD	TOBMI	Lasso	TDimpute-self	TDimpute
10%	1.02	1.09	1.09	1.15	**1.28**		NA				
30%	0.77	**0.83**	**0.51**	0.70	0.77						
50%	0.32	0.45	0.26	**0.51**	0.32						
70%	0.00	0.00	0.00	0.00	**0.19**						
90%	0.06	0.06	0.00	0.06	**0.13**						

The results are averaged over 5 random replicas. Best results are highlighted in bold face.

LGG and SARC are not included in this scenario, because no relavent gene list provided by The Human Protein Atlas.

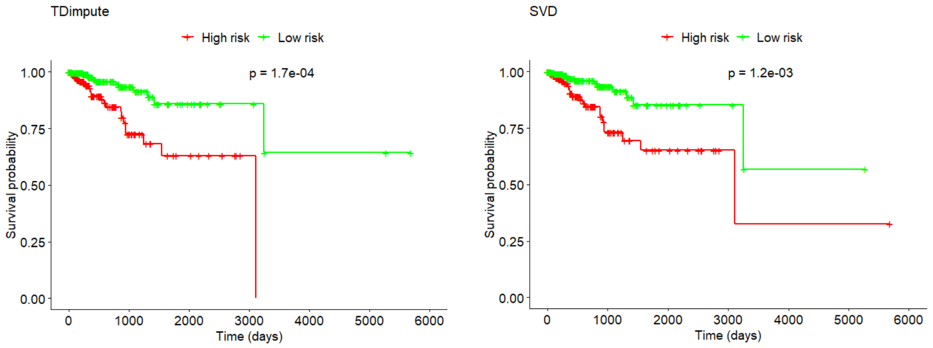

Fig S7. Kaplan-Meier plot for the two clusters obtained from the UCEC dataset imputed by TDimpute and SVD, respectively.

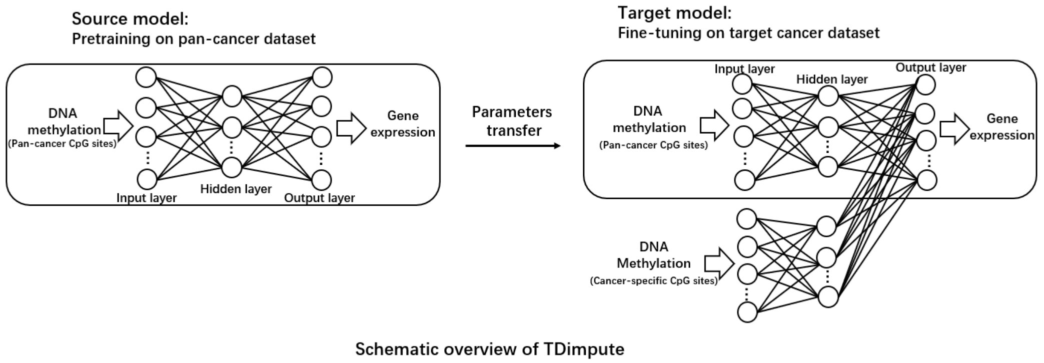

Fig S8. The architecture of transfer learning based neural network (TDimpute) with cancer-specific CpG sites as auxiliary input. The input at fine-tuning stage consists of two parts: the cancer-specific part takes the highly variable CpG sites from the target cancer as input, and the transfer part takes the commonly variable CpG sites from pan-cancer dataset as input.

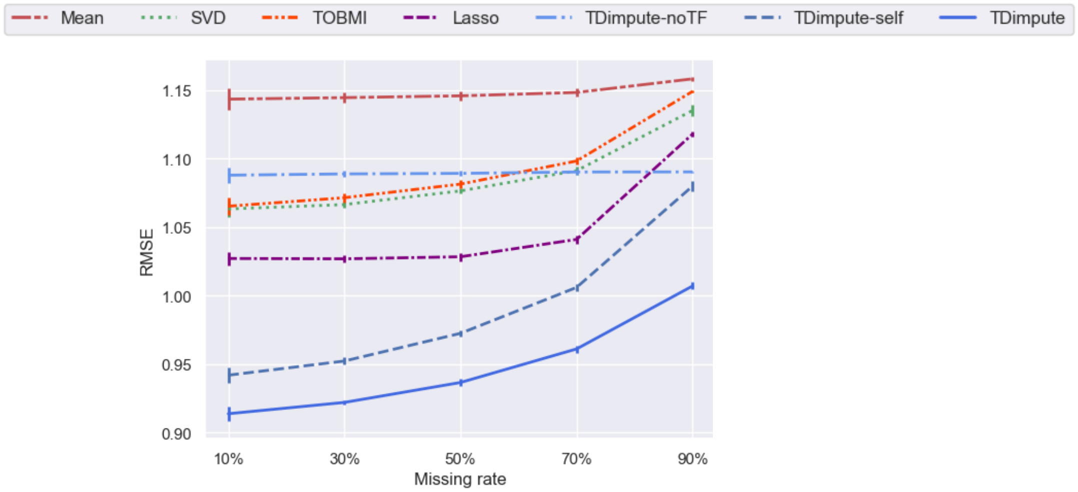

Fig S9. RMSE values of each imputation method with top 20000 CpG sites as input. For SVD, TOBMI, Lasso and TDimpute-self, the top 20000 variable cancer-specific CpG sites are used as input, while the input of TDimpute includes both the pan-cancer and cancer-specific CpG sites. Results were averaged across 16 imputed cancer datasets. The error bar shows the standard deviation.

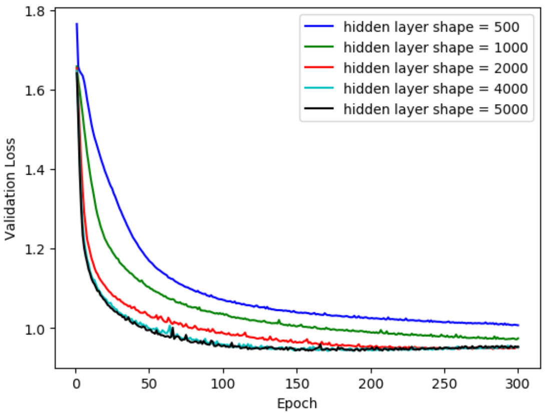

RMSE for cross-validation (5CV) when using different hyper-parameters.

the number of hidden layers	hidden layer shape	epoch 50	100	150	**300**	500
**1**	500	1.253	1.131	1.09	1.052	1.04
	1000	1.146	1.076	1.047	1.016	1.013
	2000	1.074	1.029	1.009	0.995	1.009
	**4000**	1.043	1.003	0.991	0.997	1.015
	5000	1.035	0.999	0.988	0.995	1.015
3	(4000, 500, 4000)	1.13	1.067	1.039	1.01	1.011
	(4000, 1000, 4000)	1.107	1.046	1.032	1.011	1.035
	(4000, 2000, 4000)	1.092	1.042	1.021	1.025	1.064
	(5000, 500, 5000)	1.085	1.034	1.021	1.028	1.078
	(5000, 1000, 5000)	1.097	1.052	1.025	1.014	1.04
	(5000, 2000, 5000)	1.124	1.062	1.036	1.008	1.012

Seleced hyper-parameters are highlighted in bold face.

Fig S10 (left). Loss curves for different hidden layer shape and **Table S6** (right). hyper-parameter analysis for hidden layer shape, the number of hidden layers, and training epochs on pan-cancer dataset (excluding BRCA dataset).

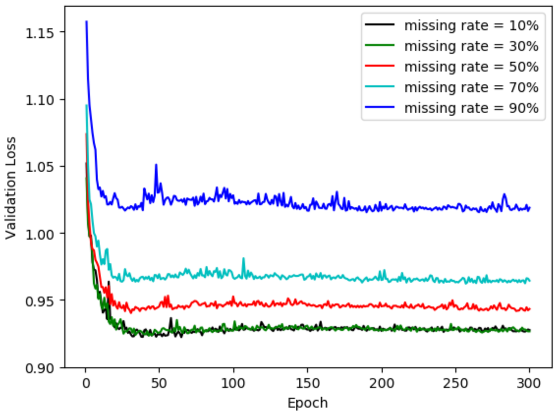

Fig S11. The loss curves of different missing rates on the validation dataset of BRCA.
