# Supplementary material for: Imputing missing RNA-sequencing data from DNA methylation by using a transfer learning–based neural network
Source: Gigascience. 2020 Jul 10;9(7):giaa076. doi: 10.1093/gigascience/giaa076 (PMC7350980; doi:10.1093/gigascience/giaa076)
Supplement: giaa076_Response_to_Reviewer_Comments_Original_Submission [file giaa076_response_to_reviewer_comments_original_submission.pdf]

Author's Response To Reviewer Comments

Close

Response to the editor:
1. The rationale is not clear and needs further clarification as per reviewers' suggestions, as well as more clarification regarding the split between the training and the test datasets.
RE: See below for the response to reviewers.

2. The writing also needs to be improved, so we strongly suggest you hire a professional native English speaking editorial service to edit the revised manuscript.
RE: We have carefully revised the manuscript accordingly.

3. The reviewer reports, together with any other comments, are below. Please also take a moment to check our website at https://www.editorialmanager.com/giga/ for any additional comments that were saved as attachments.
RE: See below for the point-to-point response.

4. In addition, please register any new software application in the bio.tools and SciCrunch.org databases to receive RRID (Research Resource Identification Initiative ID) and biotoolsID identifiers, and include these in your manuscript. This will facilitate tracking, reproducibility and re-use of your tool.
RE: We have registered our tools in bio.tools with biotoolsID: TDimpute, and in SciCrunch.org with RRID: SCR_018306. These two IDs have been included in section "Availability of source code and requirements" of the revised manuscript.

5. Please include a point-by-point within the 'Response to Reviewers' box in the submission system. Please ensure you describe additional experiments that were carried out and include a detailed rebuttal of any criticisms or requested revisions that you disagreed with. Please also ensure that your revised manuscript conforms to the journal style, which can be found in the Instructions for Authors on the journal homepage. If the data and code has been modified in the revision process please be sure to update the public versions of this too.
RE: We have updated the codes in the GitHub: https://github.com/sysu-yanglab/TDimpute and the datasets in Synapse with ID: syn21438134, https://www.synapse.org/#!Synapse:syn21438134. We have also revised manuscript to conforms to the journal style.

Reviewer reports:

Reviewer #1: In this manuscript, Zhou et al. have used a 3-layer neural network for imputation of gene expression profiles from DNA methylation data.
While the study seems interesting, there are several points to consider before publication:

Response to Reviewer 1

Major points:
1. The rational behind selecting a subset of data in some points is not very clear:
- For instance, Fig. 5 shows a Kaplan-Meier plot for *Wilms-tumor* dataset, which shows a significant advantage of TDIMpute vs. *Lasso* in distinguishing between high-risk and low-risk patients. The same figure in the preprint of the same manuscript (https://www.biorxiv.org/content/10.1101/803692v1.full.pdf), however, has compared TDImpute vs. *TOBMI* on *UCEC* dataset, which shows much similar results between both methods. I'm afraid some parts of results are *cherry-picked* in a way that shows a great advantage of TDImpute over the other methods, which might not be a fair comparison.
RE:
We thank the reviewer for this comment. We chose the UCEC dataset since it has the largest portion of mRNA-missing samples (172 samples with RNA-seq and 267 without, missing rate = 61%) to evaluate

the impact of imputation on downstream analysis. For the same reason to challenge our method, we chose the TARGET project for independent set, and the Wilms tumor dataset was selected because it has the smallest sample size in the dataset. For a comprehensive comparison, we now put both the UCEC and Wilms tumor into the updated Validation on the UCEC from TCGA dataset section and Independent test on the TARGET dataset section, respectively. The selections of the datasets were added as follows:
“As a real data application, we performed survival analysis on the Uterine corpus endometrial carcinoma (UCEC) because it has the largest proportion of missing gene expression in the TCGA (172 samples with RNA-seq and 267 without).” (In Validation on the UCEC from TCGA dataset section)
“We chose the Wilms tumor (the most common type of childhood kidney cancer) as it has the smallest sample size.” (In Datasets and preprocessing section)

2. Fig. 4 of the submitted manuscript has also a notable difference from Fig. 4 of the pre-print. While Fig. 4A seems to depict the same results between two versions (excepting added Lasso method in the manuscript), Fig. 4B shows a completely different trend for TDImpute which is (expectedly) decreasing in the manuscript, but has been increasing in the pre-print.
For above reasons, I suggest the authors will share the whole source-code and pre-processed datasets in a way that reproducing the whole set of results is possible very conveniently.
RE:
We thank the reviewer for pointing out the issue on Fig 4B. The difference between Fig 4B from the submitted manuscript and the pre-print was due to the different inputted genes for the Cox model. In the pre-print, the top 100 significantly related genes were used, while the significantly related genes ($p \leq 0.05$) were used in the submitted manuscript. We made this change since this could achieve slightly higher C-index at low missing rate. The increase may be due to the uncertainty in building the Cox model. With contributions from multiple gene features, the models could be relatively robust to some genes which are not well imputed.
We have also uploaded the whole source-code for reproducing the results to GitHub: https://github.com/sysu-yanglab/TDimpute. The pre-processed datasets (including UCEC dataset) have been uploaded to Synapse with ID: syn21438134, https://www.synapse.org/#!Synapse:syn21438134.

2. It's not clear why the authors have not used deep neural networks, while those networks are shown to outperform 3-layer perceptrons in many applications.
RE:
We thank the reviewer for raising this important point about deeper network. We have performed hyper-parameters analysis with deeper network of 3 hidden layers and found that more layers did not lead to better imputation accuracy (Table S6). The reason may be that with limited training samples (thousands of samples), deeper networks mean more parameters (hundreds of millions of parameters) and overfit more easily. Consequently, we chose a simple network based on a principle of parsimony. The procedure of hyperparameter tuning has been added in the “Hyper-parameters tuning” part of Network Architecture and model training section, as following: “Finally, we selected the following hyper-parameters for pan-cancer model: 1 hidden layer (selected from 1 and 3) including 4000 nodes (from 500, 1000, 2000, 4000, and 5000), Sigmoid activation function (from Tanh, Relu, and Sigmoid), epochs of 300 (from 50, 100, 150, 300, and 500), and batch size of 128. For the fine-tuning stage, 150 epochs (from 50, 100, 150, 300, and 500) were used and the batch size was set as 16 because of small training dataset under large missing rate.”

3. RMSE around 1, while is shown to be better than other methods, is too high for gene expression profiles.
RE:
We thank the reviewer for this comment. The RMSE is somewhat dominated by big deviations. When evaluated by the mean absolute error, the value dropped to [0.616, 0.685] (Fig S1) that is in reasonable range. More importantly, the imputations led to reasonable results in the downstream analyses. For example, the results of methylation-expression correlations at missing rate 10% are close to the reference (R^2: 0.680 to 0.682 (baseline), overlap of top 100 methylation-driving gene: 90.76 to 100). In the revised manuscript, we have added the results of mean absolute error as the second paragraph in Comparisons on the imputation accuracy section.

Minor points:

4. On page two of the file, there is some information about the history of deep learning and papers which used variational types of deep learning (autoencoders, etc.) but I think maybe the authors should've focused on the previous works done in the area of transfer learning and not deep learning in general as what they've done by referencing to two transfer learning papers in CS. Also, I believe that

mentioning the names of the 16 cancer types that were used in this experiment in the introduction part would help the readers, who want to find the papers that were done on some specific types of cancer, a lot.
RE:
We thank the reviewer for these two suggestions and have updated the fourth paragraph in the Background section with more references about the deep transfer learning, as following:
“In the computer vision community, a common strategy is to pretrain the convolutional neural network (CNN) with ImageNet [15] and then fine-tune its last few layers or all layers (depend on the size of the target dataset) for the target tasks. This pretraining approach has achieved state-of-the-art results on many tasks including object detection [16], image segmentation [17], image classification [18], action recognition [19]. Yosinski et al. pointed out the relationship between network structure and the transferability of features [20]. They showed that the deep features transition from general to specific along the network, and transferring higher layers result in significant drop in performance since the features are more specific to source datasets.”
At the end of the Background section, the names of the 16 cancer types were also included in Table 1 accordingly.

5. Removing sites with low-variance DNA methylation over all samples might remove some sites that are hypo-methylated in one or two tissues/cell types and hyper-methylated in other tissues. Those sites might be required for correct RNA-seq imputation in those cell types.
RE:
We thank the reviewer for this advice. We have performed experiments by including specific methylation sites for each cancer type, but the results don’t show significant changes. We have added the discussions:
“In the preprocessing step, we removed sites with low-variance DNA methylation over all samples, which might remove some sites that are useful for specific cancer types. To test whether the cancer-specific CpG sites from the pan-cancer dataset are necessary for gene expression prediction, we updated the neural networks by taking the top 20000 variable CpG sites specific to each cancer as auxiliary input (Fig S8). Fig S9 shows that the improvement is relatively limited with only 0.4%-1.4% decrease of RMSE, compared to the network without including the cancer-specific sites. These results indicate that the CpG sites with high variances (> 0.05) over all samples are adequate enough for predicting gene expression across different cancers.”

5. Although they completely explained the data preparation phase, they did not provide much information about their network under the part "Neural network architecture", the number of layers and neurons in each layer, the activation functions in detail, the accuracy plots or anything else. Instead, they defined this information in the "missing data simulations" part. There is only some general information in the Neural net arch part which I believe that every person with a deep learning background already knows them and they don't need to remind them.
RE:
We thank the reviewer for this suggestion. We have updated the network architecture section with new section: Network architecture and model training. It contains the following three parts:
i> Neural network architecture part describes the basic information of the network including the number of layers, size of hidden layer, the activation functions, and loss function. We removed the common knowledge of deep learning to make it concisely.
ii> Transfer learning setting part describes the details of transfer learning using pan-cancer data, and the steps of pretraining and fine-tune.
iii> Hyper-parameters tuning part describes the analysis of hyper-parameter including the number of hidden layers, the hidden layer size, and the epochs to stop training.
The missing data simulations section was reorganized as Training and testing dataset section, describing the division of training and testing dataset during the pretrain and fine-tune stage.

6. Results section contains irrelevant information - e.g. review of the other methods. Some sentences like "our work is better than them" or "their work is worse than us" are not the best phrased.
RE:
We thank the reviewer for this suggestion and have moved the review of the other methods to Methods section. We also followed the advice to rephrase the improper sentences throughout the manuscript.

7. Increased R2 for TDImpute in Fig. 3 is unexpected.
RE:
We thank the reviewer for pointing out the unexpected trend of TDimpute in Fig 3. It’s a bug in the code for this experiment that ignored the strong but negative correlations in defining benchmark CpG-gene

pairs. We have fixed it (Fig 3) and updated the description as follows:
“As shown in Fig 3 (Fig S4 for each dataset), all imputation methods cause decreases in the correlations, and differences raise with the missing rates. In general, the TDimpute has the highest recovery power for the methylation-expression correlation with R^2 values close to the actual correlation (R^2=0.68).”

Reviewer #2: In this work, the authors presented a transfer learning-based neural network model to impute the missing RNA-Seq data. The omics data of the Cancer Genome Atlas (TCGA) database was obtained and used for the model training and testing. The author conducted various experiments and comparisons to demonstrate their model's performance. To merit the publication in GigaScience, the authors need to considerably improve this work in line with my major and minor comments below.

Response to Reviewer 2

Comments:
1. The data split for training and the testing dataset is not clearly described. In traditional transfer learning, base-model is usually trained based on the source domain dataset and then transferred to the target domain for the model fine-tune. Another testing dataset will be used to evaluate the performance of the transfer learning model. When using the TCGA data, the authors providing many performance results, while the details of how to get those are not clarified.
RE:
Thanks for reminding us to clarify the split for the training and testing dataset. We have updated the Training and testing dataset section to respond to this comment as follows:
“In the transfer learning for each target cancer type of TCGA, 32 cancer types in the TCGA dataset (except the target cancer type) was used as the source domain dataset for pretraining a model. During the test, in order to simulate performances under different missing rates, we used five fractions (10%, 30%, 50%, 70%, 90%). At each missing rate, we randomly selected one fraction (i.e. 1.0 - missing rate) of the target cancer samples to fine-tune the model that were tested on the left samples to predict the gene expression. The predicted values were then compared with the actual values to evaluated the model performances.”

2. As for the model performance comparison, the survival analysis and c-index were used very often. Along with all the compared tools and methods, the author should also provide the survival analysis using the TNM cancer typing system or cancer stages as then benchmark. Apart from that, the authors only tested their model on the TCGA datasets, which can properly demonstrate the model's generalization;
RE:
We have followed the suggestion and added the C-index of the four stages from the TNM cancer typing system, as following:
“For reference, the C-index was 0.669 according to the TNM typing system [26] that were decided by clinicians by tumor phenotypes, indicating the necessity to combine genotype and phenotype for survival analyses in future.” (in Impacts on the performances of clustering analysis and survival analysis section)
As independent test, we have included the analysis on the Wilms tumor dataset from the TARGET project in Independent test on the TARGET dataset section: “As an independent test beyond TCGA, we selected the smallest Wilams tumor from TARGET project. By fine-tuning the TCGA pan-cancer model with randomly selected 59 samples (50% of the dataset), the model was tested on the left samples. As expected, TDimpute achieved the lowest RMSE of 0.955 (TDimpute-self: 0.98; SVD: 1.064; Lasso: 1.006; TOBMI: 1.018). This demonstrates the generalization of the TCGA-pretrained model on independent dataset.”

3. Typo in the 5th row of Table S1, the cancer type should be "LGG", not "LGG_";
RE:
We thank the reviewer pointing out this typo. We have corrected it accordingly.

4. The English writing should be further improved, such as:
a. In the 4th line in the section of Abstract, it should be "Imputations from one type of omics data to another are becoming important", not "is becoming…";

b. In the 7th line in the section of Abstract, it should be "utilized for training".
RE:
We thank the reviewer for pointing out the mistakes. We have corrected them accordingly, and carefully revised the manuscript.

Close
