# Supplementary material for: Imputing missing RNA-sequencing data from DNA methylation by using a transfer learning–based neural network
Source: Gigascience. 2020 Jul 10;9(7):giaa076. doi: 10.1093/gigascience/giaa076 (PMC7350980; doi:10.1093/gigascience/giaa076)
Supplement: giaa076_Response_to_Reviewer_Comments_Revision_1 [file giaa076_response_to_reviewer_comments_revision_1.pdf]

Author's Response To Reviewer Comments

Close

Response to the editor:
Your manuscript "Imputing missing RNA-seq data from DNA methylation by using transfer learning based neural network" (GIGA-D-19-00438R1) has been assessed by our reviewers. Based on these reports, and my own assessment as Editor, I am pleased to inform you that it is potentially acceptable for publication in GigaScience, once you have carried out some essential revisions suggested by our reviewers.
RE: We thank you and the two reviewers for spending time reviewing our manuscript. Below we provide the response to reviewers.

Their reports, together with any other comments, are below. Please also take a moment to check our website at https://www.editorialmanager.com/giga/ for any additional comments that were saved as attachments.
RE: See below for the point-to-point response.

In addition, please register any new software application in the bio.tools and SciCrunch.org databases to receive RRID (Research Resource Identification Initiative ID) and biotoolsID identifiers, and include these in your manuscript. This will facilitate tracking, reproducibility and re-use of your tool.
RE: We have registered our tools in bio.tools with biotoolsID: TDimpute, and in SciCrunch.org with RRID: SCR_018306. These two IDs have been included in section "Availability of source code and requirements" of the revised manuscript.

Please include a point-by-point within the 'Response to Reviewers' box in the submission system. Please ensure you describe additional experiments that were carried out and include a detailed rebuttal of any criticisms or requested revisions that you disagreed with. Please also ensure that your revised manuscript conforms to the journal style, which can be found in the Instructions for Authors on the journal homepage. If the data and code has been modified in the revision process please be sure to update the public versions of this too.
RE: No additional experiments were carried out. The data and code are unchanged. We have also revised manuscript to conforms to the journal style.

Reviewer reports:
Reviewer #2: In this revised version, the authors have address most of the reviewer's points well. However, it remains unclear how exactly they randomly selected the fraction of the target samples from the dataset for fine-tuning and testing. What effect such fraction would impact on the performance of their proposed approach?
RE: We thank the reviewer for this comment. We have updated the Training and testing dataset section to explain the way we randomly selected samples for fine-tuning and testing as follows:
"…During the test, in order to simulate the performances under different missing rates, we used five fractions (10%, 30%, 50%, 70%, 90%). At each missing rate, we used the sample function (without replacement) in Pandas [40] to get one fraction (i.e. 1.0 - missing rate) of the target cancer samples as training set and the left samples as testing set with gene expression removed. On the training set, we fine-tuned the model that were then tested on the testing set to predict the gene expression."

We also updated the Comparisons on the imputation accuracy section to describe the effect of missing rates on the performance:
"…By comparison, TDimpute-self (indicates the TDimpute trained and predicted on the target cancer dataset) without using transfer learning yielded 2%-9% lower RMSE than Lasso from the highest (90%) to lowest missing rate (10%). The small improvement at the highest missing rate is because that the limited training data can't provide enough information for the model to learn the methylation-expression correlations. This performance degradation at the highest missing rate is reduced by TDimpute which yielded 7% lower RMSE than Lasso, indicating the advantage of using transfer learning over non-transfer method."

Close
