# Supplementary material for: Imputing missing RNA-sequencing data from DNA methylation by using a transfer learning–based neural network
Source: Gigascience. 2020 Jul 10;9(7):giaa076. doi: 10.1093/gigascience/giaa076 (PMC7350980; doi:10.1093/gigascience/giaa076)
Supplement: giaa076_Reviewer_1_Report_Original_Submission — Ali Sharifi-Zarchi -- 2/18/2020 Reviewed [file giaa076_reviewer_1_report_original_submission.pdf]

Reviewer Report

Title: Imputing missing RNA-seq data from DNA methylation by using transfer learning based neural network

Version: Original Submission **Date:** 2/18/2020

Reviewer name: Ali Sharifi-Zarchi

Reviewer Comments to Author:

In this manuscript, Zhou et al. have used a 3-layer neural network for imputation of gene expression profiles from DNA methylation data.
While the study seems interesting, there are several points to consider before publication:
Major points:
1. The rational behind selecting a subset of data in some points is not very clear:
- For instance, Fig. 5 shows a Kaplan-Meier plot for *Wilms-tumor* dataset, which shows a significant advantage of TDIMpute vs. *Lasso* in distinguishing between high-risk and low-risk patients. The same figure in the preprint of the same manuscript (https://www.biorxiv.org/content/10.1101/803692v1.full.pdf), however, has compared TDImpute vs. *TOBMI* on *UCEC* dataset, which shows much similar results between both methods. I'm afraid some parts of results are *cherry-picked* in a way that shows a great advantage of TDImpute over the other methods, which might not be a fair comparison.
- Fig. 4 of the submitted manuscript has also a notable difference from Fig. 4 of the pre-print. While Fig. 4A seems to depict the same results between two versions (excepting added Lasso method in the manuscript), Fig. 4B shows a completely different trend for TDImpute which is (expectedly) decreasing in the manuscript, but has been increasing in the pre-print.
For above reasons, I suggest the authors will share the whole source-code and pre-processed datasets in a way that reproducing the whole set of results is possible very conveniently.
2. It's not clear why the authors have not used deep neural networks, while those networks are shown to outperform 3-layer perceptrons in many applications.
3. RMSE around 1, while is shown to be better than other methods, is too high for gene expression profiles.
Minor points:
- On page two of the file, there is some information about the history of deep learning and papers which used variational types of deep learning (autoencoders, etc.) but I think maybe the authors should've focused on the previous works done in the area of transfer learning and not deep learning in general as what they've done by referencing to two transfer learning papers in CS. Also, I believe that mentioning the names of the 16 cancer types that were used in this experiment in the introduction part would help the readers, who want to find the papers that were done on some specific types of cancer, a lot.
- Removing sites with low-variance DNA methylation over all samples might remove some sites that are hypo-methylated in one or two tissues/cell types and hyper-methylated in other tissues. Those sites might be required for correct RNA-seq imputation in those cell types.

- Although they completely explained the data preparation phase, they did not provide much information about their network under the part "Neural network architecture", the number of layers and neurons in each layer, the activation functions in detail, the accuracy plots or anything else. Instead, they defined this information in the "missing data simulations" part. There is only some general information in the Neural net arch part which I believe that every person with a deep learning background already knows them and they don't need to remind them.
- Results section contains irrelevant information - e.g. review of the other methods. Some sentences like "our work is better than them" or "their work is worse than us" are not the best phrased.
- Increased R2 for TDImpute in Fig. 3 is unexpected.

Methods

Are the methods appropriate to the aims of the study, are they well described, and are necessary controls included? Choose an item.

Conclusions

Are the conclusions adequately supported by the data shown? Choose an item.

Reporting Standards

Does the manuscript adhere to the journal's guidelines on minimum standards of reporting? Choose an item.

Choose an item.

Statistics

Are you able to assess all statistics in the manuscript, including the appropriateness of statistical tests used? Choose an item.

Quality of Written English

Please indicate the quality of language in the manuscript: Choose an item.

Declaration of Competing Interests

Please complete a declaration of competing interests, considering the following questions:

- Have you in the past five years received reimbursements, fees, funding, or salary from an organisation that may in any way gain or lose financially from the publication of this manuscript, either now or in the future?
- Do you hold any stocks or shares in an organisation that may in any way gain or lose financially from the publication of this manuscript, either now or in the future?

- Do you hold or are you currently applying for any patents relating to the content of the manuscript?
- Have you received reimbursements, fees, funding, or salary from an organization that holds or has applied for patents relating to the content of the manuscript?
- Do you have any other financial competing interests?
- Do you have any non-financial competing interests in relation to this paper?

If you can answer no to all of the above, write 'I declare that I have no competing interests' below. If your reply is yes to any, please give details below.

No to all above questions

I agree to the open peer review policy of the journal. I understand that my name will be included on my report to the authors and, if the manuscript is accepted for publication, my named report including any attachments I upload will be posted on the website along with the authors' responses. I agree for my report to be made available under an Open Access Creative Commons CC-BY license (http://creativecommons.org/licenses/by/4.0/). I understand that any comments which I do not wish to be included in my named report can be included as confidential comments to the editors, which will not be published.

Choose an item.

To further support our reviewers, we have joined with Publons, where you can gain additional credit to further highlight your hard work (see: https://publons.com/journal/530/gigascience). On publication of this paper, your review will be automatically added to Publons, you can then choose whether or not to claim your Publons credit. I understand this statement.

Yes Choose an item.
